# Global, Regional, and National Burden of Oral Diseases in Older Adults Aged 65 Years And Over

**DOI:** 10.1016/j.identj.2025.109297

**Published:** 2025-12-09

**Authors:** Wei Lu, Wanqing Du, Xuejing Duan

**Affiliations:** a Department of Periodontology, Shandong Provincial Hospital Affiliated to Shandong First Medical University, Jinan, Shandong, China; b School of Stomatology, Shandong First Medical University, Jinan, Shandong, China

**Keywords:** Global Burden of Disease Study, Oral diseases, Dental caries, Edentulism, Periodontal disease, Older adults

## Abstract

**Objectives:**

This study focuses on examining worldwide trends in oral disease burden, including periodontal disease, dental caries, edentulism, and other oral disorders, among those aged 65 years and above between 1990 and 2021, while predicting their future development to 2050.

**Methods:**

Using data from the Global Burden of Disease 2021 study, we analysed prevalence, disability-adjusted life years, age-standardized rates (ASRs), and average annual per cent changes across sex, age, region, country, and social-development index (SDI). Temporal trend analysis was conducted using Joinpoint regression, while decomposition analysis was applied to pinpoint the factors driving changes in disease burden. A Bayesian age–period–cohort model generated projections.

**Results:**

Globally, despite the rise in oral disease cases, the ASRs have decreased. Between 1990 and 2021, the age-standardized prevalence rates (ASPR) fell from 65.17 thousand (95% confidence interval [CI]: 58.80-70.65) to 62.72 thousand (95% CI: 57.32-67.88) per 100,000 population (average annual percentage changes: −0.12, 95% CI: −0.36 to −0.12). Dental caries, other oral diseases, and edentulism decreased in ASRs, while periodontal disease increased. Women experienced a higher burden than men, though the decline was faster among men. ASPR declined in all age groups, and the most significant reduction was seen in those aged 75 to 79 years. In the 21 Global Burden of Disease regions, oral disease ASPR showed a negative correlation with SDI, with the most significant reductions occurring in high-SDI regions. Decomposition analysis showed population growth was the main driver. The projections indicate a significant increase in case numbers, whereas ASRs are expected to rise only modestly.

**Conclusions:**

These findings underscore the growing challenge of managing oral diseases among older adults and highlight significant disparities by sex, age, SDI, and region, emphasizing the urgent need for targeted prevention and resource allocation in low- and middle-SDI settings.

## Introduction

Oral disorders are counted among the world’s most widespread health issues, impacting more than 3.5 billion individuals across the globe. These conditions represent a significant public health challenge, exerting considerable economic and health pressures.^1^^,^^2^ Significant disparities exist in oral disease burden across nations at different economic development levels, with particularly high prevalence persisting in low- and middle-income nations.^2^^,^^3^ By 2021, major oral diseases worldwide were estimated to have an age-standardized prevalence of about 45,900 cases (95% uncertainty interval, 42,300-49,800) per 100,000 people.^4^ The global burden of oral diseases has shown minimal improvement during the last 30 years and continues to impose a substantial health challenge.^4^ In 2019, oral diseases imposed a global economic burden estimated at US$710 billion.^5^ In low-income countries, per capita spending on dental care was approximately 500 times lower than that in high-income nations.^5^

Global populations of older adults have been rapidly expanding in recent decades, with projections indicating they will reach two billion by 2050.^6^ Accelerated population ageing has heightened concerns about elderly oral health, generating substantial societal and economic challenges. Global Burden of Diseases (GBD) 2021 data indicate that more than 280 million people aged 70 and older are affected by oral conditions. These conditions are consistently listed among the top contributors to global disability-adjusted life years (DALYs).^7^ In ageing populations, maintaining oral health and preventing oral diseases may substantially enhance systemic health and life quality.^8^ However, comprehensive epidemiological understanding of global oral disease burden among adults aged ≥65 years remains limited. Most existing studies focus on broad age groups, failing to clearly present the unique health patterns and risks of this age group. Consequently, a detailed assessment of the global burden of oral conditions among adults aged ≥65 years is required to develop effective interventions and allocate healthcare resources rationally.

This study collected global oral disease data from 1990 to 2021 through the GBD 2021, conducting the first global systematic assessment of oral disease burden in older adults. Specifically, we analysed global trends in prevalence and DALYs of oral conditions among older adults (≥65 years). Data were stratified by age group, sex, and social-development index (SDI), with multinational trends presented across global, regional, and country levels. We also projected disease burden trajectories through 2050 to anticipate future epidemiological patterns.

## Materials and methods

### Overview and data source

We analysed data on oral diseases among the older population from the GBD 2021. The GBD study estimated Years of Life Lost, Years Lived with Disability, DALYs, and Healthy Life Expectancy for 371 diseases and injuries across 204 countries and territories. These countries and territories were grouped into 21 regions and 7 super-regions.^9^ Data were obtained from diverse sources, such as vital registries, verbal autopsy interviews, national censuses, survey programs, clinical registries, disease surveillance systems, and supplementary sources.^9^ Main oral conditions represent the composite disease burden from untreated tooth decay (deciduous/permanent), severe periodontal disease, edentulism, and related oral health disorders.^4^ Due to data scarcity and inconsistencies, especially in less developed areas, statistical models were used to estimate the disease burden by incorporating relevant covariates. Data failing to align with the age or sex categories specified by the GBD was adjusted, derived from prior DisMod-MR 2.1 age–sex estimates.^10^

### Statistical analysis

All age-standardized metrics, including prevalence, incidence, and DALY rates expressed per 100,000 population, along with their corresponding 95% confidence intervals (CIs), were computed using the GBD 2021 reference population as the standardization basis. The age-standardized rates (ASRs) per 100,000 people were computed using the formula outlined below: $ASR=\frac{\sum_{i=1}^{A}\alpha_{i\;}\omega_{i}}{\sum_{i=1}^{A}\omega_{i}}$, where $\alpha_{i}$ is the age specific rate and $\omega_{i}$ is the weight in the same age subgroup of the chosen reference standard population (in which *i* denotes the ith age class) and A is the upper age limit.^11^ Average annual percentage changes (AAPCs) and trend inflection points in ASRs from 1990 to 2021 were calculated using Joinpoint regression. AAPCs represent weighted averages of annual percentage changes (APCs).^12^ Spearman correlation analysis assessed ASRs associations with SDI across regions and nations, and significance was defined as *P* < .05. Beyond historical trends, we projected the global oral disease burden through 2050 to inform health policy and resource allocation. Projections were generated using the Bayesian age–period–cohort model with integrated nested Laplace approximation, which offers higher accuracy and coverage than traditional APC models.^13^ The Bayesian age–period–cohort model provides both age-specific and age-standardized forecasts, incorporating Poisson noise when prediction is the primary focus.

### Software and visualization

In this study, Joinpoint software (version 4.9.1) and RStudio (version 4.3.3) were applied for all statistical analyses. Visualizations were generated by RStudio’s ggplot2 package and finalized with Adobe Illustrator (2020) and Adobe Photoshop (CS). The map lines are used to define the study regions and may not reflect officially recognized national borders. Sex denotes the biological characteristics linked to physiological and physical traits, such as internal and external anatomy, chromosomes, hormone levels. Further methodological details can be found in the Appendix.

## Results

### Global trends

**Fig. 1:**
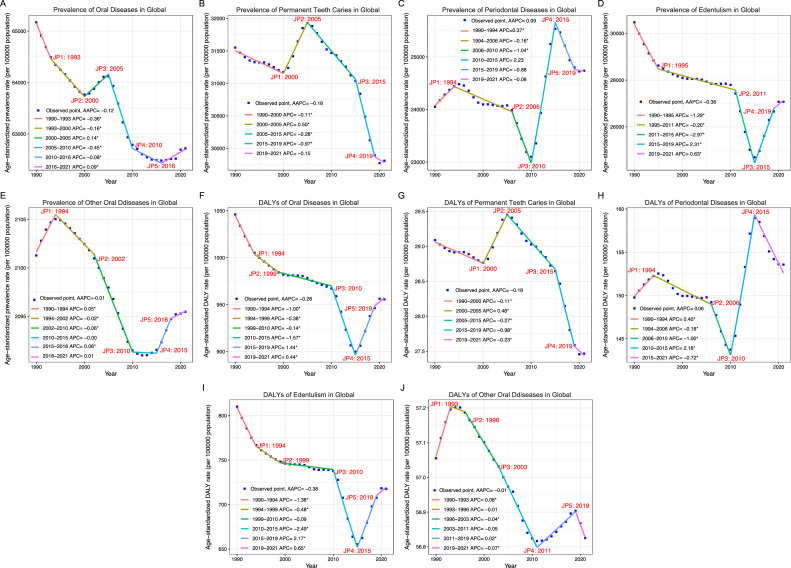
Joinpoint regression analysis of global prevalence for oral diseases (A), permanent teeth caries (B), periodontal diseases (C), edentulism (D), other oral diseases (E), and DALYs for oral diseases (F), permanent teeth caries (G), periodontal diseases (H), edentulism (I), and other oral diseases (J), in older adults aged 65 and over from 1990 to 2021. AAPC, average annual percentage change; APC, annual percentage change; DALYs, disability-adjusted life-years; JP, Joinpoint; *P < .05.

From 1990 to 2021, the global age-standardized prevalence rates (ASPR) for oral conditions in older adults decreased significantly from 65.17 thousand (95% CI: 58.80-70.65) to 62.72 thousand (95% CI: 57.32-67.88) per 100,000 population (AAPC: −0.12, 95% CI: −0.36 to −0.12) (Table 1). Five significant trend changes were detected in 1993, 2000, 2005, 2010, and 2016 using Joinpoint regression analysis, with notable declines from 1990 to 2000 and 2005 to 2016, and a slight rise from 2000 to 2005 (Figure 1A). From 1990 to 2021, the age-standardized DALY rates (ASDR) also declined significantly (AAPC: −0.28, 95% CI: −0.3 to −0.26, Table 2). Dental caries remained the most prevalent oral disease, accounting for 49.32% of cases in 1990 and 48% in 2021 (Table 1). Although cases of dental caries, edentulism, and other oral conditions increased, their ASRs stayed stable or declined, with edentulism showing the largest drop. Periodontal disease was the only one among the four categories of diseases that had increased during the period 1990 to 2021. A similar pattern was observed in the ASDR (Table 2).

**Table 1:** The prevalence of oral diseases and their trends from 1990 to 2021 at global and regional levels.

	Prevalence				
	Cases in 1990 (million)	Cases in 2021 (million)	ASR per 100 000 in 1990 (000s)	ASR per 100 000 in 2021 (000s)	AAPC 1990-2021
Global	211.9 (191.2-229.6)	482.6 (441.1-522.5)	65.17 (58.80-70.65)	62.72 (57.32-67.88)	−0.12 (−0.13 to −0.12)
Sex					
Male	90.1 (81.1-97.9)	215.6 (196.5-234.0)	64.53 (58.1-70.11)	61.82 (56.34-67.06)	−0.14 (−0.14 to −0.13)
Female	121.8 (110.0-131.8)	267.1 (244.4-288.8)	65.65 (59.29-71.09)	63.46 (58.09-68.61)	−0.11 (−0.11 to −0.10)
Age group (y)					
65-69	78.6 (70.8-85.5)	169.3 (154.8-184.3)	63.62 (57.31-69.15)	61.36 (56.13-66.81)	−0.12 (−0.12 to −0.11)
70-74	55.2 (49.5-60.0)	129.5 (117.6-140.7)	65.23 (58.51-70.86)	62.89 (57.14-68.36)	−0.12 (−0.12 to −0.11)
75-79	40.9 (37.2-43.9)	84.0 (77.4-89.8)	66.37 (60.50-71.29)	63.66 (58.65-68.08)	−0.14 (−0.14 to −0.13)
80-84	23.7 (21.5-25.6)	56.3 (51.7-60.3)	67.04 (60.70-72.39)	64.34 (58.98-68.81)	−0.13 (−0.14 to −0.12)
85-89	10.1 (9.1-10.9)	29.2 (26.6-31.6)	66.56 (59.97-72.22)	63.78 (58.19-69.09)	−0.13 (−0.14 to −0.13)
90-94	2.8 (2.5-3.1)	11.2 (10.1-12.2)	64.84 (58.23-71.28)	62.46 (56.47-68.42)	−0.12 (−0.13 to −0.11)
95 plus	0.6 (0.6-0.7)	3.3 (2.9-3.6)	62.26 (54.95-69.82)	60.17 (53.50-66.73)	−0.11 (−0.11 to −0.1)
SDI Level					
Low SDI	10.9 (9.9-11.8)	23.5 (21.3-25.5)	66.78 (60.39-72.25)	63.27 (57.44-68.56)	−0.18 (−0.19 to −0.17)
Low-middle SDI	30.5 (27.4-33.0)	76.9 (69.9-83.4)	68.74 (61.86-74.51)	67.28 (61.22-72.87)	−0.07 (−0.09 to −0.06)
Middle SDI	50.2 (45.1-54.6)	147.3 (134.8-159.3)	65.18 (58.64-70.83)	64.28 (58.85-69.48)	-0.04 (−0.05 to −0.03)
High-middle SDI	54.4 (48.9-59.2)	115.3 (104.9-125.5)	65.73 (59.01-71.57)	62.85 (57.15-68.38)	−0.14 (−0.16 to −0.13)
High SDI	65.6 (59.4-71.0)	119.1 (107.8-130.2)	62.99 (57.00-68.27)	58.30 (52.81-63.71)	−0.24 (−0.25 to −0.23)
GBD region					
Andean Latin America	1.3 (1.2-1.4)	4.1 (3.8-4.4)	83.85 (77.62-88.93)	81.98 (75.20-87.58)	−0.07 (−0.08 to −0.06)
Australasia	1.6 (1.5-1.7)	3.3 (2.9-3.7)	72.13 (67.62-76.46)	63.81 (55.57-71.76)	−0.40 (−0.42 to −0.38)
Caribbean	1.6 (1.4-1.7)	3.2 (2.8-3.5)	69.67 (62.51-76.25)	66.39 (59.26-73.22)	−0.15 (−0.16 to −0.15)
Central Asia	2.4 (2.2-2.7)	4.0 (3.4-4.5)	69.31 (61.88-76.05)	65.63 (56.89-74.06)	−0.18 (−0.18 to −0.17)
Central Europe	9.2 (8.2-10)	15 (13.2-16.8)	70.02 (62.87-76.60)	67.16 (59.24-75.05)	−0.13 (−0.15 to −0.12)
Central Latin America	4.4 (4.0-4.8)	14.4 (13.2-15.7)	69.07 (62.33-75.13)	68.01 (62.21-74.14)	−0.06 (−0.08 to −0.05)
Central Sub-Saharan Africa	1.0 (0.9-1.1)	2.1 (1.8-2.4)	63.74 (55.98-70.75)	59.70 (51.04-68.15)	−0.21 (−0.22 to −0.2)
East Asia	38.6 (34.1-42.4)	115.6 (104.7-125.9)	58.08 (51.42-63.90)	57.50 (52.10-62.65)	−0.03 (−0.05 to −0.02)
Eastern Europe	15.7 (14.1-17.2)	22.8 (20.6-25.0)	67.37 (60.53-73.75)	68.03 (61.57-74.60)	0.03 (0.02-0.03)
Eastern Sub-Saharan Africa	3.5 (3.1-3.8)	7.0 (6.3-7.7)	64.31 (57.42-70.24)	59.04 (52.70-65.09)	−0.28 (−0.29 to −0.28)
High-income Asia Pacific	8.5 (7.3-9.6)	21.4 (18.4-24.4)	49.41 (42.57-55.66)	45.63 (39.07-52.06)	−0.2 (−0.26 to −0.14)
High-income North America	20.6 (18.5-22.7)	36.1 (32.4-39.8)	60.00 (53.83-65.98)	56.23 (50.46-62.03)	−0.2 (−0.22 to −0.18)
North Africa and Middle East	8.9 (8.1-9.6)	24.1 (21.8-26.2)	72.07 (65.09-77.90)	70.52 (63.88-76.59)	−0.07 (−0.07 to −0.07)
Oceania	0.1 (0.1-0.2)	0.3 (0.3-0.3)	71.27 (63.65-77.46)	63.60 (54.19-72.00)	−0.37 (−0.38 to −0.36)
South Asia	28.4 (25.6-30.7)	82.6 (75.4-89.4)	70.41 (63.52-76.04)	69.04 (63.03-74.68)	−0.08 (−0.11 to −0.06)
Southeast Asia	12.5 (11.1-13.8)	33.4 (30.1-36.8)	67.76 (60.19-74.58)	65.44 (58.93-71.95)	−0.11 (−0.12 to −0.11)
Southern Latin America	3.1 (2.8-3.4)	6.1 (5.6-6.5)	75.78 (68.37-81.78)	75.34 (68.93-80.62)	−0.02 (−0.03 to −0.02)
Southern Sub-Saharan Africa	1.2 (1.0-1.3)	2.4 (2.1-2.7)	56.51 (49.34-63.34)	52.98 (46.04-60.09)	−0.2 (−0.21 to −0.18)
Tropical Latin America	5.5 (5.1-6.0)	17.5 (16.3-18.7)	78.36 (71.77-84.09)	78.89 (73.28-84.17)	0.01 (0.01-0.02)
Western Europe	39.5 (35.7-42.6)	60.3 (54.4-65.6)	70.80 (63.86-76.40)	67.14 (60.64-73.02)	−0.18 (−0.19 to −0.16)
Western Sub-Saharan Africa	4.0 (3.6-4.4)	7.0 (6.2-7.8)	59.48 (52.99-65.26)	51.01 (45.44-56.57)	−0.49 (−0.51 to −0.48)
Disease type					
Caries of permanent teeth	104.5 (75.9-132.2)	231.6 (172.5-290.1)	31.55 (22.79-40.13)	29.81 (22.18-37.41)	−0.18 (−0.19 to −0.17)
Periodontal diseases	79.6 (59.0-101.2)	192.0 (153.3-230.8)	24.05 (17.74-30.62)	24.74 (19.72-29.78)	0.09 (0.06-0.11)
Edentulism	95.8 (72.5-121.7)	204.4 (164.3-250.6)	30.47 (23.11-38.55)	27.07 (21.78-33.12)	−0.36 (−0.40 to −0.33)
Other oral disorders	7.0 (6.3-7.8)	16.4 (14.5-18.1)	2.10 (1.87-2.33)	2.10 (1.86-2.32)	−0.01 (−0.01 to −0.01)

Data in parentheses represent 95% uncertainty intervals for ASRs of the specified subage groups and for the case numbers of all groups, and represent 95% confidence intervals for ASRs of all the remaining groups and for AAPCs of all groups.

AAPC, average annual percentage change; ASR, age-standardised rate; GBD, Global Burden of Diseases, Injuries, and Risk Factors Study; SDI, social-development index.

**Table 2:** The DALYs of oral diseases and their trends from 1990 to 2021 at the global and regional levels.

	DALYs				
	Cases in 1990 (000s)	Cases in 2021 (000s)	ASR in 1990	ASR in 2021	AAPC 1990-2021
Global	3344.1 (2102.3-4977)	7280.9 (4586.7-10,568.1)	1046.01 (659.37-1550.96)	955.58 (602.69-1384.58)	−0.28 (−0.3 to −0.26)
Sex					
Male	1327.2 (826.1-2000.4)	3061.4 (1911.6-4491.2)	980.3 (613.15-1467.64)	896.55 (561.08-1310.63)	−0.28 (−0.3 to −0.25)
Female	2016.9 (1277.1-2979.1)	4219.4 (2674.7-6074)	1095.07 (693.93-1614.55)	1004.59 (636.97-1445.4)	−0.26 (−0.29 to −0.25)
Age group (y)					
65-69	1067.9 (637.9-1623.2)	2169.0 (1324.2-3222.6)	863.93 (516.05-1313.13)	786.33 (480.05-1168.27)	−0.3 (−0.32 to −0.28)
70-74	864.7 (544.8-1304.6)	1907.1 (1213.5-2820.5)	1021.36 (643.45-1540.99)	926.51 (589.53-1370.25)	−0.3 (−0.33 to −0.28)
75-79	713.4 (469.8-1053.6)	1374.7 (874.5-1946.3)	1158.88 (763.21-1711.64)	1042.34 (663.05-1475.79)	−0.32 (−0.35 to −0.3)
80-84	439.6 (283.5-627.2)	1007.4 (643.5-1417)	1242.68 (801.3-1772.99)	1150.19 (734.7-1617.88)	−0.22 (−0.24 to −0.2)
85-89	192.1 (124.0-274.1)	544.9 (352.1-769.3)	1271.27 (820.87-1813.79)	1191.69 (769.99-1682.49)	−0.2 (−0.22 to −0.18)
90-94	53.9 (34.5-76.4)	213.8 (137.8-302.2)	1258.01 (805.22-1783.99)	1195.06 (770.22-1689.05)	−0.16 (−0.17 to −0.14)
95 plus	12.6 (7.8-17.8)	64.0 (41.3-90.3)	1234.22 (769.98-1752.33)	1174.22 (757.44-1656.07)	−0.14 (−0.16 to −0.12)
SDI level					
Low SDI	117.5 (71.3-181.7)	245.2 (149-366.9)	754.12 (461.24-1152.66)	687.56 (420.1-1022.47)	−0.33 (−0.39 to −0.29)
Low-middle SDI	394.6 (245.4-594.3)	953.5 (589.3-1372.4)	924.51 (578.31-1381.58)	860.12 (534.1-1233.08)	−0.25 (−0.3 to −0.19)
Middle SDI	809.2 (506-1201.6)	2296 (1447.3-3312.7)	1083.88 (682.27-1598.29)	1025.31 (648.28-1473.43)	−0.2 (−0.26 to −0.16)
High-middle SDI	905.9 (568.3-1343.5)	1848.9 (1172.5-2680.4)	1110.56 (698.36-1641.84)	1016.82 (645.47-1471.73)	−0.26 (−0.29 to −0.24)
High SDI	1112.3 (706.9-1645.9)	1929.6 (1221-2825.4)	1067.47 (677.59-1579.61)	935.13 (590.72-1371.3)	−0.43 (−0.47 to −0.39)
GBD region					
Andean Latin America	28.8 (19-40.9)	85.5 (56.8-120.8)	1808.12 (1195.9-2567.03)	1707.8 (1135.39-2413.4)	−0.18 (−0.2 to −0.17)
Australasia	33.3 (22.4-46.5)	59.6 (37.8-88.4)	1492.33 (1005.56-2079.71)	1143.63 (722.94-1695.76)	−0.79 (−0.88 to −0.69)
Caribbean	27.3 (17.2-40.5)	54.1 (33.9-80)	1218.11 (768.12-1800.68)	1134.24 (711.75-1679.31)	−0.24 (−0.25 to −0.22)
Central Asia	40.5 (25.5-60.4)	62.4 (39.1-93)	1163.21 (732.03-1731.67)	1061.1 (669.05-1575.18)	−0.29 (−0.31 to −0.28)
Central Europe	170.1 (108.9-247.4)	266.8 (169.1-392.9)	1316.28 (845.37-1909.37)	1196.07 (758.72-1760.58)	−0.29 (−0.34 to −0.24)
Central Latin America	74.9 (47.4-110.7)	242 (152.5-339.2)	1182.92 (750.42-1744.07)	1147.22 (723.71-1606.01)	−0.12 (−0.15 to −0.09)
Central Sub-Saharan Africa	10.4 (6.2-16.1)	22.1 (13.4-33.7)	703.88 (425.32-1081.72)	654.18 (399.85-992.55)	−0.24 (−0.25 to −0.23)
East Asia	635.8 (389.9-963.2)	1848.5 (1157-2749.2)	1000.46 (617.74-1500.48)	948.44 (595.73-1402.57)	−0.17 (−0.21 to −0.13)
Eastern Europe	278.4 (176.3-410.2)	415.4 (259.4-577.3)	1199.85 (759.68-1765.4)	1250.79 (781.48-1737.82)	0.13 (0.12-0.13)
Eastern Sub-Saharan Africa	30.4 (17.7-49.1)	60.4 (35.8-94.1)	581.28 (341.46-927.22)	528 (314.01-817.52)	−0.31 (−0.31 to −0.3)
High-income Asia Pacific	153.1 (94.4-232.1)	364.9 (228.6-536.4)	895.2 (553.56-1353.3)	746.63 (465.44-1102.97)	−0.49 (−0.66 to −0.32)
High-income North America	369.9 (233.3-552.3)	579.5 (366.7-853.7)	1074.71 (676.89-1604.55)	902.62 (570.85-1329.1)	−0.55 (−0.6 to −0.51)
North Africa and Middle East	147.7 (93.5-217.9)	387.8 (246.1-567.1)	1216.01 (774.57-1784.7)	1151.77 (734.36-1676.32)	−0.18 (−0.19 to −0.17)
Oceania	1.9 (1.2-2.9)	4.1 (2.5-6)	1039.44 (645.93-1557.5)	891.98 (561.98-1305.88)	−0.49 (−0.5 to −0.48)
South Asia	324.6 (199.2-495.6)	890.4 (539.2-1300)	845.71 (522.12-1276.82)	773.68 (471.51-1124.07)	−0.31 (−0.41 to −0.23)
Southeast Asia	180.5 (112.3-270.3)	459.9 (286.4-666.7)	1005.21 (628.63-1497.43)	924.25 (577.82-1334.48)	−0.27 (−0.28 to −0.26)
Southern Latin America	47.8 (29.8-70.7)	85.5 (53.6-127.6)	1170.59 (730.04-1727.27)	1052.04 (658.69-1569.89)	−0.35 (−0.36 to −0.34)
Southern Sub-Saharan Africa	17.4 (10.8-26)	33.3 (20.9-49.4)	840.58 (522.84-1252.24)	765.81 (481.36-1133.79)	−0.27 (−0.31 to −0.23)
Tropical Latin America	117.9 (77.9-167.4)	372.7 (240.8-509.9)	1684.24 (1118.14-2383.32)	1684.19 (1089.1-2302.33)	0 (−0.02 to 0.01)
Western Europe	611.1 (386.5-896.9)	911.5 (579.1-1340.8)	1089.84 (687.39-1602.08)	997.33 (631.06-1471.26)	−0.26 (−0.32 to −0.21)
Western Sub-Saharan Africa	42.2 (25-67.1)	74.4 (44.8-111.8)	644.16 (383.61-1014.85)	558.06 (337.57-833.82)	−0.47 (−0.5 to −0.44)
Disease type					
Caries of permanent teeth	96.6 (43.2-182.9)	213.7 (95.5-406.5)	29.09 (12.99-55.14)	27.47 (12.28-52.23)	−0.18 (−0.2 to −0.18)
Periodontal diseases	497.4 (189.4-1063.8)	1193.5 (457.8-2452.1)	149.78 (57.12-319.9)	153.57 (58.92-315.87)	0.06 (0.03-0.09)
Edentulism	2558.7 (1620-3783.4)	5429.5 (3500.6-7755.8)	810.09 (514.28-1193.77)	717.72 (463.11-1024.34)	−0.38 (−0.41 to −0.35)
Other oral disorders	191.4 (118.3-287.5)	444.1 (274-666.3)	57.06 (35.25-85.58)	56.83 (35.05-85.18)	−0.01 (−0.01 to −0.01)

Data in parentheses represent 95% uncertainty intervals for ASRs of the specified subage groups and for the case numbers of all groups, and represent 95% confidence intervals for ASRs of all the remaining groups and for AAPCs of all groups.

AAPC, average annual percentage change; ASR, age-standardised rate; DALYs, disability-adjusted life years; GBD, Global Burden of Diseases, Injuries, and Risk Factors Study; SDI, social-development index.

### Global trends by sex

The ASPR of oral diseases among older adults declined in both sexes between 1990 and 2021, with a steeper decline observed in males (AAPC: −0.14, 95% CI: −0.14 to −0.13) than in females (AAPC: −0.11, 95% CI: −0.11 to −0.10) (Table 1). A greater overall decline in ASDR was observed, with males showing an AAPC of −0.28 (95% CI: −0.30 to −0.25) and females −0.26 (95% CI: −0.29 to −0.25) (Table 2). Throughout the period, females showed persistently greater ASPRs and ASDRs (Appendix Figure S1A,F). The steepest ASPR decline occurred from 2005 to 2010 for males and 2005 to 2009 for females (Appendix Figure S1A). Between 2010 and 2015, ASDR declined markedly in both sexes, with APCs of −1.69 for males and −1.46 for females (Appendix Figure S1F).

Regarding specific diseases, from 1990 to 2021, males consistently showed higher ASPRs of periodontitis than females globally (Appendix Figure S1C). In contrast, females bore a heavier burden of dental caries, edentulism, and other oral diseases (Appendix Figure S1B,D,E). Notably, the ASPR of periodontitis in females increased more rapidly (AAPC: 0.10, 95% CI: 0.07-0.12) than in males (AAPC: 0.03, 95% CI: 0.01-0.16) (Appendix Table S3). Meanwhile, the decrease in ASPR of dental caries and edentulism was slower in females than in males (Appendix Figure S1B,D, and Appendix Tables S1 and S2). In terms of other oral diseases, the ASPR increased in females but decreased in males (Appendix Figure S1E and Appendix Table S4). Global sex-specific trends are shown in Appendix Figure S1.

### Global trends by age group

The ASPR of oral diseases declined across all age groups between 1990 and 2021, with the largest decrease in the 75 to 79 age group (AAPC: −0.14, 95% CI: −0.14 to −0.13, Table 1). A similar trend was observed for the ASDR (Table 2). In 2021, both ASPR and ASDR peaked in the 80 to 84 age group. Below this age, the burden rose with age, while above it, the burden gradually declined (Table 1, Table 2).

By disease type, the steepest drop in dental caries ASPR occurred in the 90 to 94 age group, the largest drop in edentulism in the 65 to 69 group, and the greatest rise in periodontal disease in the 80 to 84 group, while changes in other oral diseases were minor. Appendix Figure S2 shows the global age-specific trends. The Joinpoint regression results and AAPC are shown in Appendix Table S5 and Appendix Figure S2.

### Global trends by SDI

Between 1990 and 2021, the most pronounced decline in ASPR among older adults was observed in the high-SDI quintile (AAPC: −0.24, 95% CI: −0.25 to −0.23), with downward trends also seen in the other four quintiles (Table 1). Joinpoint regression analysis indicated that the high-SDI quintile had the lowest ASPR, whereas the low-SDI quintile had the lowest ASDR (Appendix Figure S3). Detailed results for total oral diseases and specific conditions across SDI quintiles are shown in Appendix Figure S3.

**Fig. 2:**
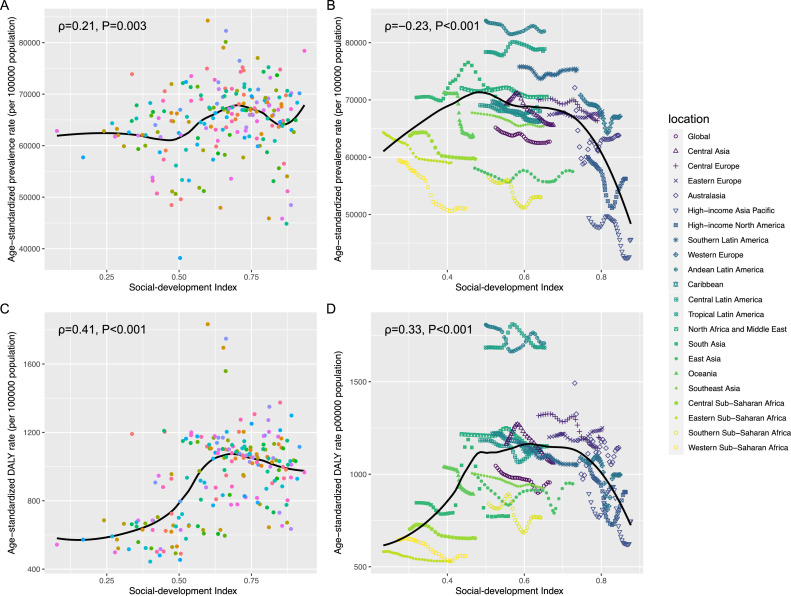
Spearman correlation analysis between the prevalence (A), and DALYs (C) of oral diseases and the SDI in 2021 at the national level, and between the prevalence (B), and DALYs (D) and the SDI from 1990 to 2021 at the regional level. DALYs, disability-adjusted life-years; SDI, social-development index.

In 2021, the highest ASPR was observed in low-middle SDI quintiles and the lowest in high-SDI quintiles, whereas ASDR peaked in middle-SDI quintiles and reached its minimum in low-SDI quintiles (Table 1, Table 2). Across the 204 countries, both ASPR and ASDR were positively correlated with SDI (National level, *P* = .003; Regional level, *P* < .001; Figure 2A,C). Among the 21 GBD regions, ASPR was negatively correlated with SDI, while ASDR showed a positive correlation with SDI (both *P* < .001; Figure 2B,D).

### Regional trends

From 1990 to 2021, most regions experienced a decline in the ASPR of oral diseases, with the steepest decreases occurring in Western Sub-Saharan Africa (AAPC: −0.49; 95% CI: −0.51 to −0.48) and in Australasia (AAPC: −0.40; 95% CI: −0.42 to −0.38, Table 1). Similarly, ASDR decreased across the majority of regions, with Australasia and High-income North America experiencing the most pronounced declines (Table 2). In 2021, Andean Latin America exhibited the greatest ASPR and ASDR values (Table 1, Table 2).

The ASPR and ASDR of dental caries, edentulism, and other oral disorders generally showed declining trends across the 21 GBD regions from 1990 to 2021. However, periodontal disease increased in several regions in both measures. High-income Asia Pacific had the largest drop in dental caries ASR (Appendix Table S1), while Australasia showed the biggest decline in edentulism (Appendix Table S2). Oceania showed the greatest decrease in ASR for periodontal disease (Appendix Table S3). Central Asia showed the greatest reduction in ASPR for other oral disorders (Appendix Table S4). Joinpoint regression outcomes for regional ASPR are presented in Appendix Table S6.

### National trends

**Fig. 3:**
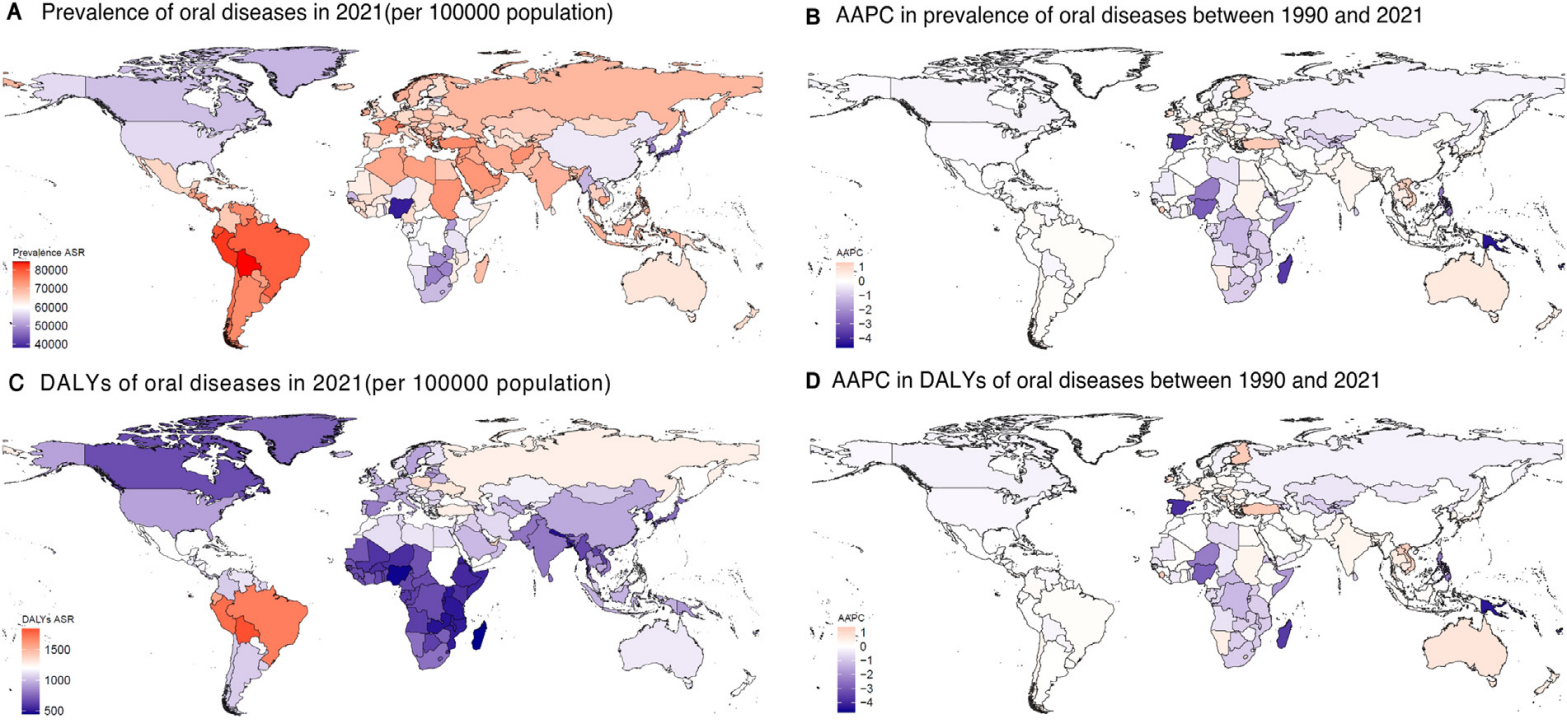
Global map of prevalence (A), and DALYs (C) for oral diseases in 2021 and AAPCs in prevalence (B), and DALYs (D) from 1990 to 2021. AAPC, average annual percentage change; ASDR, age-standardized DALY rate; ASPR, age-standardized prevalence rate; DALYs, disability-adjusted life-years.

At the national level, Nigeria recorded the fastest reduction in ASPR of oral diseases between 1990 and 2021 (AAPC −1.12, 95% CI −1.16 to −1.09), whereas Spain exhibited the greatest decline in ASDR (AAPC −1.20, 95% CI −1.23 to −1.18). Bolivia recorded the greatest levels of ASPR and ASDR in 2021 (Figure 3 and Appendix Table S7). Appendix Table S8 presents the Joinpoint regression findings for ASPR at the national level.

From 1990 to 2021, Morocco achieved the most pronounced reduction in dental caries ASPR, and Kiribati in periodontal disease ASPR (Appendix Table S9). Switzerland exhibited the highest ASPR for dental caries in 2021, and Gambia for periodontal disease. Country-specific AAPCs and ASRs for periodontal disease and dental caries are provided in Appendix Tables S9 and S10.

### Decomposition analysis

Globally, DALY cases rose sharply over the past three decades, primarily due to population growth, which explained 111.36% of the rise, while ageing contributed 1.24%. In contrast, epidemiological changes reduced DALYs by 12.6% (Appendix Figure S4 and Appendix Table S11). The middle-SDI quintile saw the largest rise, with women affected more than men. Population growth was the dominant driver across all SDI levels and both sexes (Appendix Figure S4 and Appendix Table S11).

### Predictive analysis

**Fig. 4:**
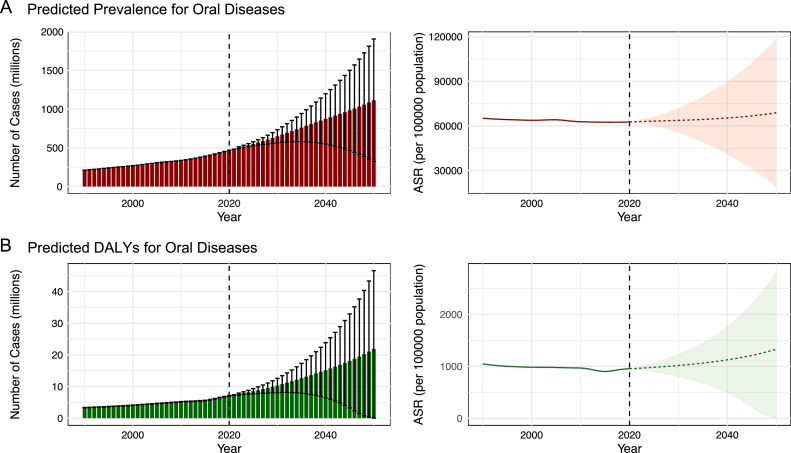
The predicted case number and ASR of prevalence (A), and DALYs (B) for oral diseases in older adults aged 65 and over in 2050 globally. ASR, age-standardized rate; DALYs, disability-adjusted life-years.

Figure 4 illustrates the projected trends in case numbers and ASRs for oral diseases among the global elderly population through 2050. The projections suggest a substantial increase in the number of cases, while ASPR and ASDR are expected to rise only slightly. The number of prevalence cases is projected to increase from 468.84 (468.64-469.04) million in 2020 to 1114.33 (322.84-1905.82) million in 2050, while the number of DALYs is expected to increase from 7081.67 (6979.02-7184.33) thousand in 2020 to 21,899.57 (36.7-46,592.32) thousand in 2050. Appendix Table S12 presents detailed information regarding case numbers and ASRs of oral disease up to 2050. The increase in oral disease cases observed over the past three decades is likely attributable mainly to population growth and ageing, which may also account for the predicted rise by 2050. These projections highlight the heavy burden and emerging challenges in the control and management of oral diseases, which will place growing demands on healthcare systems. Therefore, it is crucial for countries worldwide to develop effective public health policies to optimally prepare for such demographic transitions.

## Discussion

This research analysed worldwide trends in oral conditions among adults aged 65 and above between 1990 and 2021. Over the past three decades, the overall cases increased, but the ASRs have decreased. The ASRs of permanent dental caries, edentulism, and other oral disorders decreased, while that of periodontal diseases rose slightly. Trends differed notably by sex, age, SDI, region, and country. Population growth served as a major factor contributing to the heightened burden among older adults. The projections indicate a significant increase in case numbers, whereas ASRs are expected to rise only modestly.

Despite fluctuations, the global prevalence of oral diseases in older adults showed a general downward tendency from 1990 to 2021. The ASPR dropped sharply from 1990 to 2000, partly due to the 1994 ‘International Year for Oral Health’ led by FDI and World Health Organization (WHO), which raised governmental and public oral health awareness.^14^ Subsequently, in 1999, the WHO systematically introduced the concept of ‘active ageing’ for the first time, encouraging a preventive approach to improving health among older adults.^15^ A slight rise from 2000 to 2005 may reflect population ageing, urbanization, higher sugar intake, and smoking.^16^^,^^17^ After International Association for Dental Research and WHO defined oral health goals and standards to be met by 2020,^18^ and integrated oral disease prevention into the prevention of noncommunicable chronic diseases,^19^ the ASPR fell again from 2005 to 2010. From 2010 to 2016, the decline in the ASPR slowed down, which may be attributed to the reduction in health expenditures following the 2008 global financial crisis,^20^^,^^21^ as well as the increasing global elderly population that expanded the baseline burden of oral diseases.^22^ A mild rebound from 2016 to 2021 was linked to COVID-19, which restricted dental services.^23^

Over the past 30 years, among the four major oral diseases, periodontal disease was the only one with an increasing burden (AAPC = 0.09, 95% CI: 0.06-0.11). The intensifying global ageing and the expanding base of the 65+ population are the primary reasons for the increasing burden of periodontal disease.^24^ Changes in population size and age distribution are difficult to influence through policy measures. In many developing countries, rising risk factors such as changes in dietary patterns, smoking, and diabetes have further exacerbated the burden of periodontal disease.^25^^,^^26^ Most low and middle-SDI countries have restricted access to oral health services and lack sufficient prevention and treatment.^27^^,^^28^ Comprehensive preventive measures, lifestyle modifications, and improvements in oral healthcare systems are essential strategies to address this issue. Numerous studies have demonstrated a significant association between periodontal disease and various systemic disorders, such as cardiovascular and cerebrovascular diseases, diabetes, respiratory disorders, obesity, and prostatitis.^29^, ^30^, ^31^ Oral health is closely linked to the overall health of older adults and constitutes an indispensable component of healthy ageing. Incorporating oral health issues into general medical care may contribute to improving oral health outcomes and enhancing the overall quality of healthcare in the elderly population.^8^

Research shows clear sex differences in oral disease burden, consistent with earlier studies.^32^ Globally, older women have a heavier overall burden and a slower decline in disease rates compared to men. Limited job opportunities often mean women earn less and have limited access to oral health education and care, increasing their risk.^33^ However, the burden of periodontal disease is greater among men. Earlier studies have reported that men are less likely to visit dentists and to utilize preventive care.^34^ Furthermore, they are more susceptible to periodontal disease, likely due to differences in immune function, hormonal factors, suboptimal oral hygiene practices, and higher tobacco use.^34^ The burden of edentulism is greater among women, mainly attributable to lower bone density and an elevated risk of osteoporosis, which renders women more prone to tooth loss.^35^ These sex disparities highlight the need for a more targeted and equitable allocation of resources.

This study showed that the highest ASPR of dental caries and periodontal disease was observed in the 65 to 69 age group from 1990 to 2021, while the highest rates of edentulism were observed in people aged ≥95 years. In older populations, especially those over 80, chronic oral diseases such as periodontitis and caries accumulate over time, eventually leading to tooth mobility, loss, compromised chewing, diminished quality of life, and increasing the risk of edentulism.^36^, ^37^, ^38^ In Japan, the ‘8020 Campaign’ encourages keeping at least 20 natural teeth by age 80, boosting public awareness and improving oral hygiene habits.^39^ Preventing or treating periodontal disease and dental caries plays a key role in preserving chewing, swallowing, and other oral functions.^40^ To achieve healthy ageing, dental care should be simple, prevention-focused, and focused on improving quality of life associated with oral health.^41^

At the regional level, Australasia experienced the greatest reductions in both ASPR and ASDR of oral diseases, likely due to prioritizing older adults in national oral health programs and adopting more equitable dental policies.^42^ Besides, the transition from predominantly extracting teeth to emphasizing restorative procedures has likely contributed to better tooth preservation,^43^ suggesting a link between better oral health and effective dental care models. Consistent evidence from Australia highlights the crucial role of widespread use of fluoride toothpaste and community water fluoridation in preventing dental caries.^44^^,^^45^ Additionally, Australia has promoted the integration of skilled dental professionals into aged care facilities to conduct risk evaluation and care planning for complex needs.^46^ Such initiatives can inform other regions in enhancing oral health.

Nigeria experienced the most pronounced decline in ASPR for oral diseases between 1990 and 2021 (AAPC = −1.12, 95% CI: −1.16 to −1.09), likely due to sustained promotion of oral health policies. After several setbacks, the country launched its national Oral Health Policy in 2012, integrating oral care into primary healthcare.^47^^,^^48^ Studies indicate that Oral Health Policy’s success relied on the interplay of context, process, and stakeholder engagement – key factors for low- and middle-income countries where policy processes are often opaque.^47^ In contrast, Bolivia recorded the highest ASPR and ASDR among older adults in 2021, with severe shortages of dental resources, low dental visit rates, limited preventive care, and poor service accessibility.^49^ WHO reports that up to 90% of older adults in Latin America, including Bolivia, have periodontal disease and edentulism.^50^ Despite similar SDI levels, Nigeria and Bolivia differ markedly in elderly oral health outcomes, underscoring the pivotal role of national oral health policies in low- and middle-SDI settings, where even modest gains can yield substantial benefits.^51^

Across the 21 GBD regions, SDI was inversely correlated with ASPR of oral diseases, highlighting the role of socioeconomic status in oral health outcomes, in agreement with previous findings.^2^^,^^52^ ASPR tends to rise and then fall as SDI increases. In 2021, low-middle SDI regions reported the greatest ASPR, whereas high SDI regions showed the lowest. Lower SDI areas often lack strong health surveillance systems, which may lead to underreporting of disease burden.^26^^,^^27^ Conversely, regions with high SDI typically possess better healthcare infrastructure, improved service accessibility, higher levels of public awareness, and improved living conditions, all contributing to lower oral disease rates.^53^ However, middle SDI regions may face rising disease burdens due to lifestyle changes, urbanization, and limited healthcare infrastructure.^54^ So, in developing countries, targeted interventions are essential. Public–private oral health partnerships, for example, may be particularly effective in these regions by improving service quality through private sector involvement, and could be scaled up across dental health systems.^55^

### Strengths and limitations

As far as we know, our study is the first to evaluate oral disease burden across older populations worldwide, regionally, and nationally, including projections through 2050. However, some limitations warrant consideration. First, the estimates largely depend on the availability and quality of 2021 GBD data, which may be incomplete in many low and middle-income countries, potentially leading to underestimation.^9^^,^^56^ Second, only four oral diseases were included in the analysis, while other conditions like oral cancer and congenital anomalies were excluded, narrowing its scope. Third, cross-country and temporal differences in diagnostic and detection approaches may impact the comparability of results. Fourth, projections to 2050 are derived from present data and could fail to consider policy adjustments or future global changes.

### Future directions

The global burden for oral diseases is projected to keep increasing through 2050. To support healthy ageing, oral health must be prioritized.^57^ Dental care should be streamlined, prevention-focused, and aimed at enhancing oral health–related quality of life.^41^ Strengthening interdisciplinary collaboration between dental medicine and geriatric is essential to improve oral health management in long-term care environments.^58^ Region-specific interventions, particularly the development of robust national oral health policies across low- and middle-SDI nations, could deliver substantial public health benefits.^51^ In addition, tailored care and prevention strategies must be adapted to the specific characteristics of older adults, including region, sex, age, and type of oral disease.

## Conclusion

This study analysed global trends in oral diseases among older individuals aged ≥65 years between 1990 and 2021, with projections to 2050. While the overall burden increased over the past three decades, ASRs declined. ASRs for permanent dental caries, edentulism, and other oral disorders fell, whereas those for periodontal disease rose slightly. Trends varied by sex, age, SDI, region, and country, with population growth serving as the primary contributor to the rising burden among older adults. These findings carry significant relevance for resource allocation and public health policy, especially for women and populations in low and middle-SDI countries.

## Author contributions

WL conceived the study. WQD and WL analysed the GBD data. WQD drafted the manuscript, and WL revised the manuscript. All authors have read and approved the final version of the manuscript.

## Conflict of interest

The authors report no conflicts of interest in this work.

## Appendix Supplementary materials

Image, application 1

Image, application 2

Image, application 3

Image, application 4

Image, application 5

## Footnotes

Supplementary material associated with this article can be found in the online version at doi:10.1016/j.identj.2025.109297.
