# Supplementary material for: Global, Regional, and National Burden of Oral Diseases in Older Adults Aged 65 Years And Over
Source: Int Dent J. 2025 Dec 9;76(1):109297. doi: 10.1016/j.identj.2025.109297 (PMC12753236; doi:10.1016/j.identj.2025.109297)
Supplement: Supplementary file 1 [file mmc1.doc]

**APPENDIX**

**Global Burden of Oral Diseases in Older Adults Aged 65 Years and Over**

Wei Lu, Wanqing Du, Xuejing Duan

**Methods**

***Case definitions for the five oral diseases in GBD 2021***

Dental caries (including those in deciduous and permanent teeth): Refers to teeth with cavities reaching the dentin, root surface lesions in the cementum that feel soft or leathery upon probing, the presence of temporary or permanent fillings, or missing teeth extracted due to caries.

Periodontal disease: In GBD, severe symptomatic periodontal disease is characterized by bad breath, unpleasant taste, and gums that occasionally bleed slightly, without interfering with normal daily activities.

Edentulism: Defined as the complete absence of permanent teeth in an individual, excluding the natural toothless stage in infancy.

Other oral disorders: Covers a broad spectrum of diseases and abnormalities affecting the teeth, tongue, and jaw, excluding those classified as caries, periodontal disease, or edentulism/severe tooth loss. This group includes disorders such as tooth development and eruption anomalies (K00–K01.1), other hard tissue and pulp diseases (K03–K04.99), dentofacial anomalies, tooth loss, and other related conditions (K07–K08), oral mucosal diseases and other oral cavity disorders (K08.8–K14.9), and temporomandibular joint or occlusion disorders (M26–M27.9), based on ICD-10 codes used in GBD 2021.

List of International Classification of Diseases (ICD) codes mapped to all oral diseases in GBD 2021

| CauseName | ICD10 | ICD9 |
| --- | --- | --- |
| Oral disorders | K00-K08.499, K08.8-K14.9, M26-M27.9 | 520-525.54, 525.8-526.61, 526.69-529.9, V07.31, V45.84, V49.82, V58.5, V72.2 |
| Caries of permanent teeth | K02-K02.9 | 521.0-521.09 |
| Periodontal diseases | K05-K06.9 | 523-523.9 |
| Edentulism | K08.0-K08.499 | 525.0-525.19, 525.4-525.54 |
| Other oral disorders | K00-K01.1, K03-K04.99, K07-K08, K08.8-K14.9, M26-M27.9 | 520-521, 521.1-522.9, 524-525, 525.2-525.3, 525.8-526.61, 526.69-529.9 |

***Data collection***

In this study using GBD 2021 data, we obtained repeated cross-sectional information on oral diseases from the publicly accessible Global Health Data Exchange (GHDx, <https://ghdx.healthdata.org/gbd-2021>). The GBD dataset covers 371 diseases and injuries across 204 countries and territories from 1990 to 2021, grouped into 21 regions and seven super-regions. Data are compiled from diverse sources, including epidemiological surveys, hospital records, vital registration systems, disease surveillance networks, and supplementary materials such as academic literature and policy documents[1].
We extracted case numbers and rates of prevalence, and disability-adjusted life years (DALYs) for oral diseases and their four subcategories, stratified by sex, seven age groups (65–69, 70–74, 75–79, 80–84, 85–89, 90–94, and 95 plus years), and by 204 countries and territories organized into the 21 GBD regions[1]. DALYs quantify disease burden as the sum of years of life lost (YLLs) and years lived with disability (YLDs). Because oral diseases are non-fatal, DALY estimates derive entirely from YLDs. Rates are expressed per 100,000 population, with calculations repeated 500 times to generate draw-level estimates. Final values are the mean of these 500 draws, and 95% uncertainty intervals (UIs) are defined by the 2.5th and 97.5th percentiles[1].

This study further examines the distribution of disease burden by SDI quintiles. The SDI is a composite indicator designed to capture the influence of social and economic conditions on health outcomes across different regions. It is calculated as the geometric mean of three core metrics: the total fertility rate among individuals under 25 years of age, the average years of education for people aged 15 and older, and lag-distributed income per capita[1]. In the GBD 2021 framework, SDI values are divided into five quintiles—low, low-middle, middle, high-middle, and high—with scores ranging from 0 to 100.

***The methodology details for oral diseases used in GBD 2021***

For the four oral diseases, data standardization and harmonization of reference definitions were achieved through crosswalks using the meta-regression–Bayesian, regularized, trimmed (MR-BRT) approach. Epidemiological models were then developed with DisMod-MR 2.1 (Disease Modeling Meta-Regression, version 2.1).
MR-BRT, created by the Institute for Health Metrics and Evaluation (IHME), integrates Bayesian priors, regularization, and trimming to manage heterogeneity and uncertainty in meta-regression analyses. This approach is effective for handling variability across studies, enabling the identification and exclusion of outliers, and adjusting for biases arising from differences in study designs and characteristics. Within the GBD framework, it is a critical tool for preprocessing data and assessing risk, forming the groundwork for subsequent epidemiological modeling and disease burden estimation.
DisMod-MR 2.1 is a Bayesian meta-regression tool that produces internally consistent estimates of prevalence, remission, and mortality, stratified by sex, location, year, and age group. For locations lacking raw epidemiological data, it estimates prevalence through a hierarchical cascade across the five levels of the GBD geographical structure, using data from higher levels as priors for lower levels. It also incorporates location-specific covariates to refine prevalence and incidence estimates where direct data are unavailable.

***Statistical analysis***

To eliminate the influence of age structure and improve comparability across regions, we measured the burden of oral diseases using age-standardized rates (ASRs), including age standardized prevalence rates (ASPRs), age standardized incidence rates (ASIRs), and age-standardized disability-adjusted life-year rates (ASDRs). For older adults, ASRs (per 100,000) were calculated using the global standard population from GBD 2021, applying the formula:
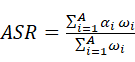
, where
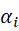
 is the age specific rate and
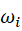
 is the weight in the same age subgroup of the chosen reference standard population (in which i denotes the
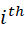
 age class) and A is the upper age limit[2]. Trends in ASRs were examined globally, regionally, and nationally using Joinpoint regression[3, 4]. The analysis involved: (1) segmented regression with a log-linear model, applying a grid search method to identify breakpoints by minimizing mean squared error (MSE); (2) selecting the optimal number of breakpoints (0–5) via Monte Carlo permutation tests; and (3) calculating the annual percent change (APC) and average annual percent change (AAPC) from the optimal model. APC was computed as: APC = 100 * (eβ ± 1.96 SE – 1), where β and SE represent the coefficient and standard error obtained from the linear regression model: ln(ASR) = α + β * (calendar year) + ε. AAPC was derived as the weighted average of APCs for each segment, weighted by segment length. APC values identified periods with the most marked changes in ASR trends between 1990 and 2021, while AAPC described the overall trend[4]. Positive AAPC indicates an upward trend, negative AAPC indicates a downward trend, and a 95% CI including zero suggests no statistically significant change.

To examine trends by age group, sex, and SDI, we applied Joinpoint regression as described above. Spearman correlation analysis was performed at regional and national levels in 2021 to assess the relationship between ASRs and SDI, with statistical significance set at P < 0.05.

Beyond historical trends, we projected the global oral disease burden through 2050 to inform health policy and resource allocation. Projections were generated using the Bayesian age–period–cohort (BAPC) model with integrated nested Laplace approximation (INLA), which offers higher accuracy and coverage than traditional APC models[5]. The BAPC model provides both age-specific and age-standardized forecasts, incorporating Poisson noise when prediction is the primary focus.

***Appendix References***

1. Global incidence, prevalence, years lived with disability (YLDs), disability-adjusted life-years (DALYs), and healthy life expectancy (HALE) for 371 diseases and injuries in 204 countries and territories and 811 subnational locations, 1990-2021: a systematic analysis for the Global Burden of Disease Study 2021. Lancet, 2024. 403(10440): p. 2133-2161.

2. Lv, B., et al., Epidemiological trends of subarachnoid hemorrhage at global, regional, and national level: a trend analysis study from 1990 to 2021. Mil Med Res, 2024. 11(1): p. 46.

3. Qiu, H., S. Cao, and R. Xu, Cancer incidence, mortality, and burden in China: a time-trend analysis and comparison with the United States and United Kingdom based on the global epidemiological data released in 2020. Cancer Commun (Lond), 2021. 41(10): p. 1037-1048.

4. Zhang, J., et al., Global, regional, and national burdens of HIV and other sexually transmitted infections in adolescents and young adults aged 10-24 years from 1990 to 2019: a trend analysis based on the Global Burden of Disease Study 2019. Lancet Child Adolesc Health, 2022. 6(11): p. 763-776.

5. Global pattern, trend, and cross-country inequality of early musculoskeletal disorders from 1990 to 2019, with projection from 2020 to 2050. Med, 2024. 5(8): p. 943-962.e6.

**Table S1. The prevalence and DALYs of permanent teeth caries and their trends from 1990 to 2021 at the global and regional levels**

|  | **Prevalence** | | | | **DALYs** | | | |
| --- | --- | --- | --- | --- | --- | --- | --- | --- |
|  | ASR in 1990 (000s) | ASR in 2021 (000s) | AAPC  (1990-2021) | | ASR in 1990 | ASR in 2021 | AAPC  (1990-2021) | |
| **Sex** | | | | | | | |  |
| Male | 31.84(22.88-40.47) | 29.57(22.01-37.13) | -0.23(-0.25 to -0.23) | | 29.42(13.16-55.67) | 27.37(12.24-51.95) | -0.23(-0.24 to -0.22) | |
| Famle | 31.39(22.79-39.99) | 30.04(22.32-37.67) | -0.14(-0.15 to -0.13) | | 28.89(12.86-54.96) | 27.58(12.33-52.37) | -0.15(-0.16 to -0.14) | |
| **SDI Level** | | | |  | | | | |
| Low SDI | 39.64(31.03-48.06) | 38.48(30.23-46.44) | -0.1(-0.11 to -0.09) | | 36.17(16.44-69.57) | 35.24(16-67.46) | -0.09(-0.1 to -0.08) | |
| Low-middle SDI | 40.29(29.99-49.87) | 38.97(29.07-48.4) | -0.11(-0.13 to -0.09) | | 36.73(16.45-69.89) | 35.6(16.1-67.72) | -0.1(-0.12 to -0.08) | |
| Middle SDI | 30.15(21.34-39.07) | 30.04(21.68-38.33) | -0.02(-0.03 to 0) | | 27.79(12.32-52.57) | 27.66(12.33-52.57) | -0.02(-0.03 to 0) | |
| High-middle SDI | 31.33(21.97-40.67) | 29.05(21.25-37.03) | -0.25(-0.26 to -0.24) | | 29(12.86-55.24) | 26.9(11.99-50.91) | -0.25(-0.27 to -0.24) | |
| High SDI | 27.91(20.29-35.58) | 23.57(18.04-29.48) | -0.54(-0.56 to -0.53) | | 25.86(11.54-48.96) | 21.78(9.79-41.43) | -0.56(-0.57 to -0.55) | |
| **GBD Region** | | | | |  | | | |
| Andean Latin America | 51.15(38.47-62.91) | 50.07(37.3-61.83) | -0.07(-0.07 to -0.07) | | 47.55(21.34-89.02) | 46.41(20.96-87.93) | -0.08(-0.08 to -0.08) | |
| Australasia | 33.67(24.85-43.34) | 29.62(19.71-40.76) | -0.41(-0.44 to -0.39) | | 31.1(13.59-58.85) | 27.33(11.59-53.74) | -0.42(-0.45 to -0.39) | |
| Caribbean | 34.88(23.99-47.01) | 31.48(23.69-40.38) | -0.32(-0.33 to -0.31) | | 32.52(14.07-62.7) | 29.16(12.98-54.88) | -0.34(-0.35 to -0.33) | |
| Central Asia | 36.96(25.41-49.04) | 36.46(25.1-48.7) | -0.05(-0.06 to -0.04) | | 34.42(15.12-66.83) | 33.92(14.86-65.98) | -0.06(-0.07 to -0.05) | |
| Central Europe | 33.37(23.9-43.73) | 31.8(22.67-42.07) | -0.16(-0.17 to -0.15) | | 30.74(13.54-58.6) | 29.39(12.91-55.61) | -0.15(-0.16 to -0.14) | |
| Central Latin America | 33.94(23.93-44.32) | 33.22(23.17-43.43) | -0.1(-0.14 to -0.08) | | 31.29(13.87-59.33) | 30.66(13.61-58.63) | -0.1(-0.14 to -0.08) | |
| Central Sub-Saharan Africa | 35.16(24.2-46.74) | 35.98(24.94-47.04) | 0.07(0.07 to 0.08) | | 32.18(13.95-62.3) | 33.04(14.37-64.32) | 0.09(0.08 to 0.09) | |
| East Asia | 18.45(12.33-25.46) | 19(13.61-25.26) | 0.11(0.09 to 0.13) | | 17.17(7.36-33.5) | 17.67(7.73-34.13) | 0.1(0.09 to 0.12) | |
| Eastern Europe | 30.35(20.91-41.1) | 30.83(21.73-41.28) | 0.05(0.04 to 0.05) | | 27.96(12.1-54.22) | 28.37(12.46-54.84) | 0.04(0.04 to 0.05) | |
| Eastern Sub-Saharan Africa | 37.37(28.13-46) | 34.25(26.25-41.99) | -0.29(-0.3 to -0.28) | | 34.36(15.42-65.91) | 31.62(14.24-59.89) | -0.27(-0.28 to -0.26) | |
| High-income Asia Pacific | 10.3(6.98-14.69) | 8.36(5.9-11.5) | -0.68(-0.72 to -0.64) | | 9.58(4.01-18.62) | 7.82(3.33-15.09) | -0.66(-0.71 to -0.62) | |
| High-income North America | 19.38(12.47-28.05) | 19.47(13.26-27.05) | 0.01(0 to 0.02) | | 17.88(7.45-35.2) | 17.72(7.56-34.5) | -0.04(-0.04 to -0.03) | |
| North Africa and Middle East | 41.94(30.99-52.21) | 39.81(29.53-49.67) | -0.17(-0.18 to -0.15) | | 38.74(17.37-73.69) | 36.52(16.45-69.21) | -0.19(-0.2 to -0.17) | |
| Oceania | 44.31(32.52-55.11) | 43.78(32.14-54.54) | -0.03(-0.05 to -0.01) | | 40.77(17.9-77.52) | 40.14(17.47-76.04) | -0.04(-0.06 to -0.02) | |
| South Asia | 44.83(34.49-54.28) | 42.84(32.94-51.87) | -0.16(-0.21 to -0.12) | | 40.58(18.29-77.43) | 38.96(17.66-75.08) | -0.14(-0.19 to -0.11) | |
| Southeast Asia | 39.23(27.75-50.77) | 36.77(26.32-47.86) | -0.21(-0.22 to -0.19) | | 36.21(15.98-68.81) | 33.95(14.91-64.1) | -0.21(-0.22 to -0.2) | |
| Southern Latin America | 50.65(37.6-61.97) | 52.37(43.41-60.35) | 0.09(0.08 to 0.11) | | 47.13(20.89-88.18) | 48.62(22.53-90.75) | 0.08(0.06 to 0.1) | |
| Southern Sub-Saharan Africa | 26.61(18.23-35.84) | 25.81(18.11-34.25) | -0.09(-0.1 to -0.08) | | 24.59(10.69-47.75) | 23.64(10.24-45.89) | -0.12(-0.13 to -0.11) | |
| Tropical Latin America | 38.03(26.64-50.16) | 39.51(27.95-51.3) | 0.08(0.03 to 0.11) | | 34.78(15.11-65.61) | 36.14(15.84-68.11) | 0.1(0.05 to 0.16) | |
| Western Europe | 43.72(32.94-53.46) | 39.72(31.33-47.29) | -0.31(-0.32 to -0.29) | | 40.61(18.53-76.91) | 36.94(16.98-71.1) | -0.31(-0.32 to -0.29) | |
| Western Sub-Saharan Africa | 26.19(18.7-33.89) | 25.23(18.27-32.49) | -0.13(-0.14 to -0.12) | | 24.13(10.72-46.64) | 23.34(10.41-44.79) | -0.12(-0.13 to -0.1) | |

Data in parentheses are 95% confidence intervals.

ASR, age-standardized rate; AAPC, average annual percentage change; SDI, social-development index; DALYs, disability-adjusted life years.

**Table S2.** The prevalence and DALYs of edentulism and their trends from 1990 to 2021 at the global and regional levels

|  | **Prevalence** | | | **DALYs** | | |
| --- | --- | --- | --- | --- | --- | --- |
|  | ASR in 1990 (000s) | ASR in 2021 (000s) | AAPC  (1990-2021) | ASR in 1990 | ASR in 2021 | AAPC  (1990-2021) |
| **Sex** | | | | | | |
| Male | 27.81(20.79-35.55) | 24.65(19.55-30.46) | -0.37(-0.41 to -0.33) | 740.26(464.15-1095.32) | 655.83(420.97-941.51) | -0.37(-0.41 to -0.34) |
| Famle | 32.45(24.81-40.85) | 29.07(23.53-35.23) | -0.33(-0.37 to -0.3) | 862.24(550.84-1266.2) | 769(497.59-1090.57) | -0.35(-0.38 to -0.32) |
| **SDI Level** | | | | | | |
| Low SDI | 18.19(13.36-23.75) | 16.49(13.04-20.52) | -0.37(-0.45 to -0.3) | 473.8(294.48-701.85) | 431.2(277.33-624.16) | -0.35(-0.44 to -0.28) |
| Low-middle SDI | 25.39(18.92-32.61) | 22.7(18.45-27.39) | -0.39(-0.46 to -0.32) | 665.41(416.21-979.29) | 595.01(384.56-837.91) | -0.38(-0.46 to -0.31) |
| Middle SDI | 31.9(24.07-40.31) | 29.68(24.08-35.85) | -0.26(-0.32 to -0.21) | 847.84(535.75-1247.2) | 786.43(506.71-1113.7) | -0.27(-0.33 to -0.23) |
| High-middle SDI | 33.03(25.1-41.79) | 29.6(23.74-36.25) | -0.32(-0.37 to -0.28) | 880.66(557.31-1295.75) | 788.06(504.13-1125.72) | -0.36(-0.4 to -0.31) |
| High SDI | 31.65(24.17-40) | 26.75(20.88-33.37) | -0.55(-0.61 to -0.48) | 844.52(537.81-1243.93) | 710.63(455.7-1032.07) | -0.56(-0.62 to -0.5) |
| **GBD Region** | | | | | | |
| Andean Latin America | 59.73(49.48-69.18) | 56.31(47.1-64.99) | -0.19(-0.21 to -0.17) | 1613.97(1067.55-2275.9) | 1516.16(1008.99-2113.88) | -0.2(-0.22 to -0.19) |
| Australasia | 48.96(45.85-52.26) | 35.39(27.1-44.49) | -0.96(-1.1 to -0.85) | 1311.39(895.23-1805.98) | 945.77(600.09-1380.95) | -0.96(-1.07 to -0.85) |
| Caribbean | 36.13(27.39-45.84) | 33.62(25.68-42.47) | -0.22(-0.25 to -0.2) | 973.98(617.68-1429.68) | 899.18(573.94-1319.77) | -0.25(-0.27 to -0.23) |
| Central Asia | 34.46(26.05-43.65) | 31.92(23.98-40.66) | -0.24(-0.26 to -0.23) | 926.88(585.57-1357.12) | 856.12(539.06-1269.08) | -0.25(-0.27 to -0.24) |
| Central Europe | 41.97(32.3-52.17) | 36.94(28.14-46.52) | -0.39(-0.45 to -0.33) | 1112.01(715.39-1611.22) | 981.88(626.41-1440.54) | -0.38(-0.44 to -0.31) |
| Central Latin America | 35.64(27.09-45.07) | 34.12(28.29-40.92) | -0.16(-0.19 to -0.13) | 944.64(602.34-1384.62) | 907.48(579.59-1274.05) | -0.15(-0.2 to -0.1) |
| Central Sub-Saharan Africa | 16.02(11.98-20.77) | 16(12-20.76) | 0(-0.01 to 0.01) | 419.73(262.82-615.63) | 421.89(262.9-620.02) | 0.02(0.01 to 0.03) |
| East Asia | 28.61(20.76-37.3) | 26.74(20.35-33.87) | -0.23(-0.3 to -0.17) | 764.61(472.83-1141.93) | 710.92(445.92-1035.45) | -0.25(-0.33 to -0.19) |
| Eastern Europe | 36.5(27.5-45.96) | 38.85(30.48-48.02) | 0.19(0.18 to 0.2) | 968.84(614.31-1417.68) | 1031.03(641.89-1451.64) | 0.19(0.18 to 0.2) |
| Eastern Sub-Saharan Africa | 10.52(7.65-14.07) | 10.05(7.36-13.37) | -0.15(-0.16 to -0.14) | 275.47(168.23-414.25) | 264.22(162-396.77) | -0.14(-0.15 to -0.12) |
| High-income Asia Pacific | 25.91(18.84-33.77) | 19.75(14.87-25.35) | -0.74(-0.99 to -0.49) | 692.63(429-1041.97) | 529.85(330.66-782.22) | -0.73(-0.98 to -0.48) |
| High-income North America | 31.74(23.52-41.33) | 25.94(19.98-32.86) | -0.63(-0.72 to -0.52) | 843.91(525.79-1247.74) | 678.18(436.63-983.37) | -0.68(-0.77 to -0.59) |
| North Africa and Middle East | 37.12(28.39-46.64) | 34.66(27.49-42.34) | -0.23(-0.25 to -0.22) | 991.33(630.88-1443.88) | 918.29(593.06-1313.53) | -0.26(-0.27 to -0.25) |
| Oceania | 30.2(22.02-39.08) | 28.72(20.84-37.36) | -0.16(-0.17 to -0.16) | 797.28(491.26-1185.96) | 756.34(468.17-1127.49) | -0.17(-0.18 to -0.17) |
| South Asia | 22.27(16.02-29.63) | 18.89(14.94-23.38) | -0.59(-0.73 to -0.44) | 574.88(350.79-858.16) | 489(311.93-693.89) | -0.57(-0.73 to -0.43) |
| Southeast Asia | 28.95(21.52-37.29) | 25.71(20.69-31.63) | -0.38(-0.39 to -0.37) | 767.39(479.74-1139.58) | 681.87(437.5-969.78) | -0.38(-0.39 to -0.37) |
| Southern Latin America | 34.39(26.15-43.69) | 29.85(22.45-38.02) | -0.47(-0.47 to -0.46) | 925.37(583.68-1365.83) | 798.66(502.55-1179.37) | -0.48(-0.49 to -0.47) |
| Southern Sub-Saharan Africa | 23.84(18.34-30.18) | 22.24(16.93-28.3) | -0.19(-0.25 to -0.14) | 632.67(397.39-920.38) | 584.13(367.49-850.61) | -0.22(-0.28 to -0.17) |
| Tropical Latin America | 56.42(46.14-65.75) | 56.17(48.33-63.34) | -0.02(-0.04 to 0) | 1493.23(995.62-2100.16) | 1487.82(973.32-2021.25) | -0.02(-0.04 to 0) |
| Western Europe | 32.24(24.86-40.37) | 29.07(22.5-36.31) | -0.29(-0.4 to -0.2) | 863.57(547.79-1266.86) | 778.49(495.31-1138.08) | -0.3(-0.38 to -0.23) |
| Western Sub-Saharan Africa | 13.09(9.58-17.35) | 12.29(9.75-15.34) | -0.23(-0.27 to -0.2) | 344.48(212.58-514.92) | 325.18(208.56-470.8) | -0.2(-0.22 to -0.17) |

Data in parentheses are 95% confidence intervals.

ASR, age-standardized rate; AAPC, average annual percentage change; SDI, social-development index; DALYs, disability-adjusted life years.

**Table S3. The prevalence and DALYs of periodontal diseases and their trends from 1990 to 2021 at the global and regional levels**

|  | **Prevalence** | | | **DALYs** | | |
| --- | --- | --- | --- | --- | --- | --- |
|  | ASR in 1990 (000s) | ASR in 2021 (000s) | AAPC  (1990-2021) | ASR in 1990 | ASR in 2021 | AAPC  (1990-2021) |
| **Sex** | | | | | | |
| Male | 25.29(18.68-32.07) | 25.78(20.62-30.97) | 0.03(0.01 to 0.06) | 157.95(60.22-337.4) | 160.76(61.72-329.95) | 0.03(0.01 to 0.05) |
| Famle | 23.12(17.03-29.57) | 23.88(19.03-28.88) | 0.1(0.07 to 0.12) | 143.6(54.84-306.85) | 147.64(56.49-304.32) | 0.07(0.05 to 0.1) |
| **SDI Level** | | | | | | |
| Low SDI | 30.56(23.07-37.89) | 26.76(21.03-32.18) | -0.44(-0.47 to -0.41) | 188.34(73.15-396.67) | 164.91(62.7-339.01) | -0.44(-0.47 to -0.41) |
| Low-middle SDI | 27.01(20.04-33.95) | 28.14(22.68-33.65) | 0.14(0.11 to 0.17) | 166.42(64.25-354.04) | 173.22(66.54-349.91) | 0.14(0.11 to 0.16) |
| Middle SDI | 24.24(17.88-30.99) | 24.81(20.07-29.69) | 0.08(0.04 to 0.12) | 151.18(57.81-322.51) | 154.31(60.06-315.24) | 0.07(0.03 to 0.11) |
| High-middle SDI | 22.93(16.45-29.66) | 23.17(18.23-28.43) | -0.02(-0.06 to 0.02) | 143.28(54.44-306.58) | 144.56(54.89-298.27) | -0.02(-0.05 to 0.02) |
| High SDI | 22.41(16.61-28.6) | 23.52(18.16-29.04) | 0.15(0.13 to 0.18) | 139.95(53.11-297.19) | 146.06(55.29-301.49) | 0.14(0.11 to 0.16) |
| **GBD Region** | | | | | | |
| Andean Latin America | 14.24(10.94-18.06) | 14.12(9.83-19.48) | 0(-0.03 to 0.03) | 89.58(34.44-187.65) | 88.41(32.62-187.4) | -0.01(-0.05 to 0.02) |
| Australasia | 14.98(9.94-19.99) | 18.34(12.22-24.86) | 0.58(0.4 to 0.82) | 93.11(34.59-200.14) | 113.93(41.1-240.46) | 0.68(0.58 to 0.78) |
| Caribbean | 24.53(18.16-31.23) | 23.9(17.49-30.53) | -0.09(-0.1 to -0.07) | 154.46(59.52-328.87) | 149.08(55.64-305.65) | -0.1(-0.12 to -0.09) |
| Central Asia | 22.89(16.43-29.59) | 18.17(12.6-24.38) | -0.74(-0.78 to -0.71) | 143.65(54.89-307.83) | 113.61(41.73-238.13) | -0.76(-0.8 to -0.72) |
| Central Europe | 18.79(13.07-25.23) | 20.59(14.65-26.89) | 0.3(0.25 to 0.34) | 116.51(44.12-251.19) | 127.7(47.51-265.82) | 0.3(0.25 to 0.34) |
| Central Latin America | 24.2(17.9-30.81) | 24.48(19.21-30.08) | 0.04(0.02 to 0.06) | 150.44(58.06-320.13) | 152.16(58.33-308.21) | 0.04(0.03 to 0.05) |
| Central Sub-Saharan Africa | 31.78(24.1-39.05) | 23.12(15.11-31.15) | -1.03(-1.07 to -0.99) | 195.92(76.8-408.53) | 142.47(51.16-302.31) | -1.02(-1.06 to -0.98) |
| East Asia | 25.64(18.81-32.8) | 25.94(20.75-31.23) | 0.05(-0.02 to 0.11) | 161.1(61.49-343.37) | 162.53(62.7-337.23) | 0.04(-0.03 to 0.09) |
| Eastern Europe | 23.28(17.02-30) | 21.61(15.59-28.02) | -0.24(-0.25 to -0.23) | 144.56(55.45-308.44) | 133.67(49.65-275.6) | -0.25(-0.25 to -0.24) |
| Eastern Sub-Saharan Africa | 34.77(26.75-42.4) | 28.37(21.96-34.46) | -0.66(-0.67 to -0.64) | 215.3(84.22-450.02) | 175.44(66.45-357.33) | -0.65(-0.67 to -0.64) |
| High-income Asia Pacific | 21.51(14.35-28.88) | 24.07(16.63-31.2) | 0.37(0.28 to 0.47) | 135.3(49.99-294.03) | 151.37(54.9-318.05) | 0.33(0.24 to 0.44) |
| High-income North America | 25.13(18.82-31.89) | 24.7(19.55-29.79) | -0.12(-0.17 to -0.05) | 156.33(59.69-332.26) | 151.17(58.02-311.78) | -0.17(-0.22 to -0.11) |
| North Africa and Middle East | 20.82(14.64-27.38) | 22.84(17.81-28.12) | 0.31(0.29 to 0.32) | 129.58(49.02-279.4) | 140.98(53.73-286.76) | 0.28(0.26 to 0.3) |
| Oceania | 23.4(16.42-30.49) | 6.45(3.71-10.05) | -4.06(-4.25 to -3.9) | 145.35(54.04-311.24) | 39.69(13.16-91.57) | -4.09(-4.21 to -3.97) |
| South Asia | 28.58(21.21-35.77) | 30.96(25.06-36.78) | 0.27(0.22 to 0.31) | 174.85(68.11-371.47) | 189.77(73.13-381.18) | 0.28(0.23 to 0.32) |
| Southeast Asia | 23.24(16.8-30.1) | 24.38(19.59-29.2) | 0.15(0.14 to 0.17) | 144.65(54.64-307.72) | 151.44(58.64-310.54) | 0.15(0.13 to 0.16) |
| Southern Latin America | 22.45(15.89-29.4) | 23.72(16.83-30.51) | 0.17(0.13 to 0.21) | 140.89(53.2-301.7) | 147.87(54.98-305.7) | 0.15(0.12 to 0.18) |
| Southern Sub-Saharan Africa | 20.24(13.09-27.67) | 16.44(11.26-22.08) | -0.69(-0.71 to -0.66) | 125.86(46.55-273.24) | 100.92(36.04-219.59) | -0.71(-0.73 to -0.68) |
| Tropical Latin America | 16.12(11.47-21.84) | 16.74(12.76-21.28) | 0.12(0.09 to 0.16) | 99.68(38.29-217.32) | 103.49(40.24-216.31) | 0.12(0.09 to 0.16) |
| Western Europe | 20.53(15.15-26.36) | 20.02(14.39-25.76) | -0.1(-0.16 to -0.04) | 128.35(48.57-272.16) | 124.91(46.52-261.46) | -0.1(-0.15 to -0.06) |
| Western Sub-Saharan Africa | 35.35(27.62-42.79) | 24.58(19.35-29.66) | -1.15(-1.17 to -1.12) | 219.09(85.53-456.71) | 152.8(57.5-310.53) | -1.14(-1.16 to -1.11) |

Data in parentheses are 95% confidence intervals.

ASR, age-standardized rate; AAPC, average annual percentage change; SDI, social-development index; DALYs, disability-adjusted life years.

**Table S4. The prevalence and DALYs of other oral diseases and their trends from 1990 to 2021 at the global and regional levels**

|  | **Prevalence** | | | **DALYs** | | | |
| --- | --- | --- | --- | --- | --- | --- | --- |
|  | ASR in 1990 (000s) | ASR in 2021 (000s) | AAPC  (1990-2021) | ASR in 1990 | ASR in 2021 | AAPC  (1990-2021) | |
| **Sex** | | | | | | |  |
| Male | 1.93(1.7-2.15) | 1.93(1.7-2.15) | -0.01(-0.01 to 0) | 52.67(32.29-78.91) | 52.6(32.1-78.89) | 0(0 to 0) | |
| Famle | 2.23(1.98-2.47) | 2.23(1.98-2.48) | 0.01(0.01 to 0.01) | 60.34(37.25-90.15) | 60.36(36.93-90.32) | 0(0 to 0) | |
| **SDI Level** | | | | | | |  |
| Low SDI | 2.08(1.85-2.31) | 2.09(1.85-2.31) | 0.01(0.01 to 0.01) | 55.8(34.29-83.39) | 56.21(34.61-83.7) | 0.02(0.02 to 0.02) | |
| Low-middle SDI | 2.08(1.85-2.31) | 2.09(1.86-2.31) | 0.02(0.01 to 0.02) | 55.96(34.48-83.62) | 56.28(34.61-83.96) | 0.02(0.02 to 0.02) | |
| Middle SDI | 2.1(1.86-2.33) | 2.1(1.86-2.33) | NA | 57.07(35.12-85.99) | 56.91(35-85.59) | -0.01(-0.01 to -0.01) | |
| High-middle SDI | 2.12(1.88-2.35) | 2.1(1.87-2.34) | -0.02(-0.02 to -0.02) | 57.63(35.47-86.76) | 57.3(35.16-85.86) | -0.02(-0.02 to -0.01) | |
| High SDI | 2.1(1.87-2.33) | 2.09(1.86-2.33) | -0.02(-0.02 to -0.02) | 57.14(35.09-85.22) | 56.66(34.87-84.34) | -0.03(-0.03 to -0.03) | |
| **GBD Region** | | | | | | |  |
| Andean Latin America | 2.08(1.83-2.33) | 2.08(1.83-2.33) | NA | 57.01(34.76-85.31) | 56.82(34.68-85.4) | -0.01(-0.01 to 0) | |
| Australasia | 2.09(1.86-2.33) | 2.08(1.85-2.31) | -0.02(-0.02 to -0.02) | 56.73(34.42-86.1) | 56.6(34.52-84.63) | -0.01(-0.02 to -0.01) | |
| Caribbean | 2.08(1.83-2.33) | 2.09(1.83-2.34) | 0.01(0.01 to 0.01) | 57.16(34.64-85.31) | 56.82(34.8-85.22) | -0.02(-0.02 to -0.02) | |
| Central Asia | 2.13(1.87-2.39) | 2.1(1.85-2.36) | -0.03(-0.04 to -0.03) | 58.25(35.32-87.79) | 57.45(34.96-85.66) | -0.04(-0.04 to -0.04) | |
| Central Europe | 2.11(1.87-2.35) | 2.11(1.87-2.35) | -0.01(-0.01 to -0.01) | 57.01(35-85.1) | 57.11(35.09-85.04) | 0(0 to 0.01) | |
| Central Latin America | 2.09(1.85-2.32) | 2.1(1.86-2.33) | 0.01(0.01 to 0.01) | 56.56(34.84-84.67) | 56.91(34.87-85.32) | 0.02(0.02 to 0.02) | |
| Central Sub-Saharan Africa | 2.08(1.83-2.33) | 2.1(1.85-2.35) | 0.03(0.03 to 0.03) | 56.04(33.94-83.64) | 56.78(34.69-84.1) | 0.04(0.04 to 0.04) | |
| East Asia | 2.1(1.86-2.35) | 2.1(1.85-2.34) | -0.01(-0.01 to -0.01) | 57.59(35.32-87.13) | 57.32(34.98-86.53) | -0.01(-0.02 to -0.01) | |
| Eastern Europe | 2.16(1.91-2.41) | 2.14(1.89-2.38) | -0.03(-0.03 to -0.03) | 58.49(36.17-87.52) | 57.71(35.34-86.8) | -0.04(-0.05 to -0.04) | |
| Eastern Sub-Saharan Africa | 2.08(1.84-2.31) | 2.09(1.86-2.33) | 0.02(0.02 to 0.02) | 56.15(34.65-84.27) | 56.72(34.94-84.59) | 0.03(0.03 to 0.04) | |
| High-income Asia Pacific | 2.1(1.87-2.36) | 2.09(1.86-2.34) | -0.02(-0.02 to -0.02) | 57.69(35.35-86.07) | 57.6(34.91-86.33) | -0.01(-0.01 to 0) | |
| High-income North America | 2.09(1.85-2.33) | 2.08(1.84-2.32) | -0.02(-0.03 to -0.01) | 56.58(34.83-84.68) | 55.56(34.27-82.45) | -0.06(-0.07 to -0.05) | |
| North Africa and Middle East | 2.08(1.83-2.32) | 2.08(1.83-2.32) | NA | 56.36(34.63-84.3) | 55.99(34.37-83.07) | -0.02(-0.02 to -0.02) | |
| Oceania | 2.07(1.82-2.32) | 2.07(1.82-2.32) | NA | 56.03(34-83.5) | 55.8(34.33-83.21) | -0.01(-0.01 to -0.01) | |
| South Asia | 2.08(1.84-2.32) | 2.09(1.85-2.33) | 0.02(0.02 to 0.02) | 55.41(33.99-83.05) | 55.96(34.52-83.65) | 0.03(0.03 to 0.03) | |
| Southeast Asia | 2.1(1.86-2.33) | 2.1(1.86-2.33) | NA | 56.96(34.95-85.61) | 56.99(34.97-85.26) | 0(0 to 0) | |
| Southern Latin America | 2.09(1.84-2.33) | 2.09(1.84-2.33) | NA | 57.2(34.41-85.87) | 56.88(34.2-84.56) | -0.01(-0.02 to 0) | |
| Southern Sub-Saharan Africa | 2.12(1.87-2.35) | 2.12(1.88-2.36) | 0.01(0.01 to 0.01) | 57.46(35.34-86.53) | 57.13(35.24-85.55) | -0.02(-0.02 to -0.01) | |
| Tropical Latin America | 2.1(1.86-2.34) | 2.11(1.86-2.35) | 0.01(0.01 to 0.01) | 56.56(34.58-84.82) | 56.74(34.87-84.6) | 0.01(0.01 to 0.01) | |
| Western Europe | 2.1(1.86-2.34) | 2.09(1.85-2.32) | -0.02(-0.02 to -0.02) | 57.31(34.99-85.43) | 56.99(34.7-85.04) | -0.02(-0.02 to -0.02) | |
| Western Sub-Saharan Africa | 2.09(1.85-2.32) | 2.09(1.85-2.32) | NA | 56.46(34.85-84.4) | 56.74(34.75-84.97) | 0.02(0.01 to 0.02) | |

Data in parentheses are 95% confidence intervals.

ASR, age-standardized rate; AAPC, average annual percentage change; SDI, social-development index; NA, not available; DALYs, disability-adjusted life years.

**Table S5. The prevalence and DALYs of different oral diseases and their trends from 1990 to 2021 among different age groups**

|  | **Prevalence** | | | **DALYs** | | | |
| --- | --- | --- | --- | --- | --- | --- | --- |
|  | ASR in 1990 (000s) | ASR in 2021 (000s) | AAPC  (1990-2021) | ASR in 1990 | ASR in 2021 | AAPC  (1990-2021) | |
| **Permanent tooth caries** | | | | | | |  |
| 65 to 69 | 34.17(25.55-42.48) | 32.43(24.65-40.03) | -0.17(-0.19, -0.16) | 32.18(14.48-61.72) | 30.53(13.73-59.55) | -0.17(-0.19, -0.16) | |
| 70 to 74 | 33.12(22.96-41.53) | 31.74(22.89-39.36) | -0.14(-0.15, -0.13) | 30.74(13.78-57.31) | 29.43(13.04-54.66) | -0.15(-0.16, -0.14) | |
| 75 to 79 | 31.05(23.47-39.16) | 29.44(23.19-36.71) | -0.17(-0.18, -0.16) | 28.28(12.68-52.77) | 26.76(12.14-49.55) | -0.18(-0.19, -0.17) | |
| 80 to 84 | 28.79(20.43-38.24) | 26.61(19.39-34.59) | -0.25(-0.27, -0.24) | 25.73(11.35-48.06) | 23.77(10.54-44.94) | -0.25(-0.26, -0.24) | |
| 85 to 89 | 25.49(17.09-35.13) | 23.02(15.87-30.7) | -0.32(-0.33, -0.31) | 22.45(9.69-44.54) | 20.24(8.95-38.77) | -0.33(-0.34, -0.32) | |
| 90 to 94 | 21.05(12.46-31.14) | 18.85(11.82-26.79) | -0.36(-0.37, -0.34) | 18.2(7.4-37.26) | 16.29(6.83-33.12) | -0.35(-0.37, -0.34) | |
| 95 plus | 14.97(8.4-23.47) | 13.45(8.07-20.34) | -0.35(-0.36, -0.33) | 12.62(5.23-27.33) | 11.35(4.76-24.23) | -0.34(-0.36, -0.33) | |
| **Periodontal diseases** | | | | | | | |
| 65 to 69 | 27.41(20.84-34.55) | 27.65(22.64-32.53) | -0.01(-0.03, 0.02) | 173.94(65.11-377.18) | 174.91(66.85-358.5) | -0.02(-0.05, 0.01) | |
| 70 to 74 | 24.87(18.14-31.58) | 25.36(20.24-30.68) | 0.04(-0.01, 0.06) | 155.65(59.83-337.4) | 158.2(60.96-316.8) | 0(-0.03, 0.03) | |
| 75 to 79 | 22.17(16.2-28.26) | 23.46(18.48-28.15) | 0.16(0.11, 0.21) | 136.79(52.61-284.09) | 144.29(54.53-302.04) | 0.14(0.12, 0.16) | |
| 80 to 84 | 20.31(14.52-26.32) | 21.51(16.6-26.63) | 0.18(0.15, 0.2) | 122.98(47.86-251.75) | 130.31(51.18-274.41) | 0.18(0.16, 0.19) | |
| 85 to 89 | 19.14(13.84-25.05) | 20.07(15.2-25.39) | 0.13(0.11, 0.15) | 113.87(44.35-233.34) | 119.47(46.1-255.81) | 0.13(0.12, 0.15) | |
| 90 to 94 | 18.62(12.62-24.31) | 19.49(14.54-25.11) | 0.14(0.13, 0.16) | 108.83(42.99-240.34) | 113.73(43.19-239.57) | 0.14(0.13, 0.15) | |
| 95 plus | 18.41(11.34-24.6) | 19.38(13.99-25.33) | 0.16(0.13, 0.18) | 105.62(39.14-234.99) | 110.68(43.68-231.45) | 0.15(0.13, 0.17) | |
| **Edentulism** | | | | | | | |
| 65 to 69 | 21.61(15.89-28.27) | 18.84(14.91-23.66) | -0.43(-0.47, -0.39) | 590.57(354.86-890.37) | 513.75(323.54-738.72) | -0.44(-0.47, -0.4) | |
| 70 to 74 | 28.65(21.32-36.97) | 25.15(20-31.55) | -0.41(-0.45, -0.36) | 772.63(488.66-1159.14) | 676.72(439.71-973.1) | -0.41(-0.46, -0.37) | |
| 75 to 79 | 35.39(27.59-44) | 30.88(25.02-37.22) | -0.41(-0.44, -0.39) | 939.74(618.76-1371.47) | 817.61(528.5-1176.49) | -0.42(-0.45, -0.4) | |
| 80 to 84 | 40.22(31.23-49.64) | 36.56(29.93-43.55) | -0.28(-0.3, -0.25) | 1049.55(682.6-1525.38) | 952.1(617.95-1354.47) | -0.28(-0.31, -0.25) | |
| 85 to 89 | 43.05(33.23-52.23) | 39.91(32.68-47.17) | -0.23(-0.26, -0.21) | 1101.61(715.48-1556.28) | 1019(669.27-1408.01) | -0.24(-0.26, -0.22) | |
| 90 to 94 | 43.98(33.62-53.94) | 41.43(33.55-49.26) | -0.18(-0.2, -0.16) | 1102.85(704.45-1559.69) | 1037.23(683.9-1429.22) | -0.19(-0.21, -0.16) | |
| 95 plus | 44.33(33.28-55.4) | 41.82(33.11-50.74) | -0.16(-0.19, -0.14) | 1087.5(689.59-1536.4) | 1024(669.23-1426.6) | -0.17(-0.2, -0.15) | |
| **Other oral disorders** | | | | | | | |
| 65 to 69 | 2.43(2.17-2.69) | 2.43(2.16-2.69) | 0(0, 0) | 67.23(41.79-101.05) | 67.15(41.55-100.9) | 0(-0.01, 0) | |
| 70 to 74 | 2.29(2.02-2.54) | 2.28(2.01-2.54) | 0(0, 0) | 62.33(38.83-95.01) | 62.17(38.72-94.66) | -0.01(-0.01, -0.01) | |
| 75 to 79 | 2.02(1.81-2.22) | 2(1.8-2.21) | -0.02(-0.02, -0.02) | 54.08(32.94-81.16) | 53.68(32.56-80.46) | -0.03(-0.03, -0.02) | |
| 80 to 84 | 1.68(1.48-1.88) | 1.67(1.47-1.87) | -0.02(-0.03, -0.02) | 44.42(26.65-64.69) | 44(26.5-64.1) | -0.03(-0.03, -0.03) | |
| 85 to 89 | 1.29(1.15-1.44) | 1.28(1.14-1.42) | -0.03(-0.03, -0.03) | 33.34(20.69-47.68) | 32.98(20.56-47) | -0.03(-0.04, -0.03) | |
| 90 to 94 | 1.11(0.97-1.25) | 1.1(0.96-1.24) | -0.03(-0.03, -0.03) | 28.12(17.8-40.26) | 27.81(17.7-39.85) | -0.04(-0.04, -0.03) | |
| 95 plus | 1.15(0.97-1.32) | 1.14(0.96-1.31) | -0.03(-0.03, -0.02) | 28.48(17.85-42.06) | 28.18(17.84-41.6) | -0.04(-0.04, -0.03) | |

Data in parentheses are 95% uncertainty intervals for ASR and 95% confidence intervals for AAPCs.

ASR, age-standardized rate; AAPC, average annual percentage change; NA, not available; DALYs, disability-adjusted life years.

**Table S6** The Joinpoint analysis results in prevalence of oral diseases among 21 GBD regions

| **Oral diseases** | | | | | | | | | | | | | |
| --- | --- | --- | --- | --- | --- | --- | --- | --- | --- | --- | --- | --- | --- |
| Location | N | Segment 1 | | Segment 2 | | Segment 3 | | Segment 4 | | Segment 5 | | Segment 6 | |
|  |  | Period | APC  (95% CI) | Period | APC  (95% CI) | Period | APC  (95% CI) | Period | APC  (95% CI) | Period | APC  (95% CI) | Period | APC  (95% CI) |
| Andean Latin America | 5 | 1990-2001 | -0.08(-0.09, -0.07) | 2001-2004 | -0.61(-0.66, -0.5) | 2004-2009 | 0.03(-0.04, 0.08) | 2009-2015 | 0.27(0.24, 0.33) | 2015-2019 | -0.35(-0.42, -0.31) | 2019-2021 | 0.09(-0.06, 0.19) |
| Australasia | 5 | 2001-2004 | -5.53(-5.87, -5.21) | 1992-1995 | -3.31(-3.58, -3.06) | 1995-2000 | 1.7(1.54, 1.87) | 2000-2005 | -0.78(-0.99, -0.63) | 2005-2009 | 0.83(0.62, 1.13) | 2009-2021 | 0.12(0.05, 0.16) |
| Caribbean | 5 | 2004-2009 | -0.15(-0.17, -0.13) | 1996-1999 | -0.42(-0.45, -0.35) | 1999-2005 | -0.08(-0.1, -0.05) | 2005-2010 | -0.38(-0.41, -0.35) | 2010-2017 | -0.07(-0.1, -0.05) | 2017-2021 | 0.07(0.02, 0.14) |
| Central Asia | 3 | 2009-2015 | 0.29(0.28, 0.31) | 2000-2005 | -0.85(-0.89, -0.82) | 2005-2015 | -0.37(-0.4, -0.34) | 2015-2021 | -0.07(-0.13, 0) |  |  |  |  |
| Central Europe | 5 | 2015-2019 | 0.07(0, 0.16) | 1995-2000 | -0.31(-0.45, -0.24) | 2000-2005 | 0.26(0.2, 0.36) | 2005-2009 | -0.68(-0.81, -0.59) | 2009-2018 | -0.31(-0.34, -0.26) | 2018-2021 | 0.43(0.27, 0.79) |
| Central Latin America | 2 | 2019-2021 | -0.12(-0.14, -0.11) | 2012-2019 | 0.29(0.24, 0.4) | 2019-2021 | -0.58(-0.9, -0.25) |  |  |  |  |  |  |
| Central Sub-Saharan Africa | 3 | 1990-1992 | 0.17(0.06, 0.36) | 1994-2005 | 0.03(-1.39, 0.05) | 2005-2010 | -1.37(-1.41, -0.08) | 2010-2021 | -0.06(-0.1, -0.03) |  |  |  |  |
| East Asia | 5 | 1992-1995 | -0.46(-0.49, -0.43) | 2000-2005 | 0.83(0.77, 0.89) | 2005-2010 | -0.76(-0.89, -0.68) | 2010-2015 | 0.01(-0.1, 0.15) | 2015-2019 | 0.85(0.75, 1.01) | 2019-2021 | -0.09(-0.32, 0.2) |
| Eastern Europe | 5 | 1995-2000 | 0.07(0.06, 0.08) | 2000-2005 | -0.26(-0.29, -0.23) | 2005-2010 | -0.06(-0.09, -0.01) | 2010-2015 | 0.22(0.19, 0.26) | 2015-2019 | 0.01(-0.04, 0.05) | 2019-2021 | 0.28(0.2, 0.35) |
| Eastern Sub-Saharan Africa | 2 | 2000-2005 | -0.21(-0.22, -0.2) | 2006-2009 | -1.29(-1.35, -1.24) | 2009-2021 | -0.13(-0.15, -0.11) |  |  |  |  |  |  |
| High-income Asia Pacific | 5 | 2005-2009 | -0.67(-1.17, -0.33) | 1995-2000 | 0.99(0.67, 1.69) | 2000-2008 | -0.47(-0.72, -0.23) | 2008-2015 | -1.86(-2.31, -1.63) | 2015-2019 | 0.38(-0.49, 1) | 2019-2021 | 3.82(2.45, 4.85) |
| High-income North America | 5 | 2009-2021 | -0.35(-0.41, -0.29) | 2001-2006 | -1.22(-1.3, -0.32) | 2006-2010 | -1.62(-1.84, -1.22) | 2010-2014 | 1.48(-1.53, 1.71) | 2014-2018 | 0.99(0.74, 1.32) | 2018-2021 | 0.16(-0.26, 0.4) |
| North Africa and Middle East | 3 | 1990-1996 | -0.22(-0.25, -0.18) | 1994-2005 | 0.09(0.08, 0.1) | 2005-2014 | -0.26(-0.27, -0.25) | 2014-2021 | 0(-0.02, 0.02) |  |  |  |  |
| Oceania | 3 | 1996-1999 | -0.05(-0.07, -0.02) | 2001-2004 | -2.21(-2.33, -2.1) | 2004-2010 | -0.55(-0.67, -0.45) | 2010-2021 | -0.09(-0.13, -0.03) |  |  |  |  |
| South Asia | 3 | 1999-2005 | -0.1(-0.2, -0.03) | 2000-2010 | 0.91(0.86, 0.99) | 2010-2019 | -1.18(-1.26, -1.12) | 2019-2021 | 0.11(-0.56, 0.45) |  |  |  |  |
| Southeast Asia | 5 | 2005-2010 | -0.11(-0.12, -0.1) | 2000-2006 | -0.19(-0.21, -0.17) | 2006-2010 | -0.32(-0.35, -0.3) | 2010-2015 | 0.11(0.1, 0.14) | 2015-2019 | -0.18(-0.23, -0.16) | 2019-2021 | 0.11(0.02, 0.16) |
| Southern Latin America | 5 | 2010-2017 | -0.05(-0.1, 0) | 1995-2000 | -0.55(-0.59, -0.5) | 2000-2005 | 0.27(0.22, 0.32) | 2005-2010 | -0.07(-0.19, -0.01) | 2010-2015 | 0.25(0.19, 0.37) | 2015-2021 | 0.01(-0.04, 0.05) |
| Southern Sub-Saharan Africa | 5 | 2017-2021 | -0.38(-0.54, -0.27) | 1995-2000 | 0.33(0.23, 0.45) | 2000-2005 | -1.44(-1.56, -1.35) | 2005-2010 | -0.49(-0.61, -0.35) | 2010-2015 | 0.79(0.67, 0.96) | 2015-2021 | -0.01(-0.13, 0.08) |
| Tropical Latin America | 3 | 1990-2000 | -0.06(-0.09, -0.04) | 2001-2005 | 0.77(0.72, 0.82) | 2005-2015 | -0.19(-0.22, -0.17) | 2015-2021 | -0.01(-0.06, 0.09) |  |  |  |  |
| Western Europe | 5 | 2000-2005 | -0.65(-0.94, -0.45) | 1993-2001 | -0.1(-0.14, -0.03) | 2001-2005 | -0.59(-0.75, -0.41) | 2005-2010 | -0.99(-1.1, -0.92) | 2010-2015 | 0.96(0.89, 1.02) | 2015-2021 | -0.02(-0.09, 0.04) |
| Western Sub-Saharan Africa | 5 | 2005-2015 | -0.12(-0.21, 0) | 1995-2006 | -0.47(-0.51, -0.44) | 2006-2009 | -2.37(-2.46, -0.49) | 2009-2015 | -0.52(-2.33, -0.46) | 2015-2019 | 0.31(-0.48, 0.49) | 2019-2021 | -0.19(-0.43, 0.15) |
| **Dental caries** | | | | | | | | | | | | | |
| Andean Latin America | 5 | 1990-1994 | -0.12(-0.17, -0.09) | 1994-2000 | -0.01(-0.03, 0.02) | 2000-2005 | -0.13(-0.16, -0.1) | 2005-2015 | 0.01(0, 0.02) | 2015-2019 | -0.35(-0.36, -0.33) | 2019-2021 | 0.15(0.09, 0.19) |
| Australasia | 4 | 1990-1995 | -1.53(-1.77, -1.28) | 1995-2000 | -2.99(-3.2, -2.8) | 2000-2011 | -0.12(-0.19, -0.06) | 2011-2014 | 3.89(3.61, 4.13) | 2014-2021 | 0(-0.16, 0.14) |  |  |
| Caribbean | 3 | 1990-1993 | -0.51(-0.74, -0.37) | 1993-2005 | -0.16(-0.18, -0.14) | 2005-2014 | -0.67(-0.69, -0.65) | 2014-2021 | -0.08(-0.1, -0.05) |  |  |  |  |
| Central Asia | 4 | 1990-1995 | 0.25(0.09, 0.32) | 1995-2000 | 0.38(-0.24, 0.47) | 2000-2011 | -0.25(-0.57, -0.22) | 2011-2015 | -0.59(-0.72, -0.08) | 2015-2021 | 0.05(-0.01, 0.12) |  |  |
| Central Europe | 5 | 1990-1994 | 0.23(0.15, 0.31) | 1994-2000 | -0.08(-0.16, -0.04) | 2000-2005 | 0.35(0.31, 0.41) | 2005-2015 | -0.37(-0.38, -0.35) | 2015-2019 | -0.71(-0.82, -0.63) | 2019-2021 | -0.31(-0.49, -0.18) |
| Central Latin America | 3 | 1990-2005 | -0.23(-0.29, -0.19) | 2005-2009 | 1.17(0.86, 1.62) | 2009-2019 | -0.05(-0.11, 0.01) | 2019-2021 | -1.89(-2.47, -1.18) |  |  |  |  |
| Central Sub-Saharan Africa | 5 | 1990-1993 | 0.65(0.6, 0.72) | 1993-1996 | 0.46(0.36, 0.5) | 1996-2005 | 0.28(0.27, 0.29) | 2005-2010 | -0.08(-0.09, -0.06) | 2010-2015 | -0.36(-0.39, -0.34) | 2015-2021 | -0.22(-0.24, -0.2) |
| East Asia | 5 | 1990-1992 | 2.65(2.24, 3.02) | 1992-1995 | 1.37(0.84, 1.56) | 1995-2000 | -0.21(-0.4, -0.09) | 2000-2005 | 0.32(0.17, 0.53) | 2005-2014 | -0.67(-0.74, -0.61) | 2014-2021 | -0.08(-0.17, 0.02) |
| Eastern Europe | 5 | 1990-1994 | 0.29(0.24, 0.35) | 1994-1999 | 0.1(0.04, 0.14) | 1999-2005 | -0.2(-0.23, -0.17) | 2005-2010 | 0.17(0.13, 0.23) | 2010-2019 | -0.02(-0.06, 0) | 2019-2021 | 0.18(0.04, 0.24) |
| Eastern Sub-Saharan Africa | 4 | 1990-1995 | -0.43(-0.61, -0.35) | 1995-2005 | -0.15(-0.18, -0.11) | 2005-2010 | -0.58(-0.73, -0.5) | 2010-2016 | -0.09(-0.14, 0.08) | 2016-2021 | -0.35(-0.46, -0.29) |  |  |
| High-income Asia Pacific | 4 | 1990-1995 | -1.43(-1.93, -1.1) | 1995-2011 | 0.06(0, 0.12) | 2011-2014 | -5.96(-6.32, -5.65) | 2014-2017 | -0.77(-1.18, -0.22) | 2017-2021 | 1.5(1.13, 2.19) |  |  |
| High-income North America | 5 | 1990-1994 | 0.84(0.75, 0.94) | 1994-1998 | 0.37(0.27, 0.48) | 1998-2005 | -0.01(-0.05, 0.04) | 2005-2010 | -0.21(-0.3, -0.16) | 2010-2015 | -0.84(-0.88, -0.8) | 2015-2021 | 0.14(0.11, 0.18) |
| North Africa and Middle East | 5 | 1990-1992 | -0.79(-0.99, -0.47) | 1992-1995 | -0.34(-0.42, 0.47) | 1995-2000 | 0.41(-0.21, 0.56) | 2000-2006 | -0.19(-0.85, -0.12) | 2006-2010 | -0.84(-0.94, 0) | 2010-2021 | -0.02(-0.06, 0.03) |
| Oceania | 3 | 1990-1996 | -0.1(-0.4, 0.03) | 1996-2005 | 0.22(0.15, 0.5) | 2005-2009 | -0.44(-0.7, -0.19) | 2009-2021 | -0.06(-0.1, 0.04) |  |  |  |  |
| South Asia | 4 | 1990-2001 | 0.02(-0.07, 0.11) | 2001-2004 | 4.07(3.82, 4.33) | 2004-2015 | -0.27(-0.34, -0.2) | 2015-2019 | -3.42(-3.79, -3.2) | 2019-2021 | -0.13(-1.08, 0.54) |  |  |
| Southeast Asia | 5 | 1990-1994 | -0.23(-0.44, -0.1) | 1994-2001 | 0.14(0.1, 0.22) | 2001-2010 | -0.68(-0.72, -0.65) | 2010-2015 | 0.08(0.01, 0.19) | 2015-2019 | -0.41(-0.6, -0.3) | 2019-2021 | 0.45(0.16, 0.67) |
| Southern Latin America | 3 | 1990-2005 | -0.17(-0.23, -0.13) | 2005-2010 | 0.36(-0.15, 0.56) | 2010-2015 | 0.73(0.53, 0.97) | 2015-2021 | 0(-0.11, 0.11) |  |  |  |  |
| Southern Sub-Saharan Africa | 4 | 1990-1994 | -0.89(-1.05, -0.8) | 1994-2000 | -0.35(-0.43, -0.28) | 2000-2006 | 0.24(0.1, 0.29) | 2006-2009 | 0.57(0.39, 0.65) | 2009-2021 | -0.02(-0.05, 0) |  |  |
| Tropical Latin America | 2 | 1990-2003 | -0.34(-0.47, -0.23) | 2003-2012 | 0.79(0.6, 1.35) | 2012-2021 | -0.03(-0.27, 0.14) |  |  |  |  |  |  |
| Western Europe | 5 | 1990-1994 | -1.74(-1.86, -1.61) | 1994-2000 | -0.23(-0.31, -0.14) | 2000-2005 | -0.74(-0.98, -0.64) | 2005-2010 | -0.09(-0.27, 0.09) | 2010-2014 | 0.65(0.49, 0.87) | 2014-2021 | 0.06(-0.02, 0.12) |
| Western Sub-Saharan Africa | 3 | 1990-1994 | -0.8(-0.97, -0.67) | 1994-2006 | 0.11(0.08, 0.15) | 2006-2009 | -0.39(-0.47, -0.16) | 2009-2021 | -0.08(-0.11, -0.01) |  |  |  |  |
| **Edentulous** | | | | | | | | | | | | | |
| Andean Latin America | 5 | 1990-2000 | -0.07(-0.12, -0.02) | 2000-2005 | -1.76(-1.85, -1.66) | 2005-2010 | 0.26(0.1, 0.38) | 2010-2015 | 1.26(1.16, 1.41) | 2015-2019 | -1.1(-1.3, -0.98) | 2019-2021 | 0.22(-0.24, 0.53) |
| Australasia | 5 | 1990-1994 | -11.58(-12.25, -11) | 1994-2000 | 3.89(3.05, 4.82) | 2000-2005 | -2.67(-4.21, -1.62) | 2005-2010 | 2.82(2.31, 3.58) | 2010-2015 | -3.51(-4.47, -2.85) | 2015-2021 | 2.36(1.29, 3.93) |
| Caribbean | 5 | 1990-1994 | 0.16(-0.04, 0.52) | 1994-2005 | -0.33(-0.38, -0.28) | 2005-2010 | -1.3(-1.4, -1.2) | 2010-2015 | 0.35(0.25, 0.51) | 2015-2019 | -0.28(-0.52, -0.14) | 2019-2021 | 0.98(0.53, 1.28) |
| Central Asia | 4 | 1990-2000 | 1.3(1.25, 1.35) | 2000-2006 | -0.83(-0.91, 1.29) | 2006-2013 | -1.43(-1.64, -0.83) | 2013-2017 | -0.95(-1.42, -0.7) | 2017-2021 | -0.4(-0.62, 0.02) |  |  |
| Central Europe | 5 | 1990-1995 | 0.2(-0.17, 1.13) | 1995-2000 | -0.92(-1.74, -0.51) | 2000-2005 | 0.84(0.46, 1.41) | 2005-2009 | -3.12(-3.67, -2.7) | 2009-2018 | -0.89(-1.06, -0.74) | 2018-2021 | 2.72(1.77, 4.43) |
| Central Latin America | 4 | 1990-2006 | -0.35(-0.41, -0.28) | 2006-2010 | -2.74(-2.92, -0.32) | 2010-2015 | 1.05(-2.94, 1.19) | 2015-2018 | 1.78(1.3, 2.03) | 2018-2021 | 0.4(-0.26, 0.75) |  |  |
| Central Sub-Saharan Africa | 5 | 1990-2000 | 0.77(0.73, 0.8) | 2000-2005 | -0.08(-0.13, 0.81) | 2005-2008 | -0.45(-0.67, -0.09) | 2008-2015 | -0.79(-0.91, -0.5) | 2015-2018 | -0.22(-0.77, -0.12) | 2018-2021 | 0.15(0, 0.35) |
| East Asia | 5 | 1990-2000 | -2.63(-2.84, -2.43) | 2000-2005 | 2.47(1.71, 3.31) | 2005-2010 | 1.11(0.14, 1.61) | 2010-2015 | -5.53(-5.94, -5.16) | 2015-2019 | 8.21(7.7, 8.77) | 2019-2021 | -0.73(-1.85, 0.42) |
| Eastern Europe | 5 | 1990-2000 | 0.3(0.27, 0.33) | 2000-2005 | -0.86(-0.91, -0.81) | 2005-2011 | 0.34(0.29, 0.39) | 2011-2014 | 1.03(0.93, 1.11) | 2014-2019 | 0.17(0.07, 0.22) | 2019-2021 | 0.7(0.45, 0.85) |
| Eastern Sub-Saharan Africa | 4 | 1990-2000 | 0.18(0.15, 0.21) | 2000-2005 | -1.24(-1.29, -1.18) | 2005-2010 | 0.76(0.69, 0.82) | 2010-2015 | -0.75(-0.84, -0.68) | 2015-2021 | -0.03(-0.1, 0.04) |  |  |
| High-income Asia Pacific | 4 | 1990-1994 | -5.82(-9.59, -3.43) | 1994-2005 | 3.4(2.85, 4.12) | 2005-2012 | -8.14(-9.48, -7.25) | 2012-2018 | -1.44(-3.27, 0.47) | 2018-2021 | 11.36(7.68, 17.89) |  |  |
| High-income North America | 5 | 1990-1994 | -2.99(-4.58, -2.09) | 1994-2001 | -0.48(-0.86, 0.33) | 2001-2005 | -5.49(-6.55, -4.59) | 2005-2011 | -1.88(-2.21, -1.45) | 2011-2019 | 3.72(3.48, 4.18) | 2019-2021 | 0.39(-0.98, 2.31) |
| North Africa and Middle East | 3 | 1990-2001 | -0.08(-0.14, -0.03) | 2001-2005 | 1.24(1.12, 1.39) | 2005-2014 | -1.19(-1.24, -1.14) | 2014-2021 | -0.08(-0.15, 0) |  |  |  |  |
| Oceania | 5 | 1990-1995 | -0.21(-0.24, -0.14) | 1995-2000 | -0.28(-0.32, -0.25) | 2000-2005 | 0.2(0.16, 0.23) | 2005-2010 | -0.06(-0.1, -0.03) | 2010-2019 | -0.41(-0.43, -0.4) | 2019-2021 | 0.17(0.11, 0.24) |
| South Asia | 3 | 1990-2005 | -1.14(-1.45, -0.82) | 2005-2010 | 15.19(14.13, 16.24) | 2010-2015 | -13.66(-14.7, -12.74) | 2015-2021 | 0.29(-0.7, 1.29) |  |  |  |  |
| Southeast Asia | 5 | 1990-1996 | -0.48(-0.59, -0.41) | 1996-2000 | -0.08(-0.25, 0.06) | 2000-2010 | -0.73(-0.76, -0.69) | 2010-2015 | 0.41(-0.72, 0.45) | 2015-2018 | -0.7(-0.79, 0.37) | 2018-2021 | -0.4(-0.57, -0.21) |
| Southern Latin America | 4 | 1990-1995 | -0.65(-0.72, -0.56) | 1995-1999 | -1.04(-1.14, -0.97) | 1999-2006 | 0(-0.03, 0.03) | 2006-2014 | -0.79(-0.82, -0.77) | 2014-2021 | -0.09(-0.13, -0.06) |  |  |
| Southern Sub-Saharan Africa | 5 | 1990-1995 | -0.91(-1.37, -0.49) | 1995-2000 | 3.54(3.19, 4) | 2000-2006 | -4.19(-4.76, -3.92) | 2006-2010 | -2.66(-3.23, -1.67) | 2010-2014 | 4.23(3.45, 5.03) | 2014-2021 | 0.16(-0.15, 0.44) |
| Tropical Latin America | 3 | 1990-2000 | -0.01(-0.08, 0.07) | 2000-2005 | 1.93(1.81, 2.06) | 2005-2014 | -1.04(-1.11, -0.98) | 2014-2021 | -0.09(-0.2, 0.07) |  |  |  |  |
| Western Europe | 5 | 1990-1995 | -0.76(-2.27, -0.11) | 1995-2000 | 1.36(0.75, 2.63) | 2000-2005 | -0.91(-1.8, -0.47) | 2005-2010 | -7.95(-8.3, -7.56) | 2010-2014 | 7.95(7.21, 8.52) | 2014-2021 | 0.48(-0.06, 0.92) |
| Western Sub-Saharan Africa | 4 | 1990-2000 | 0.49(0.36, 0.62) | 2000-2010 | -0.81(-0.92, -0.7) | 2010-2015 | -3.05(-3.23, -2.84) | 2015-2019 | 3.01(2.77, 3.29) | 2019-2021 | -0.18(-0.8, 0.62) |  |  |
| **Periodontal diseases** | | | | | | | | | | | | | |
| Andean Latin America | 5 | 1990-1993 | -2.64(-3, -2.26) | 1993-2000 | -0.56(-0.71, -0.4) | 2000-2005 | 2.23(1.98, 2.59) | 2005-2010 | 1(0.68, 1.25) | 2010-2014 | -1.93(-2.22, -1.63) | 0.53(0.37, 0.7) | 2014-2021 |
| Australasia | 5 | 1990-1992 | -7.24(-9.25, -3.15) | 1992-1995 | -2.54(-3.29, 7.92) | 1995-2001 | 7.36(-0.12, 8.57) | 2001-2011 | 0.09(-1.93, 2.98) | 2011-2015 | 3.33(-1.66, 5.17) | -2.65(-4.06, -1.56) | 2015-2021 |
| Caribbean | 5 | 1990-1996 | -0.6(-0.68, -0.51) | 1996-2000 | -1.23(-1.36, -1.07) | 2000-2005 | 0.36(0.11, 0.45) | 2005-2010 | 0.79(0.59, 1.01) | 2010-2019 | 0.09(0.05, 0.65) | -0.33(-0.56, 0.03) | 2019-2021 |
| Central Asia | 5 | 1990-1998 | -0.94(-1.04, -0.75) | 1998-2001 | -1.84(-2.16, -1.43) | 2001-2004 | -6.76(-7.15, -6.41) | 2004-2007 | -0.65(-0.97, -0.12) | 2007-2016 | 1.24(1.12, 1.55) | 0.39(-0.09, 0.68) | 2016-2021 |
| Central Europe | 4 | 1990-2001 | -0.07(-0.16, 0.07) | 2001-2005 | -1.19(-1.62, -0.88) | 2005-2009 | 2.93(2.53, 3.43) | 2009-2018 | 1(0.86, 1.15) | 2018-2021 | -1.91(-2.6, -1.16) |  |  |
| Central Latin America | 5 | 1990-1994 | -0.12(-0.42, 0.03) | 1994-2006 | 0.17(0.14, 0.25) | 2006-2009 | 1.54(0.15, 1.64) | 2009-2012 | 0.09(-0.06, 1.55) | 2012-2019 | -0.5(-0.53, 0.12) | 2019-2021 | -0.84(-1.06, -0.55) |
| Central Sub-Saharan Africa | 2 | 1990-2006 | -0.58(-0.65, -0.51) | 2006-2009 | -8.35(-8.73, -8) | 2009-2021 | 0.28(0.14, 0.41) |  |  |  |  |  |  |
| East Asia | 5 | 1990-1994 | -0.35(-1.63, 0.29) | 1994-2005 | 0.66(0.52, 1.03) | 2005-2010 | -3.19(-3.5, -2.87) | 2010-2015 | 5.32(5.03, 5.63) | 2015-2019 | -3.52(-4.05, -3.2) | 2019-2021 | 0.16(-1.01, 0.97) |
| Eastern Europe | 3 | 1990-2000 | -0.33(-0.37, -0.3) | 2000-2006 | 0.01(-0.04, 0.08) | 2006-2009 | -1.52(-1.6, -1.45) | 2009-2021 | 0.03(0.01, 0.05) |  |  |  |  |
| Eastern Sub-Saharan Africa | 5 | 1990-1996 | -0.2(-0.29, -0.08) | 1996-1999 | -1.27(-1.42, -0.93) | 1999-2006 | -0.32(-0.39, -0.22) | 2006-2009 | -3.81(-3.99, -3.66) | 2009-2012 | -0.49(-0.64, -0.25) | 2012-2021 | 0(-0.05, 0.13) |
| High-income Asia Pacific | 5 | 1990-1993 | 4.89(3.66, 6.87) | 1993-1996 | 0.71(-1.7, 2.11) | 1996-2001 | -2.5(-4.15, -1.94) | 2001-2005 | -4.47(-5.27, 7.26) | 2005-2009 | 7.44(-0.02, 8.02) | 2009-2021 | -0.23(-0.42, -0.06) |
| High-income North America | 5 | 1990-1995 | 1.14(0.68, 1.79) | 1995-2000 | -1.29(-2.4, -0.81) | 2000-2005 | 0.76(0.31, 1.63) | 2005-2010 | -3.28(-3.67, -2.91) | 2010-2015 | 3.4(3.05, 3.78) | 2015-2021 | -1.09(-1.41, -0.83) |
| North Africa and Middle East | 5 | 1990-1995 | -0.25(-0.43, -0.12) | 1995-2000 | 0.77(0.68, 0.89) | 2000-2005 | -0.79(-0.87, -0.71) | 2005-2010 | 1.36(1.28, 1.49) | 2010-2014 | 0.97(0.67, 1.08) | 2014-2021 | 0.03(-0.04, 0.09) |
| Oceania | 3 | 1990-2001 | 0(-0.39, 0.4) | 2001-2004 | -23.8(-24.95, -22.68) | 2004-2010 | -8.62(-10.16, -7.48) | 2010-2021 | 0.66(-0.09, 1.64) |  |  |  |  |
| South Asia | 3 | 1990-2005 | 0.38(0.28, 0.48) | 2005-2010 | -4.67(-4.94, -4.39) | 2010-2015 | 5.34(5.03, 5.66) | 2015-2021 | 0.07(-0.2, 0.32) |  |  |  |  |
| Southeast Asia | 5 | 1990-1995 | 0.44(0.32, 0.57) | 1995-2000 | -1.3(-1.39, -1.2) | 2000-2005 | 1.09(0.99, 1.21) | 2005-2010 | 0.5(0.38, 0.62) | 2010-2014 | -0.26(-0.45, -0.1) | 2014-2021 | 0.31(0.25, 0.4) |
| Southern Latin America | 5 | 1990-1995 | 0.86(0.64, 1.09) | 1995-2000 | -3.14(-3.3, -2.97) | 2000-2005 | 2.77(2.55, 2.96) | 2005-2010 | -0.44(-0.88, -0.15) | 2010-2015 | 0.99(0.67, 1.62) | 2015-2021 | 0.11(-0.37, 0.32) |
| Southern Sub-Saharan Africa | 3 | 1990-1996 | 0(-0.17, 0.18) | 1996-1999 | -3.19(-3.42, -2.74) | 1999-2004 | -2.23(-2.36, -1.13) | 2004-2021 | -0.03(-0.07, 0.01) |  |  |  |  |
| Tropical Latin America | 5 | 1990-1996 | -0.06(-0.26, 0.23) | 1996-2001 | -1.29(-1.69, -0.92) | 2001-2005 | -3.12(-3.52, -2.8) | 2005-2010 | 3.36(3.17, 3.56) | 2010-2019 | 0.84(0.76, 1.02) | 2019-2021 | -0.37(-0.81, 0.38) |
| Western Europe | 5 | 1990-1995 | 0.92(0.45, 1.34) | 1995-2000 | -2.26(-2.82, -1.9) | 2000-2005 | 0.06(-0.26, 0.54) | 2005-2010 | 4.08(3.79, 4.39) | 2010-2014 | -3.62(-4.25, -3.22) | 2014-2021 | -0.25(-0.53, 0.09) |
| Western Sub-Saharan Africa | 3 | 1990-1995 | -0.2(-0.45, 0.15) | 1995-2006 | -1.11(-1.2, -1.04) | 2006-2009 | -5.73(-5.92, -5.51) | 2009-2021 | -0.4(-0.46, -0.34) |  |  |  |  |
| **The other oral disease** | | | | | | | | | | | | | |
| Andean Latin America | 2 | 1990-2011 | 0(0, 0) | 2011-2019 | 0(0, 0.01) | 2019-2021 | 0.05(0.04, 0.06) |  |  |  |  |  |  |
| Australasia | 5 | 1990-2000 | -0.02(-0.02, -0.02) | 2000-2004 | -0.02(-0.02, -0.02) | 2004-2007 | -0.02(-0.03, -0.02) | 2007-2012 | -0.02(-0.02, -0.02) | 2012-2016 | -0.01(-0.01, -0.01) | 2016-2021 | -0.01(-0.01, -0.01) |
| Caribbean | 5 | 1990-1994 | 0.01(0.01, 0.01) | 1994-2001 | 0.01(0.01, 0.01) | 2001-2007 | 0(0, 0) | 2007-2014 | 0.01(0, 0.01) | 2014-2019 | 0.01(0.01, 0.01) | 2019-2021 | 0.03(0.02, 0.03) |
| Central Asia | 4 | 1990-1992 | -0.07(-0.08, -0.06) | 1992-2000 | -0.08(-0.09, -0.05) | 2000-2003 | -0.05(-0.06, -0.02) | 2003-2010 | -0.02(-0.02, 0) | 2010-2021 | 0(0, 0) |  |  |
| Central Europe | 4 | 1990-1993 | 0(0, 0.01) | 1993-2001 | -0.01(-0.01, -0.01) | 2001-2005 | 0(-0.01, 0) | 2005-2019 | -0.02(-0.02, -0.02) | 2019-2021 | 0(-0.01, 0) |  |  |
| Central Latin America | 2 | 1990-2005 | 0.01(0.01, 0.01) | 2005-2019 | 0.01(0.01, 0.01) | 2019-2021 | 0.04(0.04, 0.05) |  |  |  |  |  |  |
| Central Sub-Saharan Africa | 5 | 1990-1996 | 0.09(0.09, 0.1) | 1996-2001 | 0.07(0.06, 0.07) | 2001-2006 | 0.03(0.02, 0.03) | 2006-2015 | 0(-0.01, 0) | 2015-2019 | -0.03(-0.04, -0.03) | 2019-2021 | 0.01(-0.01, 0.02) |
| East Asia | 5 | 1990-1997 | -0.03(-0.03, -0.03) | 1997-2004 | -0.02(-0.02, -0.02) | 2004-2009 | -0.01(-0.01, -0.01) | 2009-2014 | 0(0, 0) | 2014-2018 | 0.02(0.02, 0.02) | 2018-2021 | 0.01(0, 0.01) |
| Eastern Europe | 4 | 1990-2000 | -0.07(-0.07, -0.07) | 2000-2003 | -0.03(-0.04, -0.03) | 2003-2009 | 0(-0.01, 0) | 2009-2017 | -0.02(-0.03, -0.02) | 2017-2021 | -0.01(-0.02, 0) |  |  |
| Eastern Sub-Saharan Africa | 4 | 1990-1996 | 0.02(0.02, 0.02) | 1996-2006 | 0.03(0.03, 0.03) | 2006-2011 | 0.02(0.01, 0.02) | 2011-2019 | 0.01(0.01, 0.01) | 2019-2021 | 0.05(0.04, 0.05) |  |  |
| High-income Asia Pacific | 5 | 1990-1993 | -0.01(-0.02, -0.01) | 1993-1998 | -0.01(-0.01, -0.01) | 1998-2001 | -0.02(-0.02, -0.02) | 2001-2007 | -0.02(-0.02, -0.02) | 2007-2010 | -0.02(-0.02, -0.02) | 2010-2021 | -0.02(-0.02, -0.02) |
| High-income North America | 5 | 1990-1994 | 0.68(0.61, 0.76) | 1994-2000 | -0.12(-0.18, -0.07) | 2000-2010 | -0.56(-0.59, -0.54) | 2010-2015 | 0.16(0.06, 0.2) | 2015-2018 | 0.68(0.53, 0.77) | 2018-2021 | 0.1(-0.06, 0.19) |
| North Africa and Middle East | 4 | 1990-2002 | 0(0, 0) | 2002-2008 | 0.01(0, 0.01) | 2008-2012 | 0(0, 0.01) | 2012-2019 | 0(0, 0) | 2019-2021 | 0.02(0.01, 0.02) |  |  |
| Oceania | 4 | 1990-1995 | 0.01(0.01, 0.02) | 1995-2010 | -0.01(-0.01, -0.01) | 2010-2013 | 0.02(0.01, 0.02) | 2013-2016 | -0.02(-0.03, -0.01) | 2016-2021 | 0.01(0.01, 0.02) |  |  |
| South Asia | 5 | 1990-1994 | 0(-0.01, 0) | 1994-1997 | 0.02(0.01, 0.03) | 1997-2004 | 0.05(0.05, 0.06) | 2004-2007 | 0.02(0.02, 0.03) | 2007-2019 | 0(0, 0) | 2019-2021 | 0.02(0.01, 0.02) |
| Southeast Asia | 5 | 1990-1994 | 0.01(0, 0.01) | 1994-2005 | 0.02(0.01, 0.02) | 2005-2009 | 0.01(0, 0.01) | 2009-2012 | 0(-0.01, 0) | 2012-2019 | -0.01(-0.02, -0.01) | 2019-2021 | 0(0, 0.01) |
| Southern Latin America | 5 | 1990-1992 | 0(0, 0.01) | 1992-1999 | 0.01(0, 0.01) | 1999-2004 | 0(-0.01, 0.01) | 2004-2012 | -0.01(-0.01, 0) | 2012-2019 | -0.01(-0.01, -0.01) | 2019-2021 | 0(-0.01, 0) |
| Southern Sub-Saharan Africa | 4 | 1990-1993 | 0.02(0, 0.04) | 1993-2001 | 0.06(0.05, 0.06) | 2001-2006 | 0(0, 0.02) | 2006-2019 | -0.02(-0.02, -0.02) | 2019-2021 | 0.04(0.02, 0.05) |  |  |
| Tropical Latin America | 5 | 1990-1995 | 0.02(0.02, 0.02) | 1995-2002 | 0.02(0.01, 0.02) | 2002-2013 | 0(0, 0.02) | 2013-2016 | -0.01(-0.01, 0) | 2016-2019 | 0(-0.01, 0.01) | 2019-2021 | 0.02(0.01, 0.02) |
| Western Europe | 5 | 1990-2000 | -0.02(-0.02, -0.02) | 2000-2003 | -0.02(-0.02, -0.02) | 2003-2009 | -0.03(-0.03, -0.02) | 2009-2015 | -0.02(-0.03, -0.02) | 2015-2019 | -0.02(-0.02, -0.02) | 2019-2021 | -0.01(-0.01, -0.01) |
| Western Sub-Saharan Africa | 5 | 1990-1996 | -0.07(-0.08, -0.07) | 1996-2000 | -0.05(-0.06, -0.03) | 2000-2004 | -0.01(-0.03, 0) | 2004-2013 | 0.02(0.02, 0.03) | 2013-2019 | 0.05(0.04, 0.05) | 2019-2021 | 0.08(0.06, 0.09) |

N represents the number of turning points in the Jointpoint regression analysis.

CI, confidence interval; APC, annual percentage change.

**Table S7 The prevalence and DALYs of oral diseases and their trends from 1990 to 2021 at the national level**

| **Location** | **Prevalence** | | | **DALYs** | | |
| --- | --- | --- | --- | --- | --- | --- |
|  | ASR in 1990 | ASR in 2021 | AAPC (1990-2021) | ASR in 1990 | ASR in 2021 | AAPC (1990-2021) |
| Afghanistan | 74528.6(66731.2-80968.9) | 73883.4(65118.9-81080.3) | -0.03(-0.03, -0.03) | 1259.2(801.3-1841.6) | 1191(756-1736.4) | -0.19(-0.19, -0.18) |
| Albania | 71851(64012.1-78945.1) | 68062.3(59338.5-76574.7) | -0.17(-0.18, -0.17) | 1374.7(882.2-1994.3) | 1203.6(761.1-1776.8) | -0.43(-0.43, -0.42) |
| Algeria | 71976.3(64455.5-78427.3) | 71093.3(62990.5-78531.1) | -0.04(-0.04, -0.04) | 1152.9(733.9-1697.7) | 1100.7(688.8-1648.1) | -0.15(-0.15, -0.15) |
| American Samoa | 69106.5(61409.4-75722) | 69891.8(61949.9-76621.3) | 0.04(0.03, 0.04) | 942.1(582.2-1427) | 944.7(586.5-1427) | 0.01(0, 0.01) |
| Andorra | 71453.2(63534-78132) | 69771.6(61205.5-77196.5) | -0.08(-0.08, -0.07) | 1031.8(646.9-1553.3) | 982.4(608.3-1466.8) | -0.16(-0.16, -0.15) |
| Angola | 63565.2(55685.6-70854.2) | 59055.2(50420.8-67551.4) | -0.24(-0.25, -0.23) | 695.5(418.8-1072.5) | 623.9(373.8-948.5) | -0.35(-0.36, -0.34) |
| Antigua and Barbuda | 68632.9(61244.1-75405.2) | 66066.5(57825.3-74161.5) | -0.12(-0.12, -0.12) | 1158.5(728.6-1722.6) | 1055.6(657.9-1587.7) | -0.3(-0.3, -0.3) |
| Argentina | 75373.4(67729.9-81764.2) | 74605.8(66710.6-81317.3) | -0.04(-0.04, -0.03) | 1161.2(721.9-1723) | 1051.3(658.1-1572.4) | -0.33(-0.33, -0.32) |
| Armenia | 70034(62516.9-77028.8) | 65998.4(57030-74424.3) | -0.18(-0.19, -0.18) | 1189.1(749.3-1764.2) | 1081.7(683.5-1608) | -0.32(-0.33, -0.3) |
| Australia | 73059.5(68804.3-77338.5) | 63652.3(55396.6-71586.8) | -0.44(-0.46, -0.42) | 1517(1024.1-2120.9) | 1130.8(715.7-1672.2) | -0.86(-0.98, -0.74) |
| Austria | 69438.3(61703.5-76297.6) | 67560.8(59081.7-75203.1) | -0.09(-0.12, -0.07) | 1211.9(761.8-1770.9) | 1111.1(698.5-1646.1) | -0.29(-0.35, -0.23) |
| Azerbaijan | 68720.2(61185.6-75775.4) | 65113.2(56045.2-73587.8) | -0.17(-0.18, -0.16) | 1141.9(710.6-1709.7) | 1047.1(654.8-1561.1) | -0.28(-0.3, -0.26) |
| Bahamas | 70098(62853.9-76720.5) | 68425.7(60269.4-75940.2) | -0.07(-0.08, -0.07) | 1121.2(698.6-1679.8) | 1056.8(653.5-1584.9) | -0.19(-0.19, -0.19) |
| Bahrain | 70531.6(63127.1-77043) | 69336.6(61124.1-76815.8) | -0.06(-0.06, -0.05) | 1076.2(674.5-1602.4) | 1019.3(643.1-1507.7) | -0.18(-0.18, -0.18) |
| Bangladesh | 65725(57152.5-72884.6) | 66243.2(58122.8-73989.8) | 0.02(0.01, 0.03) | 570.4(334.3-911.7) | 532.8(302-847.5) | -0.22(-0.23, -0.22) |
| Barbados | 68474.3(61710.3-75376.2) | 66944.6(59755.5-74369.7) | -0.07(-0.07, -0.07) | 1167.9(719.5-1652.8) | 1096.3(663.5-1553.3) | -0.2(-0.21, -0.2) |
| Belarus | 63572.5(56233.1-70581.1) | 62266.4(53718.6-70527.2) | -0.07(-0.08, -0.07) | 1056(665.2-1568.7) | 1004.8(625.2-1500.9) | -0.16(-0.17, -0.16) |
| Belgium | 70033.1(63170.1-76301.3) | 68061.3(60267.5-75253.5) | -0.11(-0.12, -0.09) | 1249.9(788.5-1853) | 1164.5(726.8-1728.4) | -0.22(-0.24, -0.2) |
| Belize | 70164.2(62737-77169.4) | 63688.8(55546.3-71591.6) | -0.3(-0.32, -0.3) | 1221.1(769.6-1805.3) | 1106.5(687.4-1654.2) | -0.32(-0.32, -0.31) |
| Benin | 65455.6(57702.1-72500.1) | 62090.7(54015.4-70048.4) | -0.18(-0.19, -0.17) | 682.1(408.9-1076.7) | 631(376.6-972.4) | -0.25(-0.26, -0.24) |
| Bermuda | 67396.5(60520.8-73902.7) | 65763(59014.6-72327.6) | -0.08(-0.08, -0.08) | 1114.2(697.8-1665.9) | 1026.5(643.8-1547.4) | -0.27(-0.27, -0.26) |
| Bhutan | 66605(58699.9-73680.4) | 66354.7(58310.9-74189.3) | -0.01(-0.01, -0.01) | 679.1(407.3-1058.5) | 618.9(357.9-967.8) | -0.3(-0.3, -0.3) |
| Bolivia (Plurinational State of) | 84451.3(78425.6-89300.5) | 84276(78202.7-89432.8) | -0.01(-0.01, 0) | 1815.3(1201.5-2580.1) | 1833.2(1173.5-2521.7) | 0.03(0.01, 0.05) |
| Bosnia and Herzegovina | 73174.3(65106.7-80384.5) | 68191.3(59184-76804.4) | -0.25(-0.3, -0.21) | 1437(926.8-2071.6) | 1203.5(762.4-1755.2) | -0.6(-0.72, -0.49) |
| Botswana | 52954.4(44711-61148.7) | 47944.5(39705.7-56645) | -0.33(-0.34, -0.31) | 718.2(446.5-1069.8) | 629.6(392.8-921.3) | -0.42(-0.43, -0.41) |
| Brazil | 78443.3(71878.8-84184.3) | 79032(73368.9-84342.4) | 0.02(0.01, 0.02) | 1694.2(1125.4-2396.6) | 1695.5(1094.9-2318.6) | -0.01(-0.02, 0.01) |
| Brunei Darussalam | 48210.1(41605.1-54392.2) | 45882.4(38662.8-53204.1) | -0.16(-0.17, -0.15) | 661.8(399.4-1016.6) | 645(395.8-966.4) | -0.09(-0.1, -0.09) |
| Bulgaria | 69247.7(60865.1-77194.1) | 66606.8(57320.6-75560.2) | -0.13(-0.14, -0.12) | 1191.7(751.9-1757.8) | 1072.3(674.1-1587.2) | -0.36(-0.4, -0.31) |
| Burkina Faso | 63789.2(55706.2-71208.5) | 63257.4(55244.2-70560) | -0.04(-0.05, -0.03) | 588.9(343.3-945.3) | 573.1(330-926.7) | -0.1(-0.11, -0.08) |
| Burundi | 67223.3(59097.3-74420.5) | 62042.5(52534.7-70833.2) | -0.25(-0.27, -0.24) | 589.1(344.7-948) | 521.5(311.4-808.5) | -0.39(-0.4, -0.38) |
| Cabo Verde | 65512.2(57800.6-72313.3) | 64616.3(57372.5-71405) | -0.05(-0.06, -0.05) | 696.3(414.7-1095.2) | 652.8(386.2-1039.5) | -0.21(-0.21, -0.21) |
| Cambodia | 74043.5(65287.4-81217.2) | 71960.7(63414.6-79511.8) | -0.09(-0.09, -0.09) | 1052.4(658.2-1565.8) | 955.9(590-1435.8) | -0.31(-0.32, -0.31) |
| Cameroon | 64871.7(57411.6-71689.6) | 64719.9(57156.4-71800.5) | -0.01(-0.02, -0.01) | 667.2(396.8-1053.5) | 655.6(385.8-1039.4) | -0.06(-0.06, -0.05) |
| Canada | 56287.9(50097-62148) | 54046.3(47321.9-60776.4) | -0.14(-0.15, -0.12) | 703.2(421.1-1082.9) | 672.1(408.7-1011.9) | -0.16(-0.2, -0.11) |
| Central African Republic | 64214.8(56145.3-71220.1) | 59892.2(51071-68388.3) | -0.22(-0.23, -0.21) | 717.6(435.4-1098.9) | 665.4(410.1-998.2) | -0.24(-0.25, -0.23) |
| Chad | 66308.2(58680.5-73037.3) | 62819.3(54469-70827.6) | -0.19(-0.2, -0.18) | 755.2(460.1-1172.7) | 685.7(417.9-1027) | -0.31(-0.33, -0.3) |
| Chile | 76733.5(69276.6-82612.5) | 77023.3(73422.9-80461.7) | 0.01(0, 0.02) | 1192.6(757-1756.5) | 1050.5(658.4-1569.6) | -0.42(-0.43, -0.41) |
| China | 58372(51711.3-64177) | 57707.9(52342.9-62840.4) | -0.04(-0.05, -0.03) | 1007.1(622-1509.5) | 951.2(597.7-1405.2) | -0.19(-0.23, -0.15) |
| Colombia | 68842.4(61819.5-75351.2) | 66989.2(58666.7-74858) | -0.12(-0.18, -0.08) | 1115.8(702.2-1665.7) | 1050.8(654-1567.9) | -0.2(-0.21, -0.19) |
| Comoros | 62438.1(55914.3-68098.2) | 51108.5(42615.8-59433.4) | -0.64(-0.66, -0.62) | 601.5(355.4-958.9) | 529.2(314.6-817.7) | -0.41(-0.42, -0.4) |
| Congo | 63333.8(55703.4-70345.9) | 59108.6(50473.9-67722) | -0.22(-0.24, -0.21) | 691.1(413.9-1069.7) | 629.1(380.8-952.8) | -0.3(-0.31, -0.29) |
| Cook Islands | 69492.9(61849.2-76279) | 59973.4(50166-69188.7) | -0.47(-0.49, -0.45) | 965.9(595.8-1467.9) | 779.6(484-1157.9) | -0.68(-0.7, -0.66) |
| Costa Rica | 72472.2(65090.2-79123.2) | 70651.8(62613.1-78174.3) | -0.08(-0.09, -0.08) | 1168.5(735.5-1728.5) | 1090.7(681.3-1622.3) | -0.22(-0.22, -0.22) |
| Coted'Ivoire | 65567.5(58394.7-72103.2) | 62640.6(54429.1-70266.2) | -0.15(-0.16, -0.15) | 743.6(445.4-1151.9) | 704.3(427.2-1071.4) | -0.18(-0.2, -0.16) |
| Croatia | 72380.2(65638.3-78934.7) | 69292.7(61190.4-76971.1) | -0.15(-0.15, -0.14) | 1237.3(780.9-1814.5) | 1099.5(691-1648.7) | -0.37(-0.43, -0.31) |
| Cuba | 69662.5(62235.3-76436.2) | 66514.2(58928.5-73656.9) | -0.15(-0.15, -0.14) | 1218.7(766.2-1803.7) | 1153.7(723.2-1723.9) | -0.17(-0.18, -0.15) |
| Cyprus | 73026.3(65034.5-79508.1) | 70750.2(62219.6-78385.9) | -0.1(-0.11, -0.1) | 1109.2(692.8-1648.2) | 1037.1(651.2-1551.7) | -0.22(-0.22, -0.22) |
| Czechia | 69425.6(61835-76488.6) | 66817.9(58022.6-75122.1) | -0.13(-0.14, -0.12) | 1259.8(801.6-1829.1) | 1132.8(708.5-1698.1) | -0.36(-0.39, -0.33) |
| Democratic People's Republic of Korea | 50155.3(43479.2-56741.6) | 51824.4(44676-58555.5) | 0.1(0.1, 0.11) | 875.4(532.1-1324.7) | 973.9(597.1-1452.1) | 0.35(0.34, 0.35) |
| Democratic Republic of the Congo | 63854(55943.7-71080.5) | 59991(51124-68742.4) | -0.2(-0.21, -0.19) | 708.5(429-1089.5) | 667.3(409.6-1010.8) | -0.19(-0.2, -0.18) |
| Denmark | 65404.1(59579.5-70739.7) | 62630.8(55829.9-68786.2) | -0.14(-0.15, -0.13) | 1102.6(682.5-1640.3) | 1037.1(643.4-1569.2) | -0.2(-0.26, -0.14) |
| Djibouti | 61895.3(55688.2-67738.2) | 50640(41960.5-59098.4) | -0.65(-0.68, -0.64) | 565.3(324.8-911.5) | 492.4(287.4-762.9) | -0.44(-0.46, -0.43) |
| Dominica | 69803.9(62410.2-76506.7) | 67186.9(58936.6-75176.2) | -0.12(-0.12, -0.11) | 1226.1(773.4-1820.5) | 1112.4(702.1-1660.5) | -0.31(-0.31, -0.3) |
| Dominican Republic | 71164.2(64070.3-77975.1) | 67382.2(58737.2-75710.4) | -0.18(-0.18, -0.17) | 1312.6(834.9-1932.6) | 1168.4(737.2-1734.8) | -0.38(-0.4, -0.37) |
| Ecuador | 81977.5(75112.1-87853.7) | 80151.1(72140.4-86587.9) | -0.07(-0.09, -0.06) | 1685.5(1103.4-2412.9) | 1558.9(1006.8-2249.6) | -0.25(-0.29, -0.2) |
| Egypt | 69151.5(61355.7-76160.5) | 67090.4(58550.8-75219.3) | -0.1(-0.1, -0.1) | 1196.7(756.3-1755.4) | 1104.8(692-1653.5) | -0.26(-0.26, -0.25) |
| El Salvador | 73490.7(65567.1-80197.5) | 71445.3(62853-79137.4) | -0.09(-0.09, -0.09) | 1212.4(766.6-1784.1) | 1144.4(717.5-1706.3) | -0.19(-0.19, -0.18) |
| Equatorial Guinea | 64015.7(56257.9-71204.9) | 58216.2(50193.4-66433.8) | -0.3(-0.31, -0.3) | 704.8(424.5-1085.9) | 595.7(356.9-910.6) | -0.54(-0.55, -0.53) |
| Eritrea | 70699.2(62467.9-77714.4) | 65833.4(56727.2-74256.9) | -0.25(-0.27, -0.24) | 596.3(349.6-953) | 521.6(307-802.2) | -0.44(-0.46, -0.42) |
| Estonia | 58081.6(51715.5-64393.8) | 55622.4(48177.8-62994.7) | -0.14(-0.16, -0.13) | 1068.6(668.3-1591.8) | 979.4(607.4-1459.2) | -0.29(-0.34, -0.25) |
| Eswatini | 51245.6(42953.9-59313.6) | 51187.3(42863.8-59539.8) | 0(0, 0.01) | 638(394-959.4) | 608.4(373.7-919.5) | -0.15(-0.17, -0.14) |
| Ethiopia | 61356.9(54875.5-67473.1) | 59346.4(52919.6-65388.3) | -0.11(-0.11, -0.11) | 575.9(341-915.6) | 558.1(327.7-890.7) | -0.11(-0.12, -0.09) |
| Fiji | 70710.8(63163.5-77174.1) | 62086.3(52089.9-71147.3) | -0.43(-0.44, -0.41) | 1012.8(625.3-1520.9) | 834.3(520.2-1229.3) | -0.62(-0.64, -0.61) |
| Finland | 70460.4(63767.6-76209.3) | 64233.8(56362.6-70925) | -0.29(-0.32, -0.25) | 1477.2(947.3-2124.3) | 1106.4(690.1-1665.6) | -0.89(-0.98, -0.8) |
| France | 74615.4(66259-81326) | 73677.9(64633.8-80971.6) | -0.04(-0.06, -0.03) | 983.1(617.7-1457.5) | 930.4(585.2-1400.9) | -0.14(-0.24, -0.04) |
| Gabon | 62286.4(55002.4-69251.7) | 58493.6(50083.6-67046.8) | -0.2(-0.21, -0.2) | 659.4(392.3-1024.7) | 606.1(366.4-916.8) | -0.27(-0.28, -0.26) |
| Gambia | 66521.4(59546.4-73001.4) | 66555(59557.1-73183.5) | 0(-0.01, 0.01) | 695.2(410.1-1092.3) | 683.6(402.4-1088.6) | -0.05(-0.06, -0.05) |
| Georgia | 69065.7(61667.9-75772.9) | 66861.6(57875.1-75213.6) | -0.11(-0.12, -0.11) | 1170.8(733.9-1745.4) | 1100.2(694.7-1636.8) | -0.2(-0.21, -0.19) |
| Germany | 72044.5(64804.3-77879.5) | 64946.3(57801.5-71875.1) | -0.32(-0.34, -0.31) | 1127.9(708.1-1688.7) | 1031.5(637.9-1578.1) | -0.31(-0.37, -0.26) |
| Ghana | 62093.2(54889.3-69106.1) | 61463.1(54127.7-68268.8) | -0.04(-0.04, -0.03) | 678.4(406.8-1072.8) | 651.1(384.2-1029.1) | -0.14(-0.16, -0.12) |
| Greece | 75169.6(67708.8-81251.3) | 74345.7(66764.4-81291.5) | -0.04(-0.06, -0.03) | 1169.2(741.3-1720) | 1128.8(686.2-1586.8) | -0.15(-0.22, -0.09) |
| Greenland | 54980.7(48456.4-61245.9) | 53107.9(46145-60212.9) | -0.11(-0.12, -0.11) | 790.9(485.7-1199.5) | 732.1(449.1-1095.9) | -0.25(-0.25, -0.24) |
| Grenada | 70081.7(62758.8-77156) | 67085.7(58678.5-75302.8) | -0.14(-0.15, -0.14) | 1225.7(771.1-1822.2) | 1099(687.7-1641.1) | -0.35(-0.36, -0.35) |
| Guam | 67953.3(60519.9-74621.3) | 58725.7(48971-68030) | -0.48(-0.5, -0.46) | 903.8(548.3-1392.9) | 760.4(468.4-1137.9) | -0.56(-0.57, -0.54) |
| Guatemala | 73398.4(65621.2-79969.1) | 71362.7(62865.8-79121.4) | -0.09(-0.09, -0.08) | 1208.7(767.2-1775.4) | 1137.8(720.3-1682.3) | -0.2(-0.22, -0.19) |
| Guinea | 65379(57750-72398.4) | 64792.5(57056.5-71962.8) | -0.03(-0.04, -0.03) | 683.6(405.3-1074.4) | 662.9(393.1-1051.1) | -0.1(-0.1, -0.1) |
| Guinea-Bissau | 70632.3(62942.8-77192) | 67830(59545.2-75503.3) | -0.14(-0.15, -0.14) | 694.9(415.3-1088.1) | 649(391.3-992.1) | -0.22(-0.23, -0.22) |
| Guyana | 70610.9(63344.8-77617.3) | 67274.7(58584.6-75441) | -0.15(-0.16, -0.15) | 1230.4(774.8-1822.5) | 1103.1(688.7-1648.5) | -0.35(-0.36, -0.35) |
| Haiti | 71592.9(64063.4-78679.5) | 66683.8(58293-74579.1) | -0.23(-0.23, -0.22) | 1272.6(802.5-1859) | 1209.4(757.9-1770.5) | -0.17(-0.17, -0.16) |
| Honduras | 73570.2(65817.2-80080.6) | 72148.5(63596.8-79864.9) | -0.06(-0.07, -0.06) | 1214.6(773.6-1790.6) | 1149.6(722.1-1711.2) | -0.18(-0.18, -0.18) |
| Hungary | 64669(55922.6-73303.9) | 61259.2(52014.8-70403.9) | -0.18(-0.18, -0.17) | 1230(796.8-1762.1) | 1102.6(697.8-1606.1) | -0.37(-0.39, -0.34) |
| Iceland | 72833.8(67082.5-78089.4) | 64960.7(57404.2-72432.4) | -0.36(-0.37, -0.35) | 1081.9(675.1-1614.5) | 1010.9(630.9-1509) | -0.22(-0.22, -0.21) |
| India | 72035.1(65174-77516.9) | 69767.5(63956.1-75143.1) | -0.11(-0.14, -0.09) | 887.6(550-1334.5) | 806.8(495.6-1161.9) | -0.36(-0.47, -0.26) |
| Indonesia | 70728(63035.1-77616.5) | 69619.1(62197.2-76465.7) | -0.05(-0.06, -0.05) | 1023.3(638.8-1537.2) | 979.8(607.5-1411.5) | -0.13(-0.14, -0.13) |
| Iran (Islamic Republic of) | 70183.6(64296.3-75399.8) | 70068.6(63844.4-75946.9) | -0.01(-0.01, 0) | 1073.7(674.4-1598.9) | 1085.9(660.7-1545) | 0.04(0, 0.07) |
| Iraq | 71961.6(64139.9-78703.1) | 70586(61989.6-78194.2) | -0.06(-0.07, -0.06) | 1141.7(716-1685.5) | 1056(661.2-1565.1) | -0.25(-0.25, -0.25) |
| Ireland | 73963.5(65815.3-80721.4) | 70649.6(61455.7-78366.7) | -0.17(-0.19, -0.15) | 1293(830.6-1879) | 1151.9(723.8-1682.4) | -0.39(-0.44, -0.34) |
| Israel | 77469.3(69461-83581.8) | 75232.1(66843.9-82189.7) | -0.1(-0.11, -0.08) | 1130.8(712.3-1681.1) | 1047.3(661.9-1564.6) | -0.26(-0.28, -0.24) |
| Italy | 66742.2(59142-73167.8) | 65266.1(57332.1-72376.1) | -0.08(-0.09, -0.07) | 996.8(624.7-1477.1) | 971.1(606.2-1452.7) | -0.06(-0.11, -0.01) |
| Jamaica | 69604.5(62295.8-76364) | 67431.2(58881.5-75328.5) | -0.1(-0.1, -0.1) | 1214.9(764.7-1807.6) | 1126.6(703.3-1681.6) | -0.24(-0.24, -0.24) |
| Japan | 49202.7(42430.1-55431.7) | 44847.4(38520.9-51182.9) | -0.23(-0.31, -0.16) | 924.2(572.1-1393) | 775.8(482.9-1144) | -0.45(-0.64, -0.25) |
| Jordan | 72825(65203.9-79414.2) | 71348.2(63310.9-78718.2) | -0.07(-0.07, -0.06) | 1180.4(750.5-1744.9) | 1113.2(701.9-1652.6) | -0.2(-0.2, -0.19) |
| Kazakhstan | 70648.1(63079-77645.4) | 66914.3(58076.4-75567) | -0.18(-0.19, -0.17) | 1271.6(808.4-1881.5) | 1148.7(724.7-1696.8) | -0.34(-0.35, -0.33) |
| Kenya | 61340.7(54410.3-67547) | 59193.6(52384.6-65782) | -0.11(-0.12, -0.1) | 589(346.2-938.8) | 577.7(339.1-884.6) | -0.06(-0.07, -0.05) |
| Kiribati | 71682.4(64006.2-78287.2) | 65595.6(56057.8-74095.2) | -0.3(-0.31, -0.29) | 1057(655.5-1569.7) | 934.8(585.1-1363.5) | -0.4(-0.41, -0.39) |
| Kuwait | 70302.3(62992-77016.9) | 69381.8(61179.1-76969.1) | -0.04(-0.05, -0.04) | 1084.6(679.3-1609.9) | 1031.8(642.9-1543.5) | -0.16(-0.17, -0.15) |
| Kyrgyzstan | 69676.1(62012.1-76684.5) | 67390.9(58362.9-75882.5) | -0.11(-0.12, -0.1) | 1174.3(739.2-1741.6) | 1128.4(708.6-1672.5) | -0.13(-0.15, -0.11) |
| Lao People's Democratic Republic | 58800.3(49484.6-67791.5) | 56138(47087.7-65325.9) | -0.15(-0.17, -0.14) | 745.1(461.4-1108.1) | 649.8(403-981.9) | -0.47(-0.5, -0.43) |
| Latvia | 62602(55551.3-69610.2) | 60921.9(52939.6-69025.6) | -0.09(-0.09, -0.08) | 976.4(608.5-1477.4) | 917.4(569.5-1374.5) | -0.21(-0.25, -0.17) |
| Lebanon | 72110.1(64555.1-78746.6) | 70843.4(62434.3-78402.9) | -0.06(-0.06, -0.06) | 1142.8(726.7-1690) | 1075.1(678.1-1603.7) | -0.2(-0.2, -0.19) |
| Lesotho | 54021.4(45781.9-61891.3) | 53343.6(45303.1-61189.6) | -0.04(-0.05, -0.03) | 769.7(482-1127.1) | 722.1(447.6-1062.8) | -0.2(-0.21, -0.2) |
| Liberia | 65389.4(57694-72190.3) | 65279.2(57594.4-72345.8) | -0.01(-0.01, 0) | 680.2(404.3-1065.9) | 673.8(403.4-1060.2) | -0.03(-0.04, -0.03) |
| Libya | 70103.9(62993.8-76662.9) | 71934.9(63468.4-79499.6) | 0.08(0.08, 0.09) | 1080.9(677.3-1602.4) | 1118.1(706.4-1651.1) | 0.11(0.1, 0.11) |
| Lithuania | 65670.1(57910.8-72762.4) | 63818.7(55531-72029.5) | -0.09(-0.1, -0.09) | 951(592-1448.1) | 884.2(545.4-1323.8) | -0.23(-0.26, -0.19) |
| Luxembourg | 72605.9(64983.7-78949.1) | 69786(61278.9-77530.7) | -0.13(-0.14, -0.13) | 1135(712.9-1687.1) | 1032.3(648.4-1536.2) | -0.32(-0.34, -0.3) |
| Madagascar | 76596.8(68332.5-83117.5) | 68284.4(58250.8-76844.5) | -0.4(-0.42, -0.38) | 594.4(345.2-957.3) | 444.1(271.9-669) | -0.94(-0.98, -0.9) |
| Malawi | 67645.6(59354.3-74942.9) | 62045(52800.6-71006.7) | -0.28(-0.29, -0.27) | 597.9(349.2-959.3) | 523.4(310.9-810.3) | -0.43(-0.44, -0.42) |
| Malaysia | 66536.8(59468.4-72702.1) | 61809.5(53715.3-69848.2) | -0.27(-0.31, -0.22) | 1233.2(782.7-1816.4) | 1072.7(669.4-1595.7) | -0.43(-0.52, -0.33) |
| Maldives | 67009.9(58760.4-74622.5) | 63095.3(54496.9-71815.9) | -0.2(-0.2, -0.19) | 933.7(574.7-1404.6) | 832.7(511.5-1242.9) | -0.37(-0.37, -0.37) |
| Mali | 65131.9(57154.2-72243) | 64353.6(56208.4-71540.6) | -0.05(-0.05, -0.04) | 616.7(363.9-970.7) | 592.2(345.5-949.8) | -0.14(-0.16, -0.12) |
| Malta | 71411.6(62974.4-78217.5) | 68424.3(59655.7-75845.4) | -0.14(-0.14, -0.14) | 968.3(604.4-1446) | 870.1(543.8-1299.4) | -0.35(-0.36, -0.34) |
| Marshall Islands | 71386.8(63480.3-77930.7) | 64539.2(54675.3-73425.3) | -0.32(-0.34, -0.31) | 1051.3(654.9-1575.4) | 917.8(575-1353.7) | -0.45(-0.46, -0.43) |
| Mauritania | 65765.1(58286.2-72274.9) | 62466(54705.8-70566.5) | -0.18(-0.19, -0.18) | 756.8(455.2-1173.6) | 700.6(420.5-1058.3) | -0.26(-0.28, -0.24) |
| Mauritius | 71276(63943.1-77939.6) | 66937.1(58359.6-75224.4) | -0.21(-0.24, -0.19) | 1263.3(796.1-1840.9) | 1107.3(694.1-1646.1) | -0.44(-0.49, -0.39) |
| Mexico | 66129(59629.3-72256.8) | 65521.9(59044.6-72394.8) | -0.04(-0.06, -0.03) | 1212(771.6-1772.8) | 1203.6(742.3-1686.6) | -0.04(-0.09, 0) |
| Micronesia (Federated States of) | 71563.5(63716.4-78239.3) | 64237(54667.3-72874.4) | -0.35(-0.36, -0.33) | 1047.3(652.6-1567.5) | 906.4(572.1-1341.2) | -0.47(-0.49, -0.45) |
| Monaco | 70041.4(62300.7-76731.7) | 68345.4(60791.5-75941.9) | -0.08(-0.08, -0.08) | 967.8(602.4-1456.8) | 928.6(560.5-1332.8) | -0.13(-0.13, -0.12) |
| Mongolia | 70286.1(62566.6-77434.8) | 65339.7(56289.5-73776.9) | -0.24(-0.24, -0.23) | 1217.7(769.9-1801.9) | 1046.4(660-1567.9) | -0.49(-0.5, -0.48) |
| Montenegro | 70920.8(63331.3-77774.9) | 68790.2(60166.2-76980.8) | -0.1(-0.1, -0.1) | 1297.9(825.2-1903.8) | 1181.3(742.8-1749.7) | -0.31(-0.32, -0.3) |
| Morocco | 69824.3(64501.9-74704) | 60209.2(52640.3-67636.8) | -0.47(-0.49, -0.45) | 1313.8(833.1-1934.4) | 1176.9(732.4-1751.3) | -0.35(-0.36, -0.35) |
| Mozambique | 69688.1(61036.3-77231.7) | 62578.9(53088.1-71350.5) | -0.34(-0.35, -0.33) | 595.6(351.9-945.1) | 516.3(307.5-788.7) | -0.48(-0.49, -0.46) |
| Myanmar | 58254.6(50439.5-66226.1) | 53241.9(44977.2-61913.5) | -0.29(-0.31, -0.28) | 780.2(480.1-1176.5) | 656.8(405.5-986.9) | -0.56(-0.61, -0.51) |
| Namibia | 58055.7(49722.8-65789.9) | 57282.4(49203.5-64984) | -0.04(-0.06, -0.02) | 811.3(505.8-1191.6) | 773(477.9-1143.3) | -0.16(-0.18, -0.14) |
| Nauru | 69289.6(61605.6-75898.4) | 66331.1(57377.9-75031.3) | -0.13(-0.14, -0.11) | 954.8(583.2-1444.7) | 1081.3(657.7-1516.7) | 0.4(0.37, 0.44) |
| Nepal | 60447.1(51429.7-68483.6) | 58721.3(49671.7-67589.6) | -0.1(-0.11, -0.09) | 538.8(323.8-840.8) | 480.4(280.1-754.4) | -0.37(-0.38, -0.35) |
| Netherlands | 70507(64494.7-75948.6) | 63042.4(55144.5-70692.1) | -0.35(-0.38, -0.33) | 1404.5(896.7-2032.1) | 1298.9(821.3-1886.8) | -0.23(-0.29, -0.18) |
| New Zealand | 67527.5(60108-74712.2) | 64616.8(56106.9-72838) | -0.13(-0.15, -0.12) | 1370.6(879.6-1989.1) | 1212.6(757.5-1800.5) | -0.37(-0.4, -0.34) |
| Nicaragua | 73810.3(66310.6-80298.8) | 72365.8(64011-80169.6) | -0.06(-0.08, -0.05) | 1225.6(780.2-1796.5) | 1163.1(736.2-1741.4) | -0.17(-0.17, -0.17) |
| Niger | 67170(59477.7-74151.8) | 57728.7(48336.6-66938.6) | -0.49(-0.52, -0.47) | 696.6(418.6-1095.5) | 572.6(352.7-866) | -0.63(-0.66, -0.6) |
| Nigeria | 54922.4(48552-60671.7) | 38194(32248.5-44743.1) | -1.12(-1.16, -1.09) | 610.6(363.4-966.8) | 454.3(278.5-660.9) | -0.96(-1.02, -0.91) |
| Niue | 70127.5(62260.9-76925.3) | 61547.4(51612.5-70545.8) | -0.43(-0.44, -0.41) | 994.6(611.4-1503.7) | 827(516.2-1216.3) | -0.6(-0.62, -0.59) |
| North Macedonia | 71385.8(63650.7-78252.1) | 69600(62060-77006.4) | -0.08(-0.08, -0.08) | 1342.4(859.3-1958.5) | 1250.9(774.6-1743.1) | -0.23(-0.24, -0.22) |
| Northern Mariana Islands | 68366.6(60824.8-75118.1) | 59880(49998.9-69093.6) | -0.42(-0.44, -0.4) | 910.3(556.3-1379.6) | 789.5(492-1163.6) | -0.47(-0.49, -0.45) |
| Norway | 71633.2(65161.7-77308.8) | 70167.2(63689.8-76174.5) | -0.12(-0.19, -0.06) | 1153.9(725.3-1713.1) | 1115.7(706.7-1582) | -0.16(-0.32, -0.02) |
| Oman | 72143.2(64644.9-78686.3) | 70914.7(62645.6-78095.4) | -0.06(-0.06, -0.05) | 1069.2(677.3-1581.1) | 1010.7(631-1501.6) | -0.18(-0.19, -0.18) |
| Pakistan | 64864.1(57863-71030.4) | 66964.9(59479-74000.9) | 0.1(0.09, 0.11) | 838(511.5-1276.1) | 796.8(484-1203.8) | -0.18(-0.22, -0.13) |
| Palau | 69645.5(61802.5-76256.6) | 66000.2(57251.9-75152.2) | -0.18(-0.2, -0.17) | 971(598.3-1462.9) | 1052.9(645.9-1490.1) | 0.25(0.21, 0.28) |
| Palestine | 69971.2(62367.5-77034.8) | 68151.5(59744.2-76423.9) | -0.09(-0.09, -0.08) | 1218.6(776.4-1784.3) | 1138.7(715.3-1685) | -0.22(-0.22, -0.22) |
| Panama | 78462.4(71085.6-84493.7) | 76476.9(68588-83509.7) | -0.08(-0.08, -0.08) | 1174.7(740.9-1743.6) | 1076(669.8-1597.7) | -0.29(-0.29, -0.28) |
| Papua New Guinea | 71574.9(63789.9-77985.7) | 64011(54418.6-72495.3) | -0.36(-0.37, -0.34) | 1051.4(657.2-1575.8) | 906.8(570.9-1328.8) | -0.47(-0.49, -0.46) |
| Paraguay | 75378.7(68022.8-81610.2) | 72305(63549.1-79746.1) | -0.14(-0.15, -0.12) | 1314.2(839.4-1921) | 1169.1(738.5-1741.4) | -0.4(-0.46, -0.35) |
| Peru | 84530.8(78249.1-89526.9) | 82292.1(75014.7-88075.1) | -0.09(-0.09, -0.08) | 1861.2(1236.6-2639.3) | 1748.5(1147.2-2487.4) | -0.2(-0.21, -0.19) |
| Philippines | 73550.5(67032.9-79411.2) | 69447.9(63297.7-75269.9) | -0.18(-0.19, -0.18) | 1234.1(783.5-1812.5) | 1169.5(731.1-1622.7) | -0.17(-0.18, -0.15) |
| Poland | 70901.5(64927.1-76736.3) | 67896.2(60245.3-75347.4) | -0.13(-0.16, -0.1) | 1466.3(953.7-2109.5) | 1350.2(859.8-1975.1) | -0.21(-0.34, -0.1) |
| Portugal | 68767.3(60650.8-75828.7) | 65980.7(56777.2-74507.5) | -0.14(-0.15, -0.13) | 1114(699.7-1639.2) | 1027.5(640.7-1547.9) | -0.25(-0.26, -0.23) |
| Puerto Rico | 67882.2(60875.5-74833.5) | 65976.5(57971-73670.1) | -0.09(-0.11, -0.08) | 1135.2(716.7-1698.9) | 1049.7(650.7-1575.4) | -0.23(-0.28, -0.18) |
| Qatar | 66862.5(59254.7-74038.2) | 64398.3(56134.7-72548.9) | -0.13(-0.13, -0.12) | 807.6(498.1-1232.1) | 730.4(448.4-1093.2) | -0.33(-0.35, -0.31) |
| Republic of Korea | 50674(42927.4-58233.6) | 48470.3(40881.2-56341) | -0.13(-0.16, -0.11) | 702.1(429.4-1068.5) | 634.9(387.8-959.6) | -0.3(-0.4, -0.21) |
| Republic of Moldova | 64659.4(57302.7-71883.3) | 63557.2(55135.6-72183.1) | -0.06(-0.06, -0.06) | 1093.1(688.8-1623.8) | 1064.5(671.4-1582.5) | -0.08(-0.09, -0.08) |
| Romania | 70986.4(63069.6-78159.8) | 68407.6(59863.1-76734.4) | -0.12(-0.14, -0.11) | 1205.9(759.2-1780.3) | 1065.5(663.3-1596) | -0.42(-0.47, -0.37) |
| Russian Federation | 68394.3(61560.2-74790.8) | 69177.7(62735.4-75814.8) | 0.04(0.03, 0.04) | 1206(763.7-1774.3) | 1272(792.7-1776.2) | 0.17(0.15, 0.18) |
| Rwanda | 67545.2(59102.2-75188.2) | 61681.9(52293.6-70842.7) | -0.29(-0.3, -0.27) | 590.9(343.4-945.2) | 517.4(304-799.9) | -0.43(-0.44, -0.41) |
| Saint Kitts and Nevis | 68747.5(61661.2-75280.9) | 65929.4(58021.3-73756.7) | -0.13(-0.14, -0.13) | 1162.9(733.5-1721.9) | 1053.3(650.8-1578.6) | -0.32(-0.32, -0.32) |
| Saint Lucia | 69767.7(62406.8-76792.2) | 67173.1(60081.8-74605.9) | -0.12(-0.13, -0.12) | 1199.1(760.8-1779) | 1101(674-1557.5) | -0.28(-0.28, -0.27) |
| Saint Vincent and the Grenadines | 73737.2(66081.4-80346.6) | 70981.3(62561.1-78954.5) | -0.12(-0.12, -0.12) | 1234.5(780.2-1832.8) | 1098.6(686.6-1643.3) | -0.38(-0.38, -0.37) |
| Samoa | 71102.5(63240.1-77522.5) | 63404(53737-71986) | -0.38(-0.39, -0.37) | 1032.7(640.7-1549.3) | 878.4(543.4-1298.8) | -0.54(-0.55, -0.52) |
| San Marino | 71834.8(63838.9-78444.5) | 70205(61559.9-77942.4) | -0.08(-0.08, -0.07) | 1058.3(654.9-1580.2) | 1011.2(637-1521.1) | -0.15(-0.15, -0.14) |
| Sao Tome and Principe | 65270.6(57753.3-71994.1) | 62101.8(53893.3-70137.8) | -0.16(-0.18, -0.15) | 687.8(407.5-1079.6) | 629.3(373.4-956.2) | -0.29(-0.3, -0.28) |
| Saudi Arabia | 73618.5(65502.6-80953.4) | 73186.7(64184.3-81073.4) | -0.03(-0.04, -0.02) | 1028.4(650.2-1528) | 999.4(625-1487.1) | -0.1(-0.1, -0.09) |
| Senegal | 62043.4(55993.1-67380.2) | 53827.7(46822.6-60739.5) | -0.44(-0.46, -0.43) | 748.1(450.2-1161.9) | 685(418-1034.5) | -0.28(-0.29, -0.27) |
| Serbia | 69568.1(61786.3-76309.7) | 66881.2(58279.3-75178.7) | -0.14(-0.17, -0.12) | 1293.5(820.2-1902.4) | 1179.2(743.7-1736.6) | -0.31(-0.32, -0.3) |
| Seychelles | 66505.9(58836.6-73676.8) | 62903.6(54180.4-71491.3) | -0.18(-0.18, -0.17) | 932.8(577.3-1420) | 820.3(507.5-1224.1) | -0.41(-0.42, -0.41) |
| Sierra Leone | 58594.3(50983.1-65373.8) | 61974.2(55249.3-68473.7) | 0.19(0.16, 0.23) | 618.3(373.8-949) | 685(404-1060.4) | 0.36(0.33, 0.38) |
| Singapore | 47524.8(40289.4-54407.6) | 45836.3(38691.2-52945.2) | -0.12(-0.13, -0.12) | 705.8(429.7-1088.4) | 653.3(397.9-993.4) | -0.25(-0.25, -0.25) |
| Slovakia | 70828.4(63082.1-77940.1) | 67769.1(59150.9-76156.5) | -0.15(-0.16, -0.14) | 1343.7(864-1956.6) | 1203.4(755.5-1780.8) | -0.37(-0.38, -0.35) |
| Slovenia | 70362.7(63446.7-77205.4) | 66969.4(58605.4-75061.6) | -0.16(-0.18, -0.15) | 1325.4(847.7-1921.5) | 1175.4(741.4-1752.7) | -0.4(-0.44, -0.36) |
| Solomon Islands | 72209(64229.3-78692.9) | 65346.8(55678.5-73724.6) | -0.3(-0.32, -0.29) | 1089.1(673.8-1617.5) | 946.6(593.1-1387.6) | -0.45(-0.46, -0.44) |
| Somalia | 68297.4(59547.3-75635.5) | 62859.4(53222.4-71923) | -0.26(-0.27, -0.25) | 607.2(357.5-958.6) | 543.3(335.4-838.7) | -0.36(-0.38, -0.35) |
| South Africa | 58315.4(51297.4-64864.6) | 53589.4(46471.4-60782.4) | -0.26(-0.28, -0.24) | 872.2(542.3-1303.5) | 776.8(488.8-1144.6) | -0.35(-0.41, -0.3) |
| South Sudan | 66429.9(58110.1-73777.5) | 61763.4(52081.8-70880.9) | -0.23(-0.24, -0.22) | 553(317.3-898.7) | 498.5(296.2-767.2) | -0.35(-0.36, -0.33) |
| Spain | 75032.4(67537-81403.5) | 64742.4(55008.5-73456) | -0.47(-0.51, -0.45) | 1202.6(753.6-1763.4) | 816.8(513-1204.6) | -1.2(-1.28, -1.13) |
| Sri Lanka | 70162.9(62963-76713.4) | 62651.9(54009.9-70923.1) | -0.36(-0.38, -0.35) | 949.3(585.9-1431.8) | 778.9(485.9-1172.4) | -0.61(-0.66, -0.54) |
| Sudan | 74373.9(66655.1-80847.4) | 73507.3(65809-79872.6) | -0.04(-0.04, -0.04) | 1259.9(809.8-1851.9) | 1199.9(759.3-1762.3) | -0.16(-0.17, -0.16) |
| Suriname | 63255.2(56679.2-69674.4) | 60948.8(52923.3-68877.6) | -0.12(-0.12, -0.11) | 1158.1(722.3-1718.5) | 1078.3(674.2-1613.9) | -0.23(-0.23, -0.22) |
| Sweden | 62102.5(53988.8-69503.4) | 67103.2(59556.4-74878.4) | 0.26(0.24, 0.28) | 823.6(500.5-1263.6) | 936.4(547-1377.2) | 0.39(0.34, 0.43) |
| Switzerland | 79462.6(73208.8-84517.8) | 78412.2(71291.4-84084.2) | -0.05(-0.05, -0.04) | 1051.5(678.4-1518.2) | 966.3(602.4-1452.4) | -0.27(-0.29, -0.26) |
| Syrian Arab Republic | 73723.6(66323.9-80242) | 72788.3(64299.2-80098.4) | -0.04(-0.05, -0.04) | 1232.4(783.6-1811.1) | 1178(742.8-1746.3) | -0.15(-0.15, -0.14) |
| Taiwan (Province of China) | 50840.9(44605.8-56509.3) | 51150.8(44736.6-57061) | 0.02(0.02, 0.03) | 782.9(477.7-1209.4) | 803.1(488.2-1233.2) | 0.08(0.08, 0.09) |
| Tajikistan | 70036.8(62264.2-77138.3) | 68704.3(59828-77208.2) | -0.07(-0.07, -0.06) | 1195.2(755.8-1774.9) | 1183.8(749.2-1752.1) | -0.03(-0.04, -0.02) |
| Thailand | 67398.1(59278.3-74751.2) | 66165.5(58160.6-74323.2) | -0.06(-0.06, -0.06) | 970.9(602.1-1446.6) | 883.4(546.2-1332.1) | -0.31(-0.32, -0.3) |
| Timor-Leste | 72496.5(64323.8-79343) | 68716(60014.9-76843.7) | -0.16(-0.17, -0.16) | 986.6(620.8-1487) | 880.3(546.3-1315.7) | -0.37(-0.38, -0.37) |
| Togo | 65530.1(57667.4-72507.3) | 53197(45535-60684.6) | -0.65(-0.68, -0.63) | 688.5(411.6-1083.4) | 629.2(382.7-961.3) | -0.29(-0.3, -0.28) |
| Tokelau | 71028.7(63292.6-77621.3) | 67595.1(59041.2-76107.3) | -0.15(-0.18, -0.11) | 1036.3(645.1-1561.8) | 1118.2(681.4-1576) | 0.3(0.23, 0.39) |
| Tonga | 71227.4(63617.5-77646.8) | 63103.6(53380-72325.1) | -0.41(-0.42, -0.4) | 1044.8(650.4-1566) | 877.1(550.5-1295.7) | -0.57(-0.59, -0.56) |
| Trinidad and Tobago | 68619.1(61373.7-75419.7) | 61389(54941.7-68231.3) | -0.36(-0.36, -0.35) | 1155.7(723.7-1718.8) | 1025.4(621.7-1462.3) | -0.38(-0.39, -0.38) |
| Tunisia | 72531.7(65061.4-79057.9) | 71048.3(62806.8-78233.1) | -0.07(-0.07, -0.06) | 1181.2(744-1748.2) | 1079.1(677.5-1603.4) | -0.31(-0.33, -0.29) |
| Turkey | 75035(67288.4-81798.3) | 74343.7(66230-81495.2) | -0.03(-0.05, -0.02) | 1356.7(866.9-1970.3) | 1274.5(805.8-1879.8) | -0.21(-0.26, -0.18) |
| Turkmenistan | 68773.1(61092.5-75706.4) | 64716.5(55703.7-73562.4) | -0.19(-0.2, -0.19) | 1152.5(725-1707.3) | 1030.8(645.4-1535.9) | -0.35(-0.37, -0.34) |
| Tuvalu | 71923.8(64064.3-78556.5) | 69016.6(60488-77351.3) | -0.12(-0.13, -0.12) | 1070.7(660.9-1601.1) | 1174(718.6-1639.6) | 0.3(0.28, 0.32) |
| Uganda | 57476.2(49570.8-64785.3) | 50716.8(41756.7-59995.5) | -0.4(-0.42, -0.39) | 572.5(337.1-909) | 501.3(296.1-777.8) | -0.42(-0.44, -0.41) |
| Ukraine | 66439.2(59413.8-73298.2) | 67268.2(60007.1-74578.6) | 0.04(0.03, 0.04) | 1249.3(792.1-1835.7) | 1306.8(810.5-1822) | 0.14(0.13, 0.15) |
| United Arab Emirates | 71463.5(64346.7-77797.5) | 74774.6(66957-81888.7) | 0.14(0.14, 0.15) | 1229.4(784.7-1793.6) | 1374.9(874.2-2011.6) | 0.36(0.35, 0.37) |
| United Kingdom | 67678.6(60716.6-73624.9) | 64830.5(58709.9-70435.8) | -0.15(-0.16, -0.13) | 1033.3(651.8-1525.8) | 998.4(639.7-1436.2) | -0.11(-0.18, -0.05) |
| United Republic of Tanzania | 63436.9(55603.2-70320.3) | 57309.1(48680.6-65932.5) | -0.32(-0.33, -0.31) | 574.7(333.1-922.6) | 503.5(296.4-771.4) | -0.43(-0.44, -0.41) |
| United States of America | 60350.9(54098.7-66422.2) | 56502.3(50770.6-62309.2) | -0.2(-0.22, -0.18) | 1110.7(701.4-1657.4) | 931.2(591.5-1368.6) | -0.56(-0.61, -0.51) |
| United States Virgin Islands | 66316.4(58959.4-73306.8) | 63879.5(56147.5-71939.1) | -0.12(-0.13, -0.11) | 995.7(615.5-1510.5) | 902.6(558.2-1353.1) | -0.29(-0.34, -0.24) |
| Uruguay | 76808.7(69657-82520.8) | 75215.1(67544.9-81982.6) | -0.07(-0.07, -0.06) | 1193.8(754.7-1785.7) | 1066.4(668.8-1596.3) | -0.37(-0.4, -0.35) |
| Uzbekistan | 67861.4(60081.9-74815.7) | 63552.3(54849.9-72058.2) | -0.21(-0.23, -0.2) | 1031(641.8-1546.8) | 948.6(594.1-1415.6) | -0.26(-0.29, -0.24) |
| Vanuatu | 71828(64055-78297.1) | 64800.6(55013.9-73382.7) | -0.33(-0.35, -0.31) | 1068.3(665.3-1604.4) | 928.7(580-1351.8) | -0.44(-0.46, -0.43) |
| Venezuela (Bolivarian Republic of) | 75569.8(67646.7-82607.8) | 74961(66206.1-82747.5) | -0.03(-0.03, -0.02) | 1144.1(716.3-1695.9) | 1117.2(698.2-1665.6) | -0.07(-0.08, -0.07) |
| Viet Nam | 62550.2(53677.3-71256.8) | 59834.3(51417.1-68413.1) | -0.15(-0.18, -0.13) | 929.7(581.5-1376.2) | 808.9(497.3-1218) | -0.45(-0.5, -0.41) |
| Yemen | 70825.9(63479.2-77218.1) | 70567.7(62354.2-77956.8) | -0.01(-0.01, -0.01) | 1206.6(767.3-1765) | 1203.9(763.9-1768.3) | 0(-0.01, 0) |
| Zambia | 62015.4(55342.1-68074.4) | 49599.3(41230.3-58068.4) | -0.73(-0.75, -0.7) | 566.4(332-915.3) | 490.3(286.1-765) | -0.46(-0.48, -0.43) |
| Zimbabwe | 74528.6(66731.2-80968.9) | 73883.4(65118.9-81080.3) | 0.03(0.02, 0.04) | 723.2(451.5-1074.8) | 724.5(450.9-1064.9) | 0(-0.02, 0.03) |

Data in parentheses are 95% confidence intervals.

ASR, age-standardized rate; DALYs, disability-adjusted life years.

**Table S8 The Joinpoint analysis results in prevalence of oral diseases at the national level**

| Location | N | Segment 1 | | Segment 2 | | Segment 3 | | Segment 4 | | Segment 5 | | Segment 6 | |
| --- | --- | --- | --- | --- | --- | --- | --- | --- | --- | --- | --- | --- | --- |
|  |  | Period | APC  (95% CI) | Period | APC  (95% CI) | Period | APC  (95% CI) | Period | APC  (95% CI) | Period | APC  (95% CI) | Period | APC  (95% CI) |
| Afghanistan | 5 | 1990-1995 | 0.22(0.2, 0.24) | 1995-2000 | 0.1(0.08, 0.12) | 2000-2009 | -0.17(-0.18, -0.16) | 2009-2016 | -0.06(-0.07, -0.04) | 2016-2019 | -0.22(-0.24, -0.17) | 2019-2021 | 0.04(-0.02, 0.08) |
| Albania | 5 | 1990-1993 | 0.1(0.06, 0.16) | 1993-1996 | -0.01(-0.17, 0.02) | 1996-2000 | -0.18(-0.36, -0.16) | 2000-2006 | -0.36(-0.38, -0.24) | 2006-2014 | -0.24(-0.26, -0.21) | 2014-2021 | -0.12(-0.14, -0.1) |
| Algeria | 4 | 1990-1995 | 0.01(-0.01, 0.02) | 1995-2001 | -0.06(-0.07, -0.05) | 2001-2005 | -0.16(-0.17, -0.14) | 2005-2019 | -0.03(-0.03, -0.02) | 2019-2021 | 0.03(-0.01, 0.06) |  |  |
| American Samoa | 5 | 1990-1995 | 0.03(0.01, 0.04) | 1995-2005 | 0.06(0.06, 0.07) | 2005-2010 | -0.05(-0.05, -0.04) | 2010-2015 | 0.03(0.02, 0.04) | 2015-2019 | -0.05(-0.06, -0.04) | 2019-2021 | 0.32(0.29, 0.34) |
| Andorra | 4 | 1990-1994 | -0.3(-0.38, -0.25) | 1994-2010 | -0.12(-0.12, -0.11) | 2010-2015 | 0.2(0.17, 0.24) | 2015-2019 | -0.14(-0.21, -0.08) | 2019-2021 | 0.12(0, 0.2) |  |  |
| Angola | 4 | 1990-1994 | 0.1(0, 0.3) | 1994-2006 | -0.08(-0.11, -0.06) | 2006-2009 | -1.92(-2.03, -1.82) | 2009-2012 | -0.38(-0.48, -0.21) | 2012-2021 | 0(-0.04, 0.08) |  |  |
| Antigua and Barbuda | 5 | 1990-1996 | -0.18(-0.2, -0.16) | 1996-1999 | -0.37(-0.4, -0.31) | 1999-2006 | -0.09(-0.11, -0.06) | 2006-2009 | -0.22(-0.25, -0.16) | 2009-2019 | 0.02(0.01, 0.04) | 2019-2021 | -0.22(-0.29, -0.13) |
| Argentina | 4 | 1990-1995 | -0.04(-0.11, 0.03) | 1995-2000 | -0.53(-0.6, -0.46) | 2000-2005 | 0.36(0.28, 0.44) | 2005-2012 | -0.08(-0.21, -0.03) | 2012-2021 | 0.05(0.02, 0.14) |  |  |
| Armenia | 3 | 1990-2000 | 0.33(0.31, 0.35) | 2000-2005 | -1.09(-1.14, -1.05) | 2005-2015 | -0.32(-0.36, -0.29) | 2015-2021 | -0.06(-0.12, 0.04) |  |  |  |  |
| Australia | 5 | 1990-1992 | -6.34(-6.72, -6) | 1992-1995 | -3.89(-4.19, -3.6) | 1995-2000 | 2.09(1.92, 2.28) | 2000-2005 | -1.14(-1.3, -0.98) | 2005-2009 | 1.14(0.92, 1.47) | 2009-2021 | 0.19(0.12, 0.25) |
| Austria | 5 | 1990-1995 | -0.02(-0.2, 0.26) | 1995-2000 | -0.78(-1.05, -0.61) | 2000-2005 | 1.46(1.32, 1.61) | 2005-2010 | -2.33(-2.46, -2.2) | 2010-2014 | 1.42(1.16, 1.7) | 2014-2021 | 0.01(-0.15, 0.16) |
| Azerbaijan | 5 | 1990-1995 | 0.28(0.09, 0.36) | 1995-2000 | 0.57(0.5, 0.71) | 2000-2004 | -1.02(-1.16, -0.92) | 2004-2010 | -0.64(-0.83, -0.51) | 2010-2015 | -0.36(-0.51, -0.1) | 2015-2021 | 0.03(-0.05, 0.24) |
| Bahamas | 4 | 1990-1993 | -0.22(-0.28, -0.19) | 1993-1997 | -0.11(-0.13, -0.07) | 1997-2005 | -0.16(-0.19, -0.15) | 2005-2016 | -0.01(-0.08, 0) | 2016-2021 | 0.02(0, 0.07) |  |  |
| Bahrain | 4 | 1990-1995 | 0.01(0, 0.02) | 1995-2000 | -0.27(-0.28, -0.26) | 2000-2010 | -0.04(-0.05, -0.03) | 2010-2017 | 0.01(0, 0.04) | 2017-2021 | -0.04(-0.08, -0.02) |  |  |
| Bangladesh | 3 | 1990-1994 | 0.13(0.05, 0.29) | 1994-2000 | -0.08(-0.19, -0.02) | 2000-2019 | 0.01(-0.03, 0.09) | 2019-2021 | 0.19(0.01, 0.28) | 2014-2019 | -0.08 (-0.14 to -0.03) |  |  |
| Barbados | 5 | 1990-1995 | -0.05(-0.07, -0.03) | 1995-2000 | -0.29(-0.32, -0.27) | 2000-2010 | -0.09(-0.1, -0.08) | 2010-2015 | 0.06(0.04, 0.11) | 2015-2018 | -0.06(-0.09, -0.01) | 2018-2021 | 0.1(0.06, 0.17) |
| Belarus | 4 | 1990-1994 | 0.28(0.22, 0.35) | 1994-2000 | 0.06(0.03, 0.09) | 2000-2004 | -0.44(-0.52, -0.38) | 2004-2017 | -0.16(-0.18, -0.15) | 2017-2021 | 0.03(-0.04, 0.16) |  |  |
| Belgium | 5 | 1990-1995 | 0.34(0.28, 0.41) | 1995-2000 | -0.95(-1.08, -0.88) | 2000-2005 | -0.28(-0.45, -0.13) | 2005-2010 | 0.23(0.14, 0.41) | 2010-2016 | -0.18(-0.33, -0.11) | 2016-2021 | 0.22(0.11, 0.42) |
| Belize | 4 | 1990-1994 | -1.48(-1.53, -1.42) | 1994-2000 | -0.39(-0.45, -0.34) | 2000-2010 | -0.13(-0.25, -0.1) | 2010-2019 | -0.04(-0.11, 0.01) | 2019-2021 | 0.26(0.01, 0.4) |  |  |
| Benin | 4 | 1990-1994 | 0.08(0.01, 0.25) | 1994-2007 | -0.03(-0.06, -0.02) | 2007-2011 | -0.21(-0.33, -0.15) | 2011-2014 | -1.3(-1.38, -1.21) | 2014-2021 | -0.09(-0.13, -0.05) |  |  |
| Bermuda | 5 | 1990-1995 | -0.05(-0.05, -0.04) | 1995-1999 | -0.15(-0.16, -0.14) | 1999-2006 | -0.13(-0.18, -0.12) | 2006-2009 | -0.19(-0.21, -0.06) | 2009-2013 | -0.05(-0.07, -0.01) | 2013-2021 | 0.01(0.01, 0.02) |
| Bhutan | 4 | 1990-2004 | -0.05(-0.05, -0.05) | 2004-2010 | 0.03(0.02, 0.03) | 2010-2015 | -0.08(-0.09, -0.07) | 2015-2019 | 0.04(0.02, 0.05) | 2019-2021 | 0.2(0.17, 0.23) |  |  |
| Bolivia (Plurinational State of) | 5 | 1990-1996 | -0.03(-0.06, 0) | 1996-1999 | -0.27(-0.3, -0.19) | 1999-2007 | -0.12(-0.14, -0.08) | 2007-2011 | 0.04(-0.01, 0.11) | 2011-2014 | 0.61(0.56, 0.65) | 2014-2021 | -0.03(-0.05, -0.01) |
| Bosnia and Herzegovina | 4 | 1990-2001 | -0.2(-0.32, -0.1) | 2001-2005 | 1.89(1.55, 2.41) | 2005-2010 | -3.92(-4.15, -3.7) | 2010-2015 | 1.62(1.28, 2.07) | 2015-2021 | -0.19(-0.61, 0.13) |  |  |
| Botswana | 3 | 1990-1994 | -0.2(-0.48, -0.05) | 1994-2000 | 0.07(-0.76, 0.22) | 2000-2010 | -0.9(-0.94, -0.85) | 2010-2021 | -0.07(-0.1, -0.03) |  |  |  |  |
| Brazil | 3 | 1990-2001 | -0.06(-0.09, -0.04) | 2001-2005 | 0.82(0.77, 0.88) | 2005-2015 | -0.21(-0.24, -0.18) | 2015-2021 | 0(-0.06, 0.1) |  |  |  |  |
| Brunei Darussalam | 5 | 1990-1993 | -0.05(-0.1, 0.05) | 1993-1996 | -0.28(-0.33, -0.22) | 1996-1999 | -0.93(-0.98, -0.87) | 1999-2003 | -0.2(-0.27, -0.15) | 2003-2019 | -0.04(-0.06, -0.03) | 2019-2021 | 0.12(-0.02, 0.19) |
| Bulgaria | 5 | 1990-1995 | 0.06(-0.01, 0.16) | 1995-2000 | -0.28(-0.43, -0.21) | 2000-2005 | 0.02(-0.05, 0.12) | 2005-2010 | -1.13(-1.18, -1.07) | 2010-2015 | 0.73(0.68, 0.79) | 2015-2021 | -0.17(-0.22, -0.12) |
| Burkina Faso | 2 | 1990-1995 | 0.1(0.03, 0.2) | 1995-2015 | -0.09(-0.11, -0.08) | 2015-2021 | 0.03(-0.03, 0.21) |  |  |  |  |  |  |
| Burundi | 2 | 1990-2006 | 0.02(0, 0.04) | 2006-2009 | -2.6(-2.73, -2.48) | 2009-2021 | -0.03(-0.07, 0.02) |  |  |  |  |  |  |
| Cabo Verde | 4 | 1990-1995 | 0.07(0.03, 0.13) | 1995-2000 | -0.14(-0.23, -0.1) | 2000-2005 | 0.01(-0.03, 0.1) | 2005-2014 | -0.12(-0.2, -0.1) | 2014-2021 | -0.03(-0.06, 0.05) |  |  |
| Cambodia | 5 | 1990-1994 | -0.09(-0.14, -0.07) | 1994-2000 | -0.03(-0.04, 0.01) | 2000-2005 | -0.14(-0.18, -0.12) | 2005-2010 | -0.03(-0.05, 0) | 2010-2015 | -0.18(-0.21, -0.16) | 2015-2021 | -0.07(-0.09, -0.05) |
| Cameroon | 3 | 1990-1994 | 0.13(0.08, 0.25) | 1994-2005 | 0(-0.02, 0.02) | 2005-2010 | -0.11(-0.18, -0.06) | 2010-2021 | -0.04(-0.06, 0.02) |  |  |  |  |
| Canada | 5 | 1990-1995 | 0.02(-0.09, 0.13) | 1995-2000 | -0.81(-0.9, -0.71) | 2000-2005 | 1.1(1.02, 1.18) | 2005-2011 | -0.15(-0.21, -0.08) | 2011-2014 | -1.58(-1.71, -1.41) | 2014-2021 | -0.01(-0.08, 0.06) |
| Central African Republic | 4 | 1990-2002 | 0.02(0, 0.04) | 2002-2006 | -0.24(-0.35, -0.17) | 2006-2009 | -1.69(-1.78, -1.6) | 2009-2012 | -0.29(-0.36, -0.16) | 2012-2021 | -0.01(-0.04, 0.04) |  |  |
| Chad | 3 | 1990-2006 | 0.02(0, 0.03) | 2006-2011 | -0.37(-0.45, -0.29) | 2011-2014 | -1.28(-1.39, -1.12) | 2014-2021 | -0.05(-0.1, 0.01) |  |  |  |  |
| Chile | 5 | 1990-1995 | -0.06(-0.18, 0.06) | 1995-2000 | -0.74(-0.86, -0.64) | 2000-2004 | 0.13(0.03, 0.24) | 2004-2010 | -0.05(-0.16, 0.01) | 2010-2014 | 1.12(1.04, 1.2) | 2014-2021 | -0.05(-0.08, -0.02) |
| China | 5 | 1990-2000 | -0.48(-0.51, -0.44) | 2000-2005 | 0.86(0.8, 0.93) | 2005-2010 | -0.79(-0.92, -0.71) | 2010-2015 | 0(-0.12, 0.14) | 2015-2019 | 0.87(0.77, 1.04) | 2019-2021 | -0.1(-0.34, 0.2) |
| Colombia | 4 | 1990-2005 | -0.11(-0.28, 0.41) | 2005-2010 | 1.05(-0.55, 1.56) | 2010-2016 | -0.07(-0.14, 1.33) | 2016-2019 | -0.54(-0.71, -0.12) | 2019-2021 | -2.63(-3.56, -1.86) |  |  |
| Comoros | 3 | 1990-1994 | -1.86(-1.98, -1.71) | 1994-2006 | -0.03(-0.07, 0.01) | 2006-2009 | -3.45(-3.62, -3.29) | 2009-2021 | -0.12(-0.18, -0.06) |  |  |  |  |
| Congo | 4 | 1990-1995 | 0.08(0.01, 0.2) | 1995-2006 | -0.11(-0.14, -0.09) | 2006-2009 | -1.8(-1.87, -1.69) | 2009-2019 | -0.11(-0.22, -0.09) | 2019-2021 | 0.3(-0.04, 0.48) |  |  |
| Cook Islands | 4 | 1990-2008 | 0(-0.02, 0.02) | 2008-2011 | -0.47(-0.59, -0.28) | 2011-2014 | -3.79(-3.99, -3.63) | 2014-2017 | -0.74(-0.95, -0.46) | 2017-2021 | 0.17(-0.01, 0.55) |  |  |
| Costa Rica | 3 | 1990-1995 | 0.01(-0.01, 0.03) | 1995-2000 | -0.3(-0.32, -0.27) | 2000-2018 | -0.06(-0.07, -0.06) | 2018-2021 | 0.02(-0.04, 0.1) |  |  |  |  |
| Coted'Ivoire | 4 | 1990-1996 | 0.09(0.05, 0.15) | 1996-2005 | -0.08(-0.14, -0.05) | 2005-2010 | 0.08(0.03, 0.16) | 2010-2015 | -0.95(-0.99, -0.91) | 2015-2021 | -0.05(-0.09, -0.01) |  |  |
| Croatia | 4 | 1990-1997 | -0.2(-0.24, -0.07) | 1997-2005 | -0.29(-0.55, -0.26) | 2005-2010 | -0.97(-1.03, -0.9) | 2010-2015 | 0.96(0.9, 1.01) | 2015-2021 | -0.12(-0.18, -0.07) |  |  |
| Cuba | 4 | 1990-1994 | 0.04(-0.02, 0.12) | 1994-2006 | -0.12(-0.14, -0.11) | 2006-2015 | -0.48(-0.49, -0.46) | 2015-2019 | 0.07(0.02, 0.1) | 2019-2021 | 0.39(0.27, 0.48) |  |  |
| Cyprus | 5 | 1990-1995 | -0.14(-0.15, -0.12) | 1995-2000 | -0.39(-0.4, -0.38) | 2000-2010 | -0.08(-0.09, -0.08) | 2010-2015 | 0.03(0, 0.07) | 2015-2019 | -0.11(-0.14, -0.08) | 2019-2021 | 0.29(0.23, 0.34) |
| Czechia | 4 | 1990-1996 | 0(-0.04, 0.04) | 1996-1999 | -0.82(-0.87, -0.71) | 1999-2010 | -0.37(-0.38, -0.35) | 2010-2015 | 0.66(0.62, 0.7) | 2015-2021 | -0.13(-0.18, -0.09) |  |  |
| Democratic People's Republic of Korea | 2 | 1990-2001 | 0.09(0.06, 0.11) | 2001-2004 | 0.32(0.19, 0.37) | 2004-2021 | 0.07(0.06, 0.08) |  |  |  |  |  |  |
| Democratic Republic of the Congo | 3 | 1990-1994 | 0.2(0.09, 0.41) | 1994-2005 | 0.06(-1.37, 0.08) | 2005-2010 | -1.37(-1.41, -0.14) | 2010-2021 | -0.07(-0.11, -0.04) |  |  |  |  |
| Denmark | 5 | 1990-1996 | -0.28(-0.31, -0.22) | 1996-2000 | -0.55(-0.65, -0.47) | 2000-2005 | -0.3(-0.36, -0.16) | 2005-2010 | -0.59(-0.67, -0.53) | 2010-2015 | 0.83(0.79, 0.88) | 2015-2021 | -0.04(-0.09, 0) |
| Djibouti | 3 | 1990-1994 | -1.81(-1.95, -1.66) | 1994-2006 | -0.04(-0.09, 0) | 2006-2009 | -3.6(-3.78, -3.43) | 2009-2021 | -0.13(-0.19, -0.07) |  |  |  |  |
| Dominica | 2 | 1990-1995 | -0.09(-0.12, -0.03) | 1995-2000 | -0.25(-0.34, -0.21) | 2000-2021 | -0.09(-0.1, -0.09) |  |  |  |  |  |  |
| Dominican Republic | 4 | 1990-1996 | -0.11(-0.14, -0.07) | 1996-2000 | -0.57(-0.62, -0.51) | 2000-2005 | 0.14(0.1, 0.17) | 2005-2010 | -0.4(-0.46, -0.35) | 2010-2021 | -0.11(-0.13, -0.09) |  |  |
| Ecuador | 3 | 1990-2000 | -0.04(-0.08, 0) | 2000-2005 | -1.39(-1.46, -1.32) | 2005-2014 | 0.62(0.58, 0.68) | 2014-2021 | -0.06(-0.17, 0.03) |  |  |  |  |
| Egypt | 5 | 1990-1993 | 0.08(0.05, 0.11) | 1993-1996 | -0.09(-0.11, -0.06) | 1996-1999 | -0.4(-0.42, -0.37) | 1999-2011 | -0.12(-0.12, -0.11) | 2011-2019 | -0.03(-0.04, 0) | 2019-2021 | -0.11(-0.16, -0.05) |
| El Salvador | 3 | 1990-1994 | -0.25(-0.27, -0.22) | 1994-2010 | -0.07(-0.08, -0.07) | 2010-2015 | -0.01(-0.03, 0.03) | 2015-2021 | -0.09(-0.12, -0.07) |  |  |  |  |
| Equatorial Guinea | 4 | 1990-1996 | 0.01(-0.05, 0.05) | 1996-2006 | -0.39(-0.41, -0.37) | 2006-2009 | -1.66(-1.74, -1.59) | 2009-2012 | -0.21(-0.28, -0.1) | 2012-2021 | 0.01(-0.02, 0.07) |  |  |
| Eritrea | 2 | 1990-1996 | -0.12(-0.2, -0.05) | 1996-1999 | -2.12(-2.24, -2.01) | 1999-2021 | -0.03(-0.05, -0.02) |  |  |  |  |  |  |
| Estonia | 3 | 1990-2005 | 0.09(0.07, 0.12) | 2005-2010 | -2.01(-2.09, -1.93) | 2010-2014 | 1.06(0.87, 1.29) | 2014-2021 | 0.02(-0.1, 0.13) |  |  |  |  |
| Eswatini | 3 | 1990-2001 | -0.02(-0.04, 0) | 2001-2005 | -0.2(-0.31, -0.12) | 2005-2010 | 0.3(0.23, 0.42) | 2010-2021 | -0.03(-0.06, -0.01) |  |  |  |  |
| Ethiopia | 5 | 1990-1994 | 0.01(0, 0.03) | 1994-2000 | -0.07(-0.08, -0.06) | 2000-2005 | -0.2(-0.22, -0.19) | 2005-2009 | -0.06(-0.07, -0.04) | 2009-2014 | -0.12(-0.13, -0.1) | 2014-2021 | -0.17(-0.18, -0.16) |
| Fiji | 2 | 1990-2000 | -0.04(-0.08, 0) | 2000-2010 | -1.2(-1.25, -1.16) | 2010-2021 | -0.07(-0.13, -0.02) |  |  |  |  |  |  |
| Finland | 5 | 1990-1995 | -0.93(-1.21, -0.71) | 1995-1999 | 0.25(0.05, 0.49) | 1999-2005 | -0.15(-0.32, -0.07) | 2005-2010 | -2.79(-2.92, -2.65) | 2010-2018 | 1.16(1.03, 1.42) | 2018-2021 | 0.23(-0.56, 0.79) |
| France | 5 | 1990-1994 | -0.93(-1.11, -0.77) | 1994-2000 | 0.52(0.43, 0.71) | 2000-2005 | 0.05(-0.16, 0.17) | 2005-2010 | -0.96(-1.09, -0.84) | 2010-2014 | 0.92(0.74, 1.11) | 2014-2021 | 0.03(-0.06, 0.11) |
| Gabon | 4 | 1990-2003 | -0.01(-0.02, 0.01) | 2003-2006 | -0.17(-0.24, -0.08) | 2006-2009 | -1.55(-1.64, -1.45) | 2009-2012 | -0.23(-0.31, -0.12) | 2012-2021 | -0.03(-0.06, 0.04) |  |  |
| Gambia | 3 | 1990-1996 | 0.13(0.04, 0.19) | 1996-2000 | 0.67(0.59, 0.82) | 2000-2005 | -0.6(-0.7, -0.54) | 2005-2021 | -0.03(-0.05, -0.02) |  |  |  |  |
| Georgia | 5 | 1990-1999 | 0.44(0.43, 0.46) | 1999-2002 | 0.01(-0.06, 0.19) | 2002-2006 | -0.25(-0.31, -0.21) | 2006-2009 | -1.09(-1.15, -1.01) | 2009-2014 | -0.51(-0.58, -0.45) | 2014-2021 | -0.1(-0.13, -0.05) |
| Germany | 5 | 1990-1994 | -0.91(-1.02, -0.8) | 1994-2000 | 0.28(0.22, 0.33) | 2000-2007 | -1.2(-1.29, -1.15) | 2007-2010 | -0.56(-1, -0.04) | 2010-2015 | 0.44(0.31, 0.72) | 2015-2021 | -0.01(-0.2, 0.09) |
| Ghana | 5 | 1990-1995 | 0.04(0.01, 0.1) | 1995-2000 | -0.07(-0.14, -0.03) | 2000-2005 | 0.1(0.05, 0.16) | 2005-2010 | -0.27(-0.33, -0.22) | 2010-2019 | -0.04(-0.11, -0.03) | 2019-2021 | 0.13(-0.01, 0.19) |
| Greece | 5 | 1990-1995 | 0.12(0.03, 0.27) | 1995-2000 | -0.26(-0.43, -0.16) | 2000-2005 | 0.68(0.6, 0.76) | 2005-2010 | -1.39(-1.46, -1.31) | 2010-2014 | 0.65(0.52, 0.82) | 2014-2021 | 0.04(-0.04, 0.11) |
| Greenland | 5 | 1990-2003 | -0.03(-0.03, -0.02) | 2003-2006 | -0.18(-0.21, -0.12) | 2006-2009 | -0.79(-0.83, -0.75) | 2009-2012 | -0.11(-0.15, -0.06) | 2012-2016 | 0.06(0.02, 0.12) | 2016-2021 | -0.04(-0.09, -0.02) |
| Grenada | 4 | 1990-1996 | -0.16(-0.17, -0.14) | 1996-1999 | -0.41(-0.44, -0.38) | 1999-2010 | -0.14(-0.15, -0.13) | 2010-2015 | 0(-0.03, 0.05) | 2015-2021 | -0.12(-0.14, -0.1) |  |  |
| Guam | 4 | 1990-2003 | 0.02(-0.01, 0.09) | 2003-2006 | -0.25(-0.44, -0.06) | 2006-2009 | -3.8(-4, -3.56) | 2009-2014 | -0.64(-0.87, -0.44) | 2014-2021 | 0.06(-0.07, 0.24) |  |  |
| Guatemala | 5 | 1990-1995 | 0(-0.03, 0.03) | 1995-1999 | -0.34(-0.39, -0.3) | 1999-2004 | -0.21(-0.25, -0.15) | 2004-2015 | 0.01(-0.01, 0.03) | 2015-2018 | -0.16(-0.2, 0) | 2018-2021 | 0.01(-0.07, 0.11) |
| Guinea | 5 | 1990-1995 | 0.07(0.03, 0.14) | 1995-2000 | -0.08(-0.17, -0.03) | 2000-2005 | 0.08(0.01, 0.17) | 2005-2015 | -0.13(-0.17, -0.04) | 2015-2018 | 0.09(-0.15, 0.14) | 2018-2021 | -0.1(-0.25, 0) |
| Guinea-Bissau | 2 | 1990-2010 | -0.01(-0.02, 0) | 2010-2014 | -0.55(-0.63, -0.43) | 2014-2021 | -0.29(-0.33, -0.22) |  |  |  |  |  |  |
| Guyana | 4 | 1990-1995 | -0.1(-0.13, -0.08) | 1995-2000 | -0.39(-0.42, -0.33) | 2000-2004 | -0.04(-0.23, 0.01) | 2004-2019 | -0.11(-0.12, -0.08) | 2019-2021 | -0.24(-0.31, -0.12) |  |  |
| Haiti | 3 | 1990-1993 | -1.1(-1.15, -1.05) | 1993-1999 | -0.44(-0.46, -0.42) | 1999-2015 | -0.07(-0.08, -0.06) | 2015-2021 | 0(-0.03, 0.08) |  |  |  |  |
| Honduras | 5 | 1990-1996 | -0.08(-0.08, -0.07) | 1996-1999 | -0.17(-0.18, -0.14) | 1999-2005 | -0.06(-0.07, -0.05) | 2005-2010 | -0.15(-0.16, -0.14) | 2010-2015 | 0.06(0.05, 0.07) | 2015-2021 | -0.03(-0.04, -0.02) |
| Hungary | 5 | 1990-1996 | -0.03(-0.07, 0.02) | 1996-1999 | -1.52(-1.6, -1.44) | 1999-2005 | -0.45(-0.5, -0.41) | 2005-2011 | 0.18(0.12, 0.22) | 2011-2014 | 0.68(0.51, 0.75) | 2014-2021 | -0.15(-0.2, -0.11) |
| Iceland | 2 | 1990-1994 | -2.27(-2.35, -2.19) | 1994-2008 | -0.13(-0.17, -0.11) | 2008-2021 | -0.02(-0.05, 0.03) | 2011-2014 | -4.18 (-5.32 to -3.02) | 2014-2021 | -0.24 (-0.41 to -0.07) |  |  |
| India | 3 | 1990-2001 | -0.11(-0.19, -0.04) | 2001-2010 | 1.14(1.07, 1.23) | 2010-2019 | -1.38(-1.47, -1.32) | 2019-2021 | 0.07(-0.62, 0.47) | 2015-2019 | -3.47 (-3.8 to -3.14) |  |  |
| Indonesia | 5 | 1990-1995 | -0.1(-0.19, -0.05) | 1995-2000 | 0.36(0.29, 0.44) | 2000-2006 | -0.05(-0.08, 0) | 2006-2009 | -0.46(-0.5, -0.32) | 2009-2019 | -0.15(-0.19, -0.13) | 2019-2021 | 0.14(-0.07, 0.22) |
| Iran (Islamic Republic of) | 4 | 1990-2000 | 0(-0.02, 0.03) | 2000-2005 | -0.28(-0.36, -0.19) | 2005-2010 | -0.54(-0.6, -0.49) | 2010-2015 | 0.77(0.73, 0.81) | 2015-2021 | 0(-0.04, 0.04) |  |  |
| Iraq | 5 | 1990-1994 | 0.12(0.09, 0.14) | 1994-2000 | -0.03(-0.05, -0.01) | 2000-2005 | -0.15(-0.19, -0.13) | 2005-2010 | -0.08(-0.1, -0.04) | 2010-2019 | -0.13(-0.16, -0.12) | 2019-2021 | 0(-0.08, 0.03) |
| Ireland | 3 | 1990-2000 | -0.37(-0.43, -0.3) | 2000-2005 | 1.6(1.49, 1.73) | 2005-2010 | -1.64(-1.82, -1.51) | 2010-2021 | -0.12(-0.19, -0.04) |  |  |  |  |
| Israel | 4 | 1990-1995 | -0.05(-0.13, 0.07) | 1995-2000 | -0.45(-0.65, -0.37) | 2000-2005 | 0.47(0.39, 0.58) | 2005-2010 | -0.62(-0.78, -0.53) | 0.02(-0.01, 0.07) | 2010-2021 |  |  |
| Italy | 4 | 1990-2000 | -0.21(-0.26, -0.16) | 2000-2005 | 0.33(0.22, 0.5) | 2005-2010 | -1.23(-1.32, -1.14) | 2010-2014 | 1(0.84, 1.14) | 0.03(-0.05, 0.09) | 2014-2021 |  |  |
| Jamaica | 5 | 1990-1996 | -0.11(-0.12, -0.1) | 1996-1999 | -0.29(-0.3, -0.27) | 1999-2006 | -0.14(-0.15, -0.13) | 2006-2014 | -0.05(-0.06, -0.04) | 0.04(0.02, 0.06) | 2014-2019 | 2019-2021 | -0.21(-0.25, -0.17) |
| Japan | 5 | 1990-1995 | -0.68(-1.37, -0.27) | 1995-2000 | 1.18(0.78, 2.02) | 2000-2008 | -0.44(-0.77, -0.16) | 2008-2015 | -2.52(-2.99, -2.24) | 0.67(-0.52, 1.42) | 2015-2019 | 2019-2021 | 4.63(3, 5.95) |
| Jordan | 5 | 1990-1992 | 0.18(0.13, 0.22) | 1992-1995 | 0.09(-0.13, 0.11) | 1995-2009 | -0.13(-0.14, -0.13) | 2009-2014 | -0.1(-0.12, -0.06) | -0.01(-0.02, 0.03) | 2014-2019 | 2019-2021 | -0.15(-0.2, -0.1) |
| Kazakhstan | 5 | 1990-1997 | 0.2(0.17, 0.27) | 1997-2000 | 0.07(-0.03, 0.14) | 2000-2005 | -0.89(-0.93, -0.83) | 2005-2010 | -0.12(-0.18, -0.05) | -0.36(-0.49, -0.29) | 2010-2015 | 2015-2021 | -0.05(-0.1, 0.03) |
| Kenya | 4 | 1990-1994 | -0.65(-0.84, -0.56) | 1994-2000 | -0.04(-0.31, 0.02) | 2000-2005 | 0.08(-0.16, 0.19) | 2005-2011 | -0.12(-0.24, 0.04) | -0.02(-0.08, 0.07) | 2011-2021 |  |  |
| Kiribati | 3 | 1990-2001 | 0(-0.04, 0.03) | 2001-2004 | -1.55(-1.68, -1.4) | 2004-2010 | -0.67(-0.8, -0.52) | 2010-2021 | -0.05(-0.1, 0.01) |  |  |  |  |
| Kuwait | 3 | 1990-1995 | 0.03(0.01, 0.05) | 1995-1999 | -0.28(-0.32, -0.25) | 1999-2010 | -0.07(-0.08, -0.06) | 2010-2021 | 0.03(0.02, 0.04) |  |  |  |  |
| Kyrgyzstan | 5 | 1990-1994 | 0.23(0.04, 0.31) | 1994-2000 | 0.45(0.41, 0.54) | 2000-2004 | -0.89(-0.96, -0.83) | 2004-2011 | -0.21(-0.25, -0.13) | 2011-2015 | -0.44(-0.58, -0.33) | 2015-2021 | -0.04(-0.1, 0.06) |
| Lao People's Democratic Republic | 4 | 1990-1996 | -0.13(-0.21, -0.04) | 1996-2005 | -0.64(-0.69, -0.6) | 2005-2010 | 0.76(0.69, 0.83) | 2010-2017 | -0.35(-0.44, -0.29) | 2017-2021 | 0.13(-0.02, 0.38) |  |  |
| Latvia | 5 | 1990-1998 | 0.13(0.11, 0.18) | 1998-2001 | -0.14(-0.22, -0.03) | 2001-2007 | -1.11(-1.17, -1.07) | 2007-2010 | -0.52(-0.64, -0.44) | 2010-2015 | 0.84(0.78, 0.89) | 2015-2021 | 0.12(0.07, 0.17) |
| Lebanon | 3 | 1990-1995 | 0.1(0.09, 0.12) | 1995-2000 | -0.28(-0.3, -0.26) | 2000-2012 | -0.07(-0.08, -0.06) | 2012-2021 | -0.01(-0.02, 0.01) | 2011-2015 | 0.16 (0.11 to 0.2) |  |  |
| Lesotho | 2 | 1990-1994 | -0.12(-0.33, -0.02) | 1994-2004 | 0.06(0.03, 0.18) | 2004-2021 | -0.08(-0.1, -0.07) |  |  |  |  |  |  |
| Liberia | 5 | 1990-1994 | 0.26(0.22, 0.29) | 1994-2000 | -0.04(-0.08, -0.01) | 2000-2005 | 0.04(0, 0.09) | 2005-2014 | -0.14(-0.16, -0.05) | 2014-2019 | 0.03(-0.15, 0.1) | 2019-2021 | -0.09(-0.17, 0.01) |
| Libya | 4 | 1990-2004 | 0.01(0, 0.02) | 2004-2010 | -0.09(-0.12, -0.06) | 2010-2015 | 0.26(0.23, 0.31) | 2015-2019 | 0.16(0.09, 0.2) | 2019-2021 | 0.52(0.41, 0.6) |  |  |
| Lithuania | 5 | 1990-1995 | 0.21(0.17, 0.26) | 1995-1999 | -0.3(-0.37, -0.24) | 1999-2005 | -0.17(-0.21, -0.1) | 2005-2010 | -0.71(-0.75, -0.67) | 2010-2015 | 0.27(0.23, 0.34) | 2015-2021 | 0.07(0.01, 0.11) |
| Luxembourg | 3 | 1990-1995 | -0.18(-0.22, -0.15) | 1995-2005 | -0.49(-0.51, -0.48) | 2005-2015 | 0.2(0.19, 0.22) | 2015-2021 | -0.04(-0.08, 0) |  |  |  |  |
| Madagascar | 2 | 1990-1996 | -0.03(-0.13, 0.08) | 1996-1999 | -3.57(-3.73, -3.41) | 1999-2021 | -0.06(-0.08, -0.03) |  |  |  |  |  |  |
| Malawi | 3 | 1990-2003 | 0.03(0.01, 0.07) | 2003-2006 | -0.23(-0.35, -0.06) | 2006-2009 | -2.49(-2.62, -2.36) | 2009-2021 | -0.06(-0.1, -0.03) |  |  |  |  |
| Malaysia | 4 | 1990-1995 | -0.39(-0.71, -0.06) | 1995-2000 | 3.58(3.36, 3.85) | 2000-2005 | -1.02(-1.22, -0.83) | 2005-2009 | -3.89(-4.17, -3.61) | 2009-2021 | -0.24(-0.36, -0.12) |  |  |
| Maldives | 2 | 1990-2001 | -0.08(-0.1, -0.06) | 2001-2004 | -1.14(-1.2, -1.08) | 2004-2021 | -0.11(-0.12, -0.09) |  |  |  |  |  |  |
| Mali | 2 | 1990-2001 | -0.03(-0.05, -0.01) | 2001-2004 | -0.22(-0.26, -0.13) | 2004-2021 | -0.02(-0.03, -0.01) |  |  |  |  |  |  |
| Malta | 5 | 1990-1993 | -0.01(-0.04, 0.06) | 1993-1996 | -0.21(-0.24, -0.16) | 1996-1999 | -0.69(-0.73, -0.65) | 1999-2002 | -0.2(-0.24, -0.15) | 2002-2011 | 0.04(0.02, 0.05) | 2011-2021 | -0.14(-0.15, -0.12) |
| Marshall Islands | 2 | 1990-2000 | 0.07(0.02, 0.12) | 2000-2010 | -1.04(-1.11, -0.98) | 2010-2021 | -0.03(-0.1, 0.06) |  |  |  |  |  |  |
| Mauritania | 4 | 1990-2001 | 0(-0.05, 0.03) | 2001-2005 | 0.21(0.09, 0.32) | 2005-2011 | -0.28(-0.35, -0.23) | 2011-2014 | -1.48(-1.57, -1.38) | 2014-2021 | -0.06(-0.11, -0.01) |  |  |
| Mauritius | 3 | 1990-2000 | -0.17(-0.24, -0.1) | 2000-2005 | 1.63(1.5, 1.78) | 2005-2010 | -1.97(-2.18, -1.83) | 2010-2021 | -0.27(-0.35, -0.19) |  |  |  |  |
| Mexico | 5 | 1990-2001 | -0.22(-0.32, -0.18) | 2001-2005 | 0.08(-0.12, 0.26) | 2005-2010 | -0.89(-1.09, -0.76) | 2010-2015 | 0.33(0, 0.47) | 2015-2019 | 0.94(0.76, 1.21) | 2019-2021 | -0.09(-0.45, 0.33) |
| Micronesia (Federated States of) | 3 | 1990-2001 | -0.05(-0.09, -0.02) | 2001-2004 | -2.89(-3.06, -2.74) | 2004-2008 | -0.34(-0.56, -0.14) | 2008-2021 | -0.01(-0.05, 0.09) |  |  |  |  |
| Monaco | 5 | 1990-1996 | -0.07(-0.08, -0.06) | 1996-2001 | -0.19(-0.2, -0.17) | 2001-2004 | -0.54(-0.56, -0.52) | 2004-2007 | -0.09(-0.11, -0.06) | 2007-2019 | 0.01(0, 0.01) | 2019-2021 | 0.38(0.34, 0.41) |
| Mongolia | 4 | 1990-1998 | 0.15(0.14, 0.18) | 1998-2001 | -0.03(-0.08, 0.04) | 2001-2004 | -0.94(-0.99, -0.88) | 2004-2014 | -0.48(-0.5, -0.46) | 2014-2021 | -0.12(-0.15, -0.08) |  |  |
| Montenegro | 5 | 1990-1992 | 0.29(0.24, 0.33) | 1992-1995 | 0.14(0.07, 0.16) | 1995-2000 | -0.04(-0.06, -0.03) | 2000-2009 | -0.3(-0.3, -0.29) | 2009-2019 | -0.15(-0.15, -0.14) | 2019-2021 | 0.17(0.12, 0.21) |
| Morocco | 5 | 1990-1994 | -2.54(-2.63, -2.46) | 1994-2006 | -0.24(-0.27, -0.21) | 2006-2009 | -1.15(-1.27, -0.82) | 2009-2015 | -0.19(-0.35, -0.06) | 2015-2019 | 0.8(0.58, 1.08) | 2019-2021 | 0.01(-0.33, 0.46) |
| Mozambique | 4 | 1990-1995 | -0.25(-0.39, -0.16) | 1995-2003 | 0.03(-0.01, 0.18) | 2003-2006 | -0.25(-0.35, -0.11) | 2006-2009 | -2.42(-2.52, -2.31) | 2009-2021 | -0.12(-0.15, -0.09) |  |  |
| Myanmar | 3 | 1990-2000 | -0.07(-0.11, -0.02) | 2000-2005 | -2.06(-2.16, -1.96) | 2005-2010 | 0.78(0.63, 0.99) | 2010-2021 | -0.17(-0.24, -0.12) |  |  |  |  |
| Namibia | 5 | 1990-2000 | -0.03(-0.07, 0.01) | 2000-2005 | 1.2(1.12, 1.29) | 2005-2010 | -0.89(-0.98, -0.8) | 2010-2015 | 0.34(0.24, 0.45) | 2015-2019 | -1.07(-1.29, -0.94) | 2019-2021 | 0.09(-0.32, 0.37) |
| Nauru | 4 | 1990-1995 | 0.14(0.02, 0.24) | 1995-2005 | -0.92(-0.98, -0.87) | 2005-2011 | -0.11(-0.31, 0.07) | 2011-2014 | 1.02(0.71, 1.2) | 2014-2021 | 0.32(0.2, 0.39) |  |  |
| Nepal | 4 | 1990-1996 | 0.07(0.01, 0.16) | 1996-2001 | -0.26(-0.37, -0.19) | 2001-2005 | -1.2(-1.28, -1.13) | 2005-2010 | 0.46(0.38, 0.56) | 2010-2021 | 0.02(-0.01, 0.05) |  |  |
| Netherlands | 5 | 1990-1994 | -1.99(-2.24, -1.77) | 1994-2001 | -0.22(-0.41, -0.08) | 2001-2005 | 0.9(0.67, 1.24) | 2005-2010 | -2.55(-2.69, -2.41) | 2010-2015 | 1.68(1.49, 1.89) | 2015-2021 | -0.07(-0.29, 0.13) |
| New Zealand | 4 | 1990-1994 | -0.99(-1.27, -0.83) | 1994-2000 | -0.1(-0.25, 0.06) | 2000-2005 | 0.89(0.78, 1.07) | 2005-2016 | -0.49(-0.54, -0.46) | 2016-2021 | 0.28(0.13, 0.49) |  |  |
| Nicaragua | 1 | 1990-2019 | -0.08(-0.1, -0.07) | 2019-2021 | 0.17(-0.07, 0.28) |  |  |  |  |  |  |  |  |
| Niger | 4 | 1990-1995 | 0.16(0.05, 0.39) | 1995-2010 | -0.06(-0.1, -0.04) | 2010-2016 | -1.6(-1.65, -1.45) | 2016-2019 | -2.16(-2.37, -1.87) | 2019-2021 | 0.5(0.02, 0.91) |  |  |
| Nigeria | 4 | 1990-1995 | -0.13(-0.38, 0.2) | 1995-2006 | -1.23(-1.31, -1.16) | 2006-2009 | -5.89(-6.2, -5.59) | 2009-2014 | -1.44(-1.87, -1.13) | 2014-2021 | 0.67(0.44, 0.96) |  |  |
| Niue | 4 | 1990-1998 | 0.03(-0.01, 0.15) | 1998-2001 | -0.27(-0.43, -0.1) | 2001-2004 | -3.39(-3.59, -3.18) | 2004-2007 | -0.65(-0.8, -0.32) | 2007-2021 | -0.03(-0.07, 0.01) |  |  |
| North Macedonia | 5 | 1990-1995 | 0.11(0.07, 0.13) | 1995-2000 | 0.19(0.17, 0.21) | 2000-2005 | -0.17(-0.18, -0.15) | 2005-2009 | -0.38(-0.39, -0.36) | 2009-2015 | -0.17(-0.2, -0.16) | 2015-2021 | -0.11(-0.13, -0.08) |
| Northern Mariana Islands | 2 | 1990-1995 | -0.04(-0.17, 0.08) | 1995-2005 | -1.38(-1.44, -1.34) | 2005-2021 | 0.07(0.03, 0.1) |  |  |  |  |  |  |
| Norway | 5 | 1990-1996 | 0.16(-0.12, 0.57) | 1996-2005 | -0.98(-1.34, -0.82) | 2005-2010 | 1.82(1.39, 2.87) | 2010-2015 | -0.36(-1.16, 0.04) | 2015-2019 | 1.01(0.63, 1.68) | 2019-2021 | -3.38(-4.6, -1.88) |
| Oman | 3 | 1990-1996 | -0.08(-0.09, -0.06) | 1996-2005 | -0.12(-0.13, -0.11) | 2005-2010 | 0.02(0.01, 0.05) | 2010-2021 | -0.03(-0.03, -0.02) |  |  |  |  |
| Pakistan | 4 | 1990-2001 | 0.1(0.06, 0.13) | 2001-2005 | 1.37(1.26, 1.46) | 2005-2009 | -0.77(-0.95, -0.63) | 2009-2014 | -0.23(-0.46, -0.06) | 2014-2021 | 0.13(0.05, 0.28) |  |  |
| Palau | 5 | 1990-1996 | -0.18(-0.26, -0.09) | 1996-1999 | -3.51(-3.7, -3.34) | 1999-2005 | -0.17(-0.36, -0.05) | 2005-2009 | 1.13(0.89, 1.47) | 2009-2015 | 0.45(0.31, 0.62) | 2015-2021 | 0(-0.23, 0.11) |
| Palestine | 2 | 1990-1994 | -0.18(-0.25, -0.14) | 1994-2004 | -0.13(-0.14, -0.05) | 2004-2021 | -0.04(-0.05, -0.03) |  |  |  |  |  |  |
| Panama | 2 | 1990-2001 | -0.09(-0.09, -0.08) | 2001-2004 | -0.13(-0.15, -0.07) | 2004-2021 | -0.06(-0.07, -0.06) |  |  |  |  |  |  |
| Papua New Guinea | 3 | 1990-2001 | -0.04(-0.08, 0) | 2001-2004 | -2.66(-2.83, -0.04) | 2004-2007 | -0.41(-2.82, -0.17) | 2007-2021 | -0.1(-0.14, 0) |  |  |  |  |
| Paraguay | 3 | 1990-2000 | -0.02(-0.05, 0.02) | 2000-2005 | -1.69(-1.78, -1.61) | 2005-2009 | 1.26(1.13, 1.38) | 2009-2021 | -0.04(-0.08, 0) |  |  |  |  |
| Peru | 5 | 1990-1994 | -0.14(-0.2, -0.11) | 1994-2001 | -0.03(-0.05, 0.01) | 2001-2010 | -0.1(-0.11, -0.09) | 2010-2015 | 0.16(0.14, 0.18) | 2015-2019 | -0.5(-0.53, -0.47) | 2019-2021 | 0.09(0.02, 0.16) |
| Philippines | 3 | 1990-2000 | -0.03(-0.06, -0.01) | 2000-2005 | -0.91(-0.96, -0.87) | 2005-2010 | 0.23(0.16, 0.32) | 2010-2021 | -0.18(-0.2, -0.15) |  |  |  |  |
| Poland | 5 | 1990-2001 | -0.06(-0.13, 0) | 2001-2005 | 0.72(0.46, 1.05) | 2005-2011 | -0.25(-0.44, -0.11) | 2011-2014 | -2.85(-3.13, -2.43) | 2014-2018 | -0.28(-0.72, 0.08) | 2018-2021 | 1.69(1.22, 2.5) |
| Portugal | 5 | 1990-1996 | -0.25(-0.29, -0.21) | 1996-2000 | -0.53(-0.59, -0.41) | 2000-2005 | 0.21(-0.02, 0.25) | 2005-2010 | -0.33(-0.4, 0.18) | 2010-2019 | -0.05(-0.32, -0.03) | 2019-2021 | 0.16(-0.03, 0.25) |
| Puerto Rico | 5 | 1990-1996 | -0.12(-0.17, -0.05) | 1996-1999 | -0.91(-1.01, -0.7) | 1999-2005 | -0.12(-0.19, 0.02) | 2005-2010 | -0.66(-0.76, -0.56) | 2010-2015 | 1(0.92, 1.08) | 2015-2021 | -0.07(-0.15, 0) |
| Qatar | 5 | 1990-1996 | -0.12(-0.16, -0.07) | 1996-1999 | -0.56(-0.63, -0.15) | 1999-2005 | -0.22(-0.5, -0.1) | 2005-2010 | -0.32(-0.41, 0.33) | 2010-2015 | 0.36(-0.03, 0.43) | 2015-2021 | -0.07(-0.14, -0.01) |
| Republic of Korea | 5 | 1990-2001 | -0.37(-0.41, -0.29) | 2001-2004 | -0.91(-1.09, -0.56) | 2004-2010 | -0.15(-0.26, 0.1) | 2010-2015 | 2.02(1.89, 2.15) | 2015-2019 | -1.87(-2.06, -1.72) | 2019-2021 | 0.6(0.08, 0.98) |
| Republic of Moldova | 5 | 1990-1995 | 0.22(0.2, 0.25) | 1995-2000 | -0.18(-0.22, -0.15) | 2000-2005 | 0.02(-0.02, 0.09) | 2005-2010 | -0.1(-0.15, -0.07) | 2010-2014 | -0.3(-0.36, -0.25) | 2014-2021 | -0.07(-0.09, -0.04) |
| Romania | 4 | 1990-1994 | 0.16(0.03, 0.37) | 1994-2005 | -0.14(-0.18, -0.11) | 2005-2010 | -1.13(-1.19, -1.06) | 2010-2014 | 0.81(0.7, 0.9) | 2014-2021 | -0.07(-0.13, -0.02) |  |  |
| Russian Federation | 5 | 1990-2000 | 0.05(0.04, 0.06) | 2000-2005 | -0.3(-0.31, -0.28) | 2005-2011 | 0.07(0.05, 0.08) | 2011-2014 | 0.24(0.2, 0.27) | 2014-2019 | 0.06(0.03, 0.08) | 2019-2021 | 0.33(0.26, 0.38) |
| Rwanda | 3 | 1990-2003 | 0.03(0.01, 0.09) | 2003-2006 | -0.21(-0.41, -0.04) | 2006-2009 | -2.56(-2.7, -2.33) | 2009-2021 | -0.08(-0.12, -0.04) |  |  |  |  |
| Saint Kitts and Nevis | 3 | 1990-1997 | -0.18(-0.19, -0.16) | 1997-2005 | -0.25(-0.26, -0.24) | 2005-2018 | -0.07(-0.08, -0.07) | 2018-2021 | 0.03(-0.02, 0.09) |  |  |  |  |
| Saint Lucia | 4 | 1990-1995 | -0.12(-0.13, -0.1) | 1995-2000 | -0.37(-0.39, -0.36) | 2000-2005 | -0.03(-0.06, -0.01) | 2005-2010 | -0.16(-0.2, -0.13) | 2010-2021 | -0.04(-0.05, -0.03) |  |  |
| Saint Vincent and the Grenadines | 5 | 1990-1996 | -0.2(-0.22, -0.18) | 1996-1999 | -0.27(-0.29, -0.13) | 1999-2005 | -0.13(-0.19, -0.08) | 2005-2010 | -0.2(-0.24, -0.02) | 2010-2017 | 0.04(0.02, 0.07) | 2017-2021 | -0.04(-0.1, -0.02) |
| Samoa | 4 | 1990-1998 | 0.03(-0.01, 0.12) | 1998-2001 | -0.26(-0.38, -0.1) | 2001-2004 | -3.05(-3.22, -2.89) | 2004-2008 | -0.45(-0.66, -0.28) | 2008-2021 | 0(-0.04, 0.04) |  |  |
| San Marino | 5 | 1990-1996 | -0.15(-0.17, -0.12) | 1996-1999 | -0.51(-0.55, -0.43) | 1999-2010 | -0.1(-0.12, -0.09) | 2010-2015 | 0.13(0.1, 0.21) | 2015-2019 | -0.01(-0.09, 0.03) | 2019-2021 | 0.29(0.16, 0.37) |
| Sao Tome and Principe | 5 | 1990-1995 | 0.11(0.05, 0.21) | 1995-2000 | -0.14(-0.27, -0.07) | 2000-2005 | 0.09(0.03, 0.23) | 2005-2010 | -0.16(-0.27, -0.1) | 2010-2019 | -0.57(-0.6, -0.54) | 2019-2021 | 0.25(-0.02, 0.4) |
| Saudi Arabia | 4 | 1990-1992 | 0.53(0.31, 0.71) | 1992-1995 | 0.21(-0.43, 0.27) | 1995-2000 | -0.37(-0.48, 0.03) | 2000-2015 | -0.06(-0.16, -0.03) | 2015-2021 | 0.03(-0.04, 0.18) |  |  |
| Senegal | 3 | 1990-1994 | -1.71(-1.8, -1.6) | 1994-2005 | -0.02(-0.05, 0.02) | 2005-2015 | -0.68(-0.74, -0.62) | 2015-2021 | 0.01(-0.11, 0.19) |  |  |  |  |
| Serbia | 4 | 1990-2001 | 0.05(0.01, 0.09) | 2001-2004 | 1.26(1.06, 1.37) | 2004-2015 | -0.21(-0.24, -0.18) | 2015-2019 | -1.62(-1.8, -1.51) | 2019-2021 | 0.08(-0.47, 0.41) |  |  |
| Seychelles | 5 | 1990-1994 | -0.18(-0.28, -0.12) | 1994-2003 | 0(-0.01, 0.07) | 2003-2006 | -0.09(-1.07, -0.03) | 2006-2009 | -1.04(-1.1, -0.2) | 2009-2018 | -0.17(-0.21, -0.15) | 2018-2021 | 0.07(-0.03, 0.22) |
| Sierra Leone | 3 | 1990-1993 | -2.5(-3.7, -1.91) | 1993-2011 | -0.15(-0.2, -0.09) | 2011-2014 | 5.26(4.98, 5.57) | 2014-2021 | 0.12(-0.03, 0.25) |  |  |  |  |
| Singapore | 4 | 1990-1995 | -0.14(-0.17, -0.11) | 1995-2000 | -0.55(-0.58, -0.53) | 2000-2006 | -0.12(-0.17, -0.09) | 2006-2017 | -0.01(-0.02, 0.01) | 0.13(0.08, 0.23) | 2017-2021 |  |  |
| Slovakia | 5 | 1990-1995 | 0.08(0.02, 0.17) | 1995-2000 | -0.15(-0.27, -0.09) | 2000-2005 | 0.13(0.08, 0.21) | 2005-2010 | -0.84(-0.88, -0.8) | 0.04(-0.02, 0.12) | 2010-2014 | 2014-2021 | -0.12(-0.21, -0.08) |
| Slovenia | 4 | 1990-1994 | 0.05(-0.07, 0.29) | 1994-2005 | -0.22(-0.26, -0.19) | 2005-2010 | -1.1(-1.17, -1.02) | 2010-2015 | 0.76(0.65, 0.85) | -0.19(-0.29, -0.1) | 2015-2021 |  |  |
| Solomon Islands | 3 | 1990-2001 | -0.04(-0.07, 0) | 2001-2006 | -0.82(-0.91, -0.55) | 2006-2010 | -1.2(-1.42, -0.98) | 2010-2021 | -0.01(-0.06, 0.04) |  |  |  |  |
| Somalia | 2 | 1990-2000 | 0.05(0.02, 0.08) | 2000-2009 | -0.88(-0.92, -0.84) | 2009-2021 | -0.05(-0.08, -0.01) |  |  |  |  |  |  |
| South Africa | 5 | 1990-1995 | -0.47(-0.67, -0.33) | 1995-2000 | 0.35(0.23, 0.51) | 2000-2005 | -1.8(-1.95, -1.68) | 2005-2010 | -0.65(-0.82, -0.45) | 2010-2015 | 0.96(0.8, 1.21) | 2015-2021 | 0.01(-0.14, 0.13) |
| South Sudan | 3 | 1990-2000 | 0.03(0, 0.05) | 2000-2006 | -0.62(-0.68, -0.56) | 2006-2010 | -1.07(-1.18, -0.98) | 2010-2021 | 0.06(0.03, 0.08) |  |  |  |  |
| Spain | 4 | 1990-1996 | -0.7(-1.04, -0.45) | 1996-2001 | -1.53(-1.8, -0.43) | 2001-2010 | -1.83(-2.16, 3.37) | 2010-2014 | 3.33(-0.2, 3.65) | 2014-2021 | 0.11(-0.15, 0.55) |  |  |
| Sri Lanka | 4 | 1990-2000 | -0.07(-0.11, -0.03) | 2000-2006 | -1.29(-1.36, -0.72) | 2006-2010 | -1.65(-1.88, -1.44) | 2010-2014 | 1.07(0.83, 1.37) | 2014-2021 | -0.04(-0.17, 0.07) |  |  |
| Sudan | 5 | 1990-1993 | 0.1(0.09, 0.13) | 1993-1996 | 0.03(-0.02, 0.05) | 1996-2001 | -0.05(-0.08, -0.04) | 2001-2010 | -0.14(-0.14, -0.13) | 2010-2019 | -0.04(-0.04, -0.03) | 2019-2021 | 0.12(0.09, 0.14) |
| Suriname | 3 | 1990-1996 | -0.04(-0.06, -0.02) | 1996-1999 | -0.44(-0.47, -0.38) | 1999-2011 | -0.15(-0.17, -0.14) | 2011-2021 | -0.01(-0.03, 0) |  |  |  |  |
| Sweden | 5 | 1990-1995 | 0.35(0.11, 0.5) | 1995-2000 | 1.15(1.03, 1.32) | 2000-2011 | -0.17(-0.22, -0.12) | 2011-2014 | 1.09(-0.14, 1.22) | 2014-2019 | 0.08(-0.02, 0.94) | 2019-2021 | -0.64(-0.97, -0.18) |
| Switzerland | 4 | 1990-2006 | -0.09(-0.1, -0.09) | 2006-2010 | -0.26(-0.35, -0.2) | 2010-2015 | 0.16(0.12, 0.25) | 2015-2019 | -0.06(-0.15, -0.01) | 2019-2021 | 0.26(0.12, 0.36) |  |  |
| Syrian Arab Republic | 4 | 1990-1999 | -0.02(-0.04, 0) | 1999-2009 | -0.14(-0.17, -0.12) | 2009-2015 | 0.02(-0.11, 0.06) | 2015-2018 | 0.18(0.09, 0.22) | 2018-2021 | -0.13(-0.25, -0.07) |  |  |
| Taiwan (Province of China) | 3 | 1990-2001 | -0.02(-0.03, -0.01) | 2001-2014 | 0.08(0.04, 0.09) | 2014-2018 | -0.08(-0.14, 0.08) | 2018-2021 | 0.06(-0.02, 0.16) |  |  |  |  |
| Tajikistan | 4 | 1990-1996 | 0.34(0.31, 0.37) | 1996-2000 | 0.09(0.03, 0.15) | 2000-2006 | -0.17(-0.21, -0.14) | 2006-2015 | -0.33(-0.36, -0.31) | 2015-2021 | -0.08(-0.11, -0.03) |  |  |
| Thailand | 5 | 1990-1995 | -0.09(-0.11, -0.07) | 1995-2000 | -0.29(-0.31, -0.27) | 2000-2005 | 0.28(0.26, 0.29) | 2005-2010 | -0.16(-0.19, -0.14) | 2010-2019 | -0.09(-0.1, -0.08) | 2019-2021 | 0.13(0.05, 0.19) |
| Timor-Leste | 3 | 1990-2001 | -0.1(-0.12, -0.08) | 2001-2004 | -0.75(-0.81, -0.61) | 2004-2013 | -0.21(-0.25, -0.18) | 2013-2021 | 0.02(-0.02, 0.07) |  |  |  |  |
| Togo | 3 | 1990-1994 | -3.23(-3.36, -3.1) | 1994-2010 | -0.03(-0.06, 0) | 2010-2018 | -0.78(-0.98, -0.68) | 2018-2021 | -0.15(-0.53, 0.39) |  |  |  |  |
| Tokelau | 3 | 1990-1996 | -0.1(-0.23, 0.05) | 1996-1999 | -3.15(-3.36, -2.9) | 1999-2019 | -0.15(-0.19, -0.12) | 2019-2021 | 4.43(3.75, 4.97) |  |  |  |  |
| Tonga | 2 | 1990-2001 | -0.06(-0.1, -0.02) | 2001-2004 | -3.29(-3.43, -3.15) | 2004-2021 | -0.12(-0.16, -0.09) |  |  |  |  |  |  |
| Trinidad and Tobago | 5 | 1990-1993 | -0.92(-0.96, -0.87) | 1993-1996 | -0.4(-0.45, -0.36) | 1996-1999 | -1.04(-1.08, -1) | 1999-2009 | -0.32(-0.33, -0.3) | 2009-2015 | -0.16(-0.21, -0.12) | 2015-2021 | 0.03(0, 0.06) |
| Tunisia | 4 | 1990-1995 | 0.06(0.03, 0.09) | 1995-2000 | -0.2(-0.24, -0.16) | 2000-2005 | -0.42(-0.46, -0.39) | 2005-2009 | 0.25(0.2, 0.29) | 2009-2021 | -0.02(-0.03, -0.01) |  |  |
| Turkey | 5 | 1990-1995 | -0.08(-0.27, 0.03) | 1995-2002 | 0.74(0.5, 0.81) | 2002-2005 | 1(0.82, 1.09) | 2005-2011 | -0.5(-0.56, -0.45) | 2011-2014 | -1.51(-1.62, -1.37) | 2014-2021 | -0.17(-0.23, -0.1) |
| Turkmenistan | 4 | 1990-2000 | 0.35(0.33, 0.37) | 2000-2005 | -1.09(-1.14, -1.04) | 2005-2010 | -0.23(-0.3, -0.12) | 2010-2015 | -0.46(-0.62, -0.38) | 2015-2021 | -0.1(-0.17, 0) |  |  |
| Tuvalu | 4 | 1990-2001 | -0.1(-0.13, -0.08) | 2001-2004 | -1.61(-1.69, -1.52) | 2004-2011 | 0.1(0.04, 0.15) | 2011-2014 | 0.66(0.46, 0.76) | 2014-2021 | -0.08(-0.15, -0.03) |  |  |
| Uganda | 2 | 1990-2000 | 0.04(0, 0.09) | 2000-2010 | -1.3(-1.35, -1.26) | 2010-2021 | 0.01(-0.05, 0.07) |  |  |  |  |  |  |
| Ukraine | 5 | 1990-1998 | 0.12(0.1, 0.14) | 1998-2005 | -0.03(-0.06, -0.01) | 2005-2010 | -0.2(-0.25, -0.17) | 2010-2015 | 0.33(0.3, 0.36) | 2015-2019 | -0.14(-0.2, -0.11) | 2019-2021 | 0.19(0.08, 0.27) |
| United Arab Emirates | 5 | 1990-1996 | 0.12(0.09, 0.14) | 1996-2001 | 0.25(0.2, 0.29) | 2001-2005 | 0.38(0.35, 0.42) | 2005-2010 | -0.17(-0.19, -0.15) | 2010-2016 | 0.25(0.23, 0.27) | 2016-2021 | 0.07(0.04, 0.1) |
| United Kingdom | 5 | 1990-1995 | -0.39(-0.58, -0.25) | 1995-2000 | 0.38(0.27, 0.55) | 2000-2007 | -1.18(-1.32, -1.12) | 2007-2010 | -0.63(-0.85, -0.06) | 2010-2015 | 1.28(1.19, 1.41) | 2015-2021 | -0.12(-0.21, -0.04) |
| United Republic of Tanzania | 3 | 1990-2003 | 0.06(0.04, 0.1) | 2003-2006 | -0.22(-0.35, -0.04) | 2006-2009 | -2.84(-2.98, -2.69) | 2009-2021 | -0.11(-0.15, -0.07) |  |  |  |  |
| United States of America | 5 | 1990-2001 | -0.34(-0.47, -0.26) | 1993-2001 | -0.16(-0.19, -0.09) | 2001-2004 | -1.55(-1.64, -1.46) | 2004-2010 | -0.21(-0.25, -0.15) | 2014-2018 | 1.12(0.84, 1.44) | 2015-2021 | 0.07(0.01, 0.13) |
| United States Virgin Islands | 5 | 1990-1993 | -0.37(-0.56, -0.23) | 2001-2006 | -1.46(-1.55, -0.22) | 2006-2010 | -1.81(-2.06, -1.58) | 2010-2014 | 1.85(1.62, 2.13) | 2010-2015 | 0.85(0.78, 0.9) | 2018-2021 | 0.17(-0.28, 0.45) |
| Uruguay | 2 | 1990-1995 | -0.07(-0.11, -0.02) | 1995-1999 | -0.33(-0.39, -0.29) | 1999-2021 | -0.02(-0.02, -0.01) |  |  |  |  |  |  |
| Uzbekistan | 5 | 1990-1995 | 0.13(-0.02, 0.19) | 1995-1998 | 0.51(0.37, 0.61) | 1998-2001 | 0.05(-0.06, 0.16) | 2001-2004 | -1.81(-1.9, -1.71) | 2004-2018 | -0.27(-0.3, -0.25) | 2018-2021 | 0.1(-0.09, 0.39) |
| Vanuatu | 3 | 1990-2006 | -0.01(-0.05, 0.01) | 2006-2009 | -3.04(-3.23, 0.01) | 2009-2012 | -0.37(-3.25, -0.1) | 2012-2021 | 0.05(-0.04, 0.31) |  |  |  |  |
| Venezuela (Bolivarian Republic of) | 5 | 1990-1993 | -0.08(-0.12, -0.05) | 1993-2005 | -0.02(-0.03, -0.01) | 2005-2010 | -0.17(-0.19, -0.03) | 2010-2015 | -0.04(-0.18, -0.02) | 2015-2019 | 0.15(-0.03, 0.17) | 2019-2021 | 0.08(0.04, 0.13) |
| Viet Nam | 5 | 1990-1995 | -0.11(-0.29, 0.08) | 1995-2000 | -2.25(-2.4, -2.1) | 2000-2005 | 0.79(0.66, 0.93) | 2005-2010 | -0.37(-0.49, -0.26) | 2010-2015 | 1.28(1.11, 1.45) | 2015-2021 | -0.2(-0.4, -0.01) |
| Yemen | 4 | 1990-1994 | -0.19(-0.21, -0.18) | 1994-2000 | 0.03(0.02, 0.04) | 2000-2010 | -0.12(-0.13, -0.11) | 2010-2015 | 0.04(0.03, 0.06) | 2015-2021 | 0.21(0.2, 0.22) |  |  |
| Zambia | 3 | 1990-1994 | -2.11(-2.24, -1.96) | 1994-2011 | -0.05(-0.08, -0.02) | 2011-2014 | -3.78(-3.99, -3.59) | 2014-2021 | -0.23(-0.36, -0.1) |  |  |  |  |
| Zimbabwe | 3 | 1990-2001 | 0(-0.03, 0.04) | 2001-2005 | -0.51(-0.7, -0.38) | 2005-2010 | 0.62(0.5, 0.76) | 2010-2021 | -0.02(-0.05, 0.02) |  |  |  |  |

N represents the number of turning points in the jointpoint regression analysis.

CI, confidence interval; APC, annual percentage change.

**Table S9 The prevalence and DALYs of dental carious and their trends from 1990 to 2021 at the national level**

| Location | **Prevalence** | | | **DALYs** | | |
| --- | --- | --- | --- | --- | --- | --- |
|  | ASR in 1990 | ASR in 2021 | AAPC (1990-2021) | ASR in 1990 | ASR in 2021 | AAPC (1990-2021) |
| Afghanistan | 46772.3(34379.4-58438.5) | 47572.9(34242.6-59428.3) | 0.05(0.05, 0.06) | 42.8(19-82.3) | 42.8(19.1-81.8) | 0.01(0, 0.01) |
| Albania | 36916.8(25371.6-49789.5) | 34361.5(23241-47363.1) | -0.24(-0.25, -0.23) | 34.3(14.8-65.2) | 32(13.6-63.5) | -0.22(-0.24, -0.22) |
| Algeria | 42681.4(30710.3-54849.9) | 42833.3(30587.9-54980) | 0.01(0, 0.01) | 39.5(17.4-76.8) | 39.3(17.5-74.8) | -0.02(-0.02, -0.01) |
| American Samoa | 40073.9(28060.7-52102.1) | 41776(29443.8-53632.9) | 0.14(0.13, 0.14) | 36.9(16.3-71.9) | 38(16.6-72.4) | 0.09(0.09, 0.1) |
| Andorra | 45931(33103.2-57784.7) | 44667.7(32309.4-56444.3) | -0.1(-0.11, -0.08) | 42.9(18.8-81.6) | 41.6(18.3-80.3) | -0.11(-0.12, -0.09) |
| Angola | 34396.3(23392.6-46262.8) | 32763.7(21736.2-44357.9) | -0.16(-0.17, -0.15) | 31.6(13.5-62.2) | 30.1(12.7-58.5) | -0.16(-0.17, -0.15) |
| Antigua and Barbuda | 33757.4(22451.3-46226.5) | 32003.1(21521-44235.1) | -0.16(-0.17, -0.15) | 31.5(13.3-60.8) | 29.5(12.9-57.1) | -0.2(-0.21, -0.18) |
| Argentina | 50341.9(37336.3-62226.3) | 51488.9(38947.5-62466) | 0.06(0.04, 0.09) | 46.9(20.7-88) | 47.9(21.5-88.6) | 0.06(0.03, 0.08) |
| Armenia | 37757.3(25927.7-50570) | 36603.5(25117.4-49333.4) | -0.11(-0.13, -0.1) | 35.1(15.2-68.6) | 34(14.6-66.1) | -0.11(-0.13, -0.1) |
| Australia | 34962(26142.3-44887.9) | 30138(20048.5-41396.6) | -0.48(-0.51, -0.44) | 32.3(14.2-61.2) | 27.8(11.7-54.1) | -0.48(-0.52, -0.45) |
| Austria | 37378(26072.3-48399.7) | 37120.3(26263-48251.6) | -0.04(-0.06, -0.02) | 34.7(15.2-67) | 34.6(15.5-67.4) | -0.03(-0.05, -0.01) |
| Azerbaijan | 36382.8(24753.3-48947.6) | 35617.5(24133.1-48475.2) | -0.06(-0.07, -0.05) | 34.1(14.5-66.7) | 33.2(14-65.6) | -0.08(-0.09, -0.07) |
| Bahamas | 37441(25993.2-50070.7) | 36670.2(24916.6-49424.7) | -0.04(-0.06, -0.03) | 35(15.2-68.7) | 34(14.8-65.7) | -0.08(-0.09, -0.07) |
| Bahrain | 40659.8(28617.7-52649.7) | 39885.4(27727.2-52690.7) | -0.07(-0.07, -0.06) | 37.4(16.2-71.8) | 36.2(15.9-69.4) | -0.11(-0.12, -0.11) |
| Bangladesh | 41726(30045-52786.8) | 40612.7(29250.9-52494.1) | -0.1(-0.13, -0.08) | 38.4(17.1-73.1) | 37.4(16.6-72.1) | -0.1(-0.12, -0.08) |
| Barbados | 33334.1(22430.5-45854) | 33329.1(22676.4-45842.1) | 0.01(0, 0.02) | 31.2(13.4-61.6) | 30.9(13.3-60.6) | -0.02(-0.03, -0.01) |
| Belarus | 27878.8(17893.6-39436.7) | 27684.4(18229.5-38964.4) | -0.04(-0.07, -0.02) | 25.9(10.9-51.3) | 25.6(10.8-50) | -0.05(-0.08, -0.03) |
| Belgium | 34461.6(23805.8-45948.2) | 32878.4(22145.5-44106.5) | -0.15(-0.17, -0.14) | 32.1(13.9-62.7) | 30.4(13.1-60.2) | -0.17(-0.18, -0.16) |
| Belize | 36525.3(25071.7-49459.5) | 27336.7(17144.5-39925) | -0.9(-0.94, -0.87) | 34.2(14.9-66.9) | 25.5(9.9-52.5) | -0.92(-0.95, -0.89) |
| Benin | 35837(24706.3-47649.1) | 34838.7(24035.3-46340.8) | -0.12(-0.14, -0.1) | 33(14.1-64.5) | 32.1(13.8-61.4) | -0.09(-0.14, -0.05) |
| Bermuda | 31421.2(20917.4-43424.1) | 29923.5(19726.2-42303.3) | -0.16(-0.16, -0.15) | 29.4(12.7-57.6) | 27.9(11.7-55.1) | -0.17(-0.18, -0.16) |
| Bhutan | 40984.6(29533.3-52778.7) | 38257.8(26687-50215.7) | -0.22(-0.23, -0.22) | 37.9(16.7-73.6) | 35.4(15.2-68.1) | -0.22(-0.22, -0.21) |
| Bolivia (Plurinational State of) | 52153.7(39277.3-63758.8) | 50887.6(37158.4-62828.7) | -0.07(-0.08, -0.06) | 48.3(21.9-91.6) | 46.9(20.9-89.6) | -0.09(-0.1, -0.08) |
| Bosnia and Herzegovina | 38157.7(26013.1-51221.2) | 34018.8(22572.4-46627.1) | -0.37(-0.38, -0.36) | 35.2(15.2-68.2) | 31.3(13.2-60.7) | -0.38(-0.39, -0.37) |
| Botswana | 27818.6(18479.1-38207) | 25725.2(16516.2-36098) | -0.25(-0.25, -0.24) | 25.8(10.9-50.3) | 23.6(9.8-47.9) | -0.28(-0.29, -0.28) |
| Brazil | 37846(26468.2-50025.7) | 39411.9(27865.1-51230) | 0.1(0.05, 0.16) | 34.6(15-65.3) | 36(15.8-67.9) | 0.11(0.06, 0.17) |
| Brunei Darussalam | 11866.2(7630.1-17692.8) | 12041.1(7630-17891) | 0.05(0.04, 0.05) | 10.9(4.4-22.1) | 11.1(4.5-22) | 0.04(0.03, 0.05) |
| Bulgaria | 38330.2(25881.7-52178.2) | 37080.1(24993.4-50255.9) | -0.11(-0.11, -0.1) | 35.4(15.3-69.1) | 34.3(14.7-66.7) | -0.1(-0.1, -0.09) |
| Burkina Faso | 34997.6(23939.9-46682.4) | 33849.3(22911.7-45443.4) | -0.14(-0.17, -0.11) | 32.3(13.9-63.6) | 31.4(13.5-60.8) | -0.11(-0.13, -0.08) |
| Burundi | 42482.4(30503.6-54405.4) | 43616(30997.8-55123.7) | 0.08(0.07, 0.08) | 39.3(17-74.7) | 40.5(18-78.1) | 0.09(0.09, 0.1) |
| Cabo Verde | 35291.2(23847.7-46631.7) | 32503.6(21899.7-43917.8) | -0.29(-0.31, -0.26) | 32.9(14.2-63.5) | 30.1(12.8-59.3) | -0.3(-0.33, -0.28) |
| Cambodia | 51196.1(36999.2-63978.8) | 48135.9(34516.5-61781) | -0.18(-0.19, -0.18) | 47(20.8-89.9) | 44.3(19.4-84.5) | -0.17(-0.18, -0.16) |
| Cameroon | 34032.4(23074.6-45848.7) | 34259.1(23173.2-45949.6) | -0.01(-0.04, 0.02) | 31.4(13.5-60.7) | 31.7(13.7-62.3) | 0(-0.02, 0.03) |
| Canada | 20237.3(13054.7-29147.5) | 19582.3(12609.8-28641.8) | -0.11(-0.13, -0.1) | 18.9(7.6-38.5) | 18.2(7.4-36.6) | -0.13(-0.14, -0.12) |
| Central African Republic | 36843.4(25469.9-48373.2) | 37525.7(25995.6-48996.3) | 0.08(0.07, 0.09) | 33.6(14.5-64.7) | 34.3(14.9-66.6) | 0.08(0.06, 0.09) |
| Chad | 36963(25586.3-48544.7) | 35661.9(24656.3-47548.3) | -0.15(-0.16, -0.13) | 34.1(15.1-64.7) | 32.9(14.4-64.5) | -0.14(-0.15, -0.12) |
| Chile | 51724.6(38321.8-62926.9) | 54873.6(52990-56781.4) | 0.15(0.1, 0.23) | 47.9(21.3-90.8) | 50.8(23.9-95.7) | 0.18(0.16, 0.2) |
| China | 18710(12517.8-25798.5) | 19241.2(13813.2-25557.2) | 0.1(0.08, 0.12) | 17.4(7.5-33.9) | 17.9(7.8-34.5) | 0.1(0.08, 0.11) |
| Colombia | 36003.7(25542-47422.9) | 33988.1(23004-45501.4) | -0.29(-0.48, -0.17) | 33.4(14.6-63.9) | 31.6(13.5-61.6) | -0.28(-0.47, -0.18) |
| Comoros | 31909.3(24532.4-39958.4) | 23600.6(14804.5-33879.5) | -0.92(-0.94, -0.89) | 29.6(13.1-58.5) | 22(8.7-46.4) | -0.92(-0.95, -0.89) |
| Congo | 33771.8(23174.5-45386.1) | 33242.4(22282.3-45265.2) | -0.04(-0.06, -0.03) | 31.2(13.4-60.7) | 30.6(12.9-59.8) | -0.05(-0.07, -0.04) |
| Cook Islands | 40861.6(28903.7-53155.1) | 39975.4(27959.1-52271.3) | -0.06(-0.07, -0.05) | 37.6(16.4-72.6) | 36.6(15.6-70.3) | -0.08(-0.09, -0.07) |
| Costa Rica | 42188.9(29469.5-54984.5) | 40524.4(28643.7-53167.3) | -0.14(-0.15, -0.12) | 39.1(16.9-75.7) | 37.4(16.2-72.5) | -0.15(-0.16, -0.14) |
| Coted'Ivoire | 33954.6(23029.3-45222.5) | 33746.6(22772.2-44559) | -0.04(-0.05, -0.02) | 30.9(13.4-60.2) | 31.2(13.4-60.9) | 0.01(-0.01, 0.03) |
| Croatia | 37847.4(26263.3-50649.1) | 36919.8(25577-49482.9) | -0.09(-0.1, -0.08) | 34.9(15-66.5) | 34(14.7-65.3) | -0.08(-0.1, -0.07) |
| Cuba | 34843.9(23435.2-47585.7) | 31376.5(23467-40978) | -0.34(-0.35, -0.33) | 32.5(14-63.3) | 29.1(13-55.5) | -0.35(-0.36, -0.34) |
| Cyprus | 48220.5(35473-59921) | 46191(33274.4-58012.4) | -0.14(-0.14, -0.14) | 44.7(19.5-84) | 43(19.4-81.2) | -0.13(-0.13, -0.12) |
| Czechia | 33304.7(22222.7-46133.7) | 32380.7(21039.4-45133.2) | -0.11(-0.13, -0.1) | 30.5(13.3-59.6) | 29.7(12.8-57.7) | -0.09(-0.1, -0.08) |
| Democratic People's Republic of Korea | 11400.6(7079.5-16849.3) | 12578.1(7736.6-18322.1) | 0.33(0.3, 0.35) | 10.7(4.3-21.8) | 11.8(4.7-24) | 0.32(0.3, 0.35) |
| Democratic Republic of the Congo | 35555.3(24466-47458.5) | 37375.1(25925.1-48777.2) | 0.16(0.16, 0.17) | 32.5(14.1-63.4) | 34.3(14.8-67) | 0.17(0.17, 0.18) |
| Denmark | 24828.4(16103-34203.2) | 20752.8(12865.9-29822.9) | -0.56(-0.58, -0.55) | 23.1(9.7-45.2) | 19.5(7.4-40.9) | -0.53(-0.55, -0.51) |
| Djibouti | 30952.7(23258.8-38738.5) | 22452.2(14097.6-32367) | -1.04(-1.07, -1.01) | 28.9(12.6-55.9) | 21(8.2-43.5) | -1.02(-1.05, -0.99) |
| Dominica | 35085.6(23899.9-47632.9) | 33670.1(22781.8-45953.5) | -0.11(-0.13, -0.1) | 32.8(13.9-65.4) | 31.2(13.5-60.7) | -0.14(-0.16, -0.12) |
| Dominican Republic | 35803.3(24755.2-48748.2) | 32738.6(21606.6-45616.9) | -0.29(-0.3, -0.28) | 33.5(14.6-64) | 30.4(12.8-59.9) | -0.31(-0.33, -0.29) |
| Ecuador | 50460.9(37670.3-62864.6) | 50325.6(36883.8-62401) | -0.01(-0.01, 0) | 46.9(20.9-89.1) | 46.5(21-87.7) | -0.03(-0.04, -0.02) |
| Egypt | 36701.7(25246.6-48940.3) | 34934.4(23618.2-47596.1) | -0.16(-0.16, -0.15) | 34(14.9-64.9) | 32.1(13.6-62.9) | -0.19(-0.2, -0.18) |
| El Salvador | 44123.4(31140.8-56752.3) | 41700.7(29412.2-54338.4) | -0.18(-0.19, -0.16) | 40.9(18.2-79) | 38.6(16.8-74.1) | -0.18(-0.19, -0.17) |
| Equatorial Guinea | 35782.1(24658-47768.9) | 29483.9(19591.5-40738.2) | -0.62(-0.63, -0.61) | 32.5(14.2-63.5) | 27(11.6-52.6) | -0.59(-0.6, -0.58) |
| Eritrea | 48485.5(36027.6-59963.5) | 47728.3(35266.9-59569.1) | -0.08(-0.1, -0.06) | 44.5(19.8-84.9) | 43.9(19.7-83.2) | -0.07(-0.09, -0.05) |
| Estonia | 16093.2(10019.1-24191.8) | 14769.1(9164.8-22237.7) | -0.28(-0.29, -0.28) | 14.9(6-29.8) | 13.7(5.6-28) | -0.28(-0.29, -0.27) |
| Eswatini | 27949.1(18588.1-38190) | 26993.2(17623.9-37589.9) | -0.12(-0.13, -0.11) | 25.9(10.9-51.7) | 24.7(10.6-47.8) | -0.17(-0.18, -0.16) |
| Ethiopia | 33171.5(25796.1-41200.3) | 29751.9(22812.2-37573.6) | -0.35(-0.36, -0.35) | 30.3(13.4-58.7) | 27.4(12.1-52.6) | -0.32(-0.33, -0.32) |
| Fiji | 43125.4(31577.7-54250) | 42560.3(30221.3-54629.9) | -0.04(-0.05, -0.03) | 39.6(17.2-75) | 38.7(16.7-73.4) | -0.07(-0.08, -0.07) |
| Finland | 28500(19441.9-38175.1) | 27256.8(18273.5-36999.4) | -0.2(-0.25, -0.17) | 26.3(11.4-50.7) | 25.2(10.8-49.5) | -0.16(-0.19, -0.13) |
| France | 56406.2(44016.3-67160.9) | 55169.3(41246.2-66377.7) | -0.07(-0.07, -0.06) | 52.2(24.7-98.7) | 51.1(23.5-96.9) | -0.07(-0.07, -0.06) |
| Gabon | 30469.4(20306.3-41836.2) | 30487.8(20250.6-41955.4) | 0(0, 0.01) | 28.1(11.7-56.8) | 28(11.9-55.4) | -0.01(-0.01, -0.01) |
| Gambia | 34745.2(23622.2-46311) | 35059.3(24195.2-46090) | 0.02(0, 0.04) | 32.3(13.9-63) | 32.3(14-62.5) | 0(-0.02, 0.02) |
| Georgia | 35971.2(24857.7-48198.6) | 36604.1(24654-49258.3) | 0.05(0.04, 0.05) | 33.6(14.7-65.7) | 33.9(14.6-66.8) | 0.03(0.02, 0.03) |
| Germany | 41608.6(29523.6-53053) | 28771.4(19040.3-39767.6) | -1.16(-1.18, -1.11) | 38.7(17-74.1) | 26.8(10.8-55.3) | -1.15(-1.18, -1.12) |
| Ghana | 29125.2(19350.3-40106.2) | 27424.8(17695.3-38270) | -0.22(-0.25, -0.19) | 27(11.5-54) | 25.4(10.8-49.7) | -0.21(-0.25, -0.19) |
| Greece | 51950.5(39456-62633) | 50713.8(38297.9-62047.9) | -0.08(-0.09, -0.08) | 48.4(21.8-90.5) | 47.3(20.9-89.7) | -0.08(-0.08, -0.07) |
| Greenland | 19372.1(12421.3-28357.9) | 18630.6(11912-27073.3) | -0.13(-0.13, -0.12) | 17.9(7.4-36.2) | 17.3(7-34.9) | -0.11(-0.12, -0.1) |
| Grenada | 35887.7(24585.3-48450.2) | 33406.7(22330.9-45870.3) | -0.23(-0.23, -0.22) | 33.5(14.1-65) | 30.8(13.2-60.9) | -0.26(-0.27, -0.26) |
| Guam | 38311.2(26511.5-50292.3) | 38566.4(26667.5-50477.5) | 0.03(0.01, 0.05) | 35.7(15.5-69.2) | 35.9(15.5-68.6) | 0.02(0, 0.04) |
| Guatemala | 43565.2(31174.4-56524.6) | 41821.7(29157-55065.9) | -0.13(-0.15, -0.12) | 40.1(17.6-77.5) | 38.3(16.7-73.4) | -0.14(-0.16, -0.13) |
| Guinea | 35626.7(24617.4-47434.9) | 35121(24294.5-46847.4) | -0.07(-0.1, -0.06) | 33(14.4-64.7) | 32.5(14.2-62.7) | -0.07(-0.09, -0.05) |
| Guinea-Bissau | 45719.6(34048.9-56870.5) | 45238.4(33573.1-56755.8) | -0.03(-0.06, -0.01) | 42.2(18.6-79) | 41.8(18.5-80.1) | 0(-0.02, 0.02) |
| Guyana | 36958.6(25405.3-49110.1) | 34180(23158.2-46396.8) | -0.24(-0.25, -0.23) | 34(14.6-65.2) | 31.3(13-61.5) | -0.26(-0.27, -0.24) |
| Haiti | 38519.8(26930.8-51572.4) | 31957.5(21070.6-44661.5) | -0.6(-0.61, -0.58) | 35.6(15.1-69.1) | 29.5(11.6-58.8) | -0.61(-0.64, -0.59) |
| Honduras | 44291.2(31581-56334.1) | 43447.1(30805.1-55743.7) | -0.06(-0.07, -0.06) | 41.1(18.2-77.7) | 40(17.5-76) | -0.08(-0.09, -0.08) |
| Hungary | 30166.1(19531.5-42750.6) | 29083(19063.7-40955.1) | -0.12(-0.12, -0.11) | 27.6(11.6-54.2) | 26.8(11.4-52.3) | -0.08(-0.09, -0.08) |
| Iceland | 48410.9(40506-55598.4) | 35890(25512.1-46399.6) | -0.95(-0.98, -0.92) | 45.3(21.1-88.1) | 33.7(14.3-69.2) | -0.93(-0.96, -0.9) |
| India | 47167.1(36804.1-56526.7) | 43884.1(34280.5-52718.9) | -0.24(-0.29, -0.2) | 42.5(19.2-81.2) | 39.8(18-76.5) | -0.22(-0.27, -0.18) |
| Indonesia | 42521.6(30455.6-54771.3) | 40931.8(29208.6-52754.4) | -0.13(-0.17, -0.11) | 39.2(17.2-74.1) | 37.8(16.5-71.2) | -0.13(-0.17, -0.11) |
| Iran (Islamic Republic of) | 40193(31259.5-49035.1) | 39191.4(30395-48043.2) | -0.08(-0.09, -0.08) | 37(16.6-70.5) | 36(16.2-69.4) | -0.1(-0.1, -0.09) |
| Iraq | 43140(30534-55528.4) | 42843.7(30098.4-55167.3) | -0.03(-0.05, -0.02) | 39.7(17.6-75.5) | 39(17.2-74.7) | -0.07(-0.08, -0.06) |
| Ireland | 48672.1(36325.4-60212.9) | 45782.3(33303.6-57190.2) | -0.21(-0.24, -0.19) | 45.4(20-86) | 42.7(19.2-80.1) | -0.21(-0.24, -0.19) |
| Israel | 56619.6(43408.7-67814.1) | 54830.3(41928-65811.7) | -0.1(-0.12, -0.07) | 52.9(23.8-101.4) | 51.2(22.9-97.8) | -0.1(-0.15, -0.06) |
| Italy | 37927.2(26905.6-48585.1) | 36692.1(26445.4-46577.2) | -0.11(-0.12, -0.1) | 35(15.4-67.9) | 34.1(15.1-65.4) | -0.09(-0.1, -0.08) |
| Jamaica | 35226.2(24276-47751.8) | 34282.7(23042.9-46788.1) | -0.08(-0.09, -0.08) | 33(14.3-64.3) | 31.9(13.9-62.7) | -0.11(-0.11, -0.1) |
| Japan | 8728.3(5922.9-12517.3) | 5743.5(4194.9-7765.3) | -1.35(-1.41, -1.28) | 8.1(3.4-15.8) | 5.4(2.3-10.2) | -1.33(-1.39, -1.27) |
| Jordan | 44237(31809.9-56801.9) | 43360.3(30896.7-55132.3) | -0.06(-0.07, -0.06) | 40.8(18.2-79.3) | 39.7(17.7-77.1) | -0.09(-0.1, -0.08) |
| Kazakhstan | 35925.5(24233.1-48750.8) | 34743.3(23332.5-47576.5) | -0.12(-0.13, -0.1) | 33.3(14.3-66.7) | 32.1(13.9-63.3) | -0.12(-0.14, -0.11) |
| Kenya | 30862.7(22202.7-39961.8) | 28275.4(20666.2-36363.4) | -0.27(-0.3, -0.24) | 28.4(12.6-54.7) | 26(11.6-50.3) | -0.29(-0.32, -0.27) |
| Kiribati | 45153.4(32999.6-56876.3) | 46151.6(34239.2-57661.9) | 0.08(0.07, 0.08) | 41.3(17.9-78.6) | 42.2(19.1-80) | 0.07(0.07, 0.08) |
| Kuwait | 39611.3(27255.9-52294.4) | 39325.7(27225-51611.5) | -0.02(-0.03, -0.02) | 36.7(16.1-70.9) | 36(15.8-70) | -0.06(-0.07, -0.06) |
| Kyrgyzstan | 37795.5(25964-50750.9) | 39007.7(26913.4-51764.9) | 0.09(0.09, 0.1) | 35.2(15.5-67.4) | 36.4(15.5-70.1) | 0.1(0.1, 0.11) |
| Lao People's Democratic Republic | 38580.1(26679.1-51431.4) | 34550.9(23352.5-47101.7) | -0.35(-0.36, -0.34) | 35.8(15.8-68.6) | 32(13.6-61.8) | -0.36(-0.37, -0.34) |
| Latvia | 27817.6(18391.4-39000.9) | 26696.6(17392.4-37860.8) | -0.14(-0.14, -0.13) | 25.7(10.9-51.3) | 24.6(10.5-48.5) | -0.14(-0.15, -0.13) |
| Lebanon | 43276.7(30928.1-55121.4) | 42887.8(30424.6-55337.7) | -0.03(-0.04, -0.03) | 39.8(17.6-76.4) | 39.1(17.1-74.9) | -0.06(-0.07, -0.05) |
| Lesotho | 30717.1(20770-41044.2) | 29192.8(19594.6-39411.3) | -0.17(-0.2, -0.15) | 28.5(12.1-55.9) | 26.7(11.4-52.2) | -0.23(-0.26, -0.2) |
| Liberia | 35918.3(25172.5-47373.3) | 36735.7(25546.5-48327) | 0.06(0.04, 0.07) | 32.9(14.3-63.7) | 33.6(14.6-65.7) | 0.05(0.03, 0.07) |
| Libya | 39759.7(27948.1-51839.2) | 44325.8(31795.9-56712.6) | 0.34(0.32, 0.35) | 36.9(16.1-71.5) | 40.5(17.8-78.4) | 0.3(0.28, 0.31) |
| Lithuania | 34624.9(23495.5-47295) | 33168.6(22481.8-45636.5) | -0.14(-0.16, -0.13) | 32(13.8-62.4) | 30.6(13-59.7) | -0.16(-0.17, -0.15) |
| Luxembourg | 46136.2(33877.2-57478.7) | 43753.6(31629.7-56025) | -0.17(-0.17, -0.16) | 42.9(18.8-80) | 40.8(18.2-78) | -0.16(-0.17, -0.16) |
| Madagascar | 58601.7(45805.6-69570) | 59222.5(46530.9-69905.5) | 0.03(0.01, 0.04) | 54.2(25.2-101.8) | 55(25.5-103.2) | 0.04(0.03, 0.05) |
| Malawi | 43164.4(30549-54827.6) | 42749(30651.7-54775.3) | -0.04(-0.06, -0.03) | 39.8(17.1-76.7) | 39.5(17.4-75.7) | -0.04(-0.06, -0.03) |
| Malaysia | 28164.1(18729.4-38503) | 25674.1(16845.9-35660.7) | -0.29(-0.3, -0.28) | 26(11-51.7) | 23.6(9.9-47.2) | -0.3(-0.31, -0.3) |
| Maldives | 38659.3(26334.1-51914.5) | 35824.4(24104.7-48458.3) | -0.24(-0.25, -0.23) | 35.9(15.3-68.9) | 33.3(14.2-65.5) | -0.24(-0.25, -0.23) |
| Mali | 37117.9(26294.3-48652.8) | 35184.5(24150.3-46834.5) | -0.19(-0.21, -0.18) | 34.1(14.5-66.1) | 32.4(13.7-62.8) | -0.19(-0.21, -0.17) |
| Malta | 49071.5(35926.4-60650.3) | 46010(33477.5-57257.4) | -0.21(-0.21, -0.2) | 45.9(20.2-88.1) | 43(19.3-81) | -0.21(-0.21, -0.2) |
| Marshall Islands | 44440.9(32636.7-56179.7) | 44596.5(32647.9-56570.4) | 0.02(0, 0.04) | 40.9(18.2-78.3) | 40.7(17.8-78.2) | 0(-0.02, 0.02) |
| Mauritania | 34781.2(24010.5-46507.4) | 33817.9(23273.9-45558.6) | -0.12(-0.14, -0.1) | 32.3(13.9-63.3) | 31.5(13.5-61.9) | -0.1(-0.12, -0.09) |
| Mauritius | 37658.1(25867.5-50135.7) | 34925.5(23770.8-47874.8) | -0.24(-0.24, -0.23) | 34.7(14.8-67.6) | 31.9(13.7-62) | -0.27(-0.27, -0.26) |
| Mexico | 26144.2(17497.9-36189.4) | 25877.7(17505.7-35571.4) | -0.03(-0.05, -0.02) | 23.9(10.3-46.7) | 23.8(10.3-46.3) | -0.02(-0.03, 0) |
| Micronesia (Federated States of) | 45093.2(32460.3-56819.9) | 44673.5(32915.3-56168) | -0.02(-0.04, -0.01) | 41.7(18.4-78.6) | 41(18-79.2) | -0.05(-0.06, -0.03) |
| Monaco | 43440.9(31003.2-55763.9) | 41458.5(28978.9-53868.2) | -0.15(-0.16, -0.15) | 40.8(17.7-79) | 38.8(16.9-76.3) | -0.16(-0.17, -0.16) |
| Mongolia | 38325.9(26351.1-51492.5) | 36372.1(24619.6-48997.5) | -0.18(-0.19, -0.16) | 35.7(15.3-69.1) | 33.8(14.7-65.5) | -0.18(-0.2, -0.16) |
| Montenegro | 36653.3(24799.4-48891.9) | 36053.2(24898.6-48649.4) | -0.05(-0.06, -0.05) | 34.1(14.5-66.8) | 33.4(14.4-64.8) | -0.07(-0.08, -0.06) |
| Morocco | 31811.4(24358.5-39508.1) | 15817.1(9440.4-24479.7) | -2.23(-2.35, -2.15) | 29.5(13.2-57.3) | 14.5(5.5-30.2) | -2.27(-2.38, -2.18) |
| Mozambique | 47791.5(35076.2-60543.6) | 43230.7(30667.4-55152.5) | -0.31(-0.32, -0.31) | 43.6(19.3-82.7) | 39.6(17.7-74.2) | -0.29(-0.3, -0.28) |
| Myanmar | 29429.1(19118.1-41108.6) | 24978.5(15951.6-35823.6) | -0.51(-0.58, -0.45) | 27.1(11.3-53.1) | 23(9.5-45.6) | -0.56(-0.59, -0.52) |
| Namibia | 33249.8(22813.8-44065.3) | 31757(21432-42577) | -0.15(-0.19, -0.12) | 30.9(13.2-59.5) | 29.4(12.6-58) | -0.17(-0.2, -0.14) |
| Nauru | 40520.1(28650.2-52656.2) | 41417.8(29372.3-53232.2) | 0.08(0.07, 0.1) | 37.4(16.3-72.9) | 37.8(16.6-71.4) | 0.05(0.04, 0.07) |
| Nepal | 39706.5(28325.8-50631.6) | 36648.2(25671.2-47747.2) | -0.27(-0.29, -0.25) | 36.1(15.8-69.4) | 33.4(14.7-63.6) | -0.26(-0.28, -0.23) |
| Netherlands | 32948.5(25961.4-40039.1) | 21383.4(13302.5-30672) | -1.35(-1.38, -1.3) | 30.9(14-62) | 20(7.9-42.1) | -1.35(-1.39, -1.3) |
| New Zealand | 27278.8(18102.8-38284) | 26788.8(17760.9-37668.3) | -0.04(-0.08, 0) | 25.1(10.7-49.9) | 24.8(10.4-48.8) | -0.03(-0.07, 0.01) |
| Nicaragua | 44163.5(31618.9-56604.3) | 43237(30657.4-55943.3) | -0.08(-0.12, -0.05) | 40.9(17.8-78.8) | 39.9(17.5-76.3) | -0.09(-0.11, -0.07) |
| Niger | 39907.3(28363-51345.7) | 39674.5(28407.5-51131.3) | -0.03(-0.07, 0) | 36.9(16.1-70.9) | 36.8(16.1-71) | -0.03(-0.07, 0) |
| Nigeria | 18630.9(13393.2-24570.5) | 17337.4(12568.4-22892.1) | -0.23(-0.24, -0.22) | 17.1(7.5-32.8) | 16(7-31) | -0.21(-0.22, -0.2) |
| Niue | 42258.6(30417.7-54479.6) | 41871(29377.2-53929.6) | -0.03(-0.04, -0.03) | 39(16.9-75.7) | 38.4(16.5-73.8) | -0.06(-0.06, -0.05) |
| North Macedonia | 36118.6(24889.4-48799.3) | 35303.3(23990.3-47560.2) | -0.07(-0.08, -0.07) | 33.3(14.4-63.3) | 32.5(13.6-62.7) | -0.08(-0.09, -0.07) |
| Northern Mariana Islands | 38767.5(26809.6-51011.2) | 39914.5(27700.6-51974.8) | 0.11(0.09, 0.12) | 35.9(15.6-68.6) | 36.8(16.1-69.7) | 0.09(0.08, 0.11) |
| Norway | 40230(29627.8-50596.1) | 38905.8(28643-49029.7) | -0.23(-0.47, -0.11) | 37.2(16.7-70.9) | 36.2(16.1-69.1) | -0.21(-0.45, -0.08) |
| Oman | 44476.8(31754.7-56853.3) | 43734.1(30735.4-55655.9) | -0.06(-0.06, -0.05) | 40.7(17.9-77.8) | 40(17.6-75.8) | -0.06(-0.07, -0.06) |
| Pakistan | 32233.7(22075.6-42771.1) | 35319.1(24731.8-46230.5) | 0.28(0.25, 0.31) | 29.6(12.7-57.7) | 32.3(14-63.7) | 0.25(0.19, 0.32) |
| Palau | 41507.8(29370.9-53515.6) | 41589.3(29196.8-53866.7) | 0.01(0, 0.01) | 38.3(16.6-73) | 38(16.5-73) | -0.02(-0.03, -0.01) |
| Palestine | 38261.9(26026.9-50277.7) | 37137.3(25680.8-49940.2) | -0.1(-0.11, -0.09) | 35.3(15.2-68.2) | 34(14.8-66.5) | -0.13(-0.14, -0.12) |
| Panama | 54880.7(41327.2-66732.8) | 52420.2(39193.4-64898.6) | -0.14(-0.15, -0.13) | 50.9(22.9-96.6) | 48.5(21.7-92.7) | -0.16(-0.16, -0.15) |
| Papua New Guinea | 44824(32639.3-56349.3) | 44241.6(32395.7-55207.8) | -0.03(-0.05, 0) | 41.2(18-78.1) | 40.6(17.5-77.1) | -0.02(-0.05, 0) |
| Paraguay | 44891(32261.5-56957.3) | 44061.5(30933.2-56710.4) | -0.07(-0.08, -0.06) | 41.6(18.3-80.1) | 40.6(17.7-76.2) | -0.09(-0.1, -0.07) |
| Peru | 51200(37666.1-63133.7) | 49730.6(36945.1-61924) | -0.09(-0.11, -0.08) | 47.7(21.3-90) | 46.3(20.7-88.1) | -0.1(-0.12, -0.09) |
| Philippines | 43333.9(33069.6-53579.6) | 43358.9(34524.3-52204.4) | 0.01(0, 0.02) | 39.8(18-75.4) | 40(18-76.8) | 0.02(0.01, 0.03) |
| Poland | 27813.5(20938.9-35578.2) | 26515.6(20019.8-34155.8) | -0.17(-0.2, -0.14) | 25.5(11-49.3) | 24.4(10.6-47.3) | -0.15(-0.17, -0.13) |
| Portugal | 40254.4(28552.6-52256.4) | 37924.1(25571.5-50463) | -0.21(-0.24, -0.18) | 37.5(16.3-73.6) | 35.4(15.4-69.9) | -0.2(-0.23, -0.17) |
| Puerto Rico | 32213.2(21461.2-44853.1) | 31118.9(20809.7-43099.3) | -0.1(-0.11, -0.09) | 29.9(12.7-57.8) | 28.7(12.3-56) | -0.13(-0.14, -0.12) |
| Qatar | 38165.1(26454.1-50578.8) | 38030.7(26108.1-50661.7) | -0.03(-0.04, 0) | 35(15.3-67.4) | 34.4(14.8-66.7) | -0.07(-0.1, -0.05) |
| Republic of Korea | 21033.1(13380.8-30257.8) | 18223.1(11469.4-26640.6) | -0.46(-0.47, -0.45) | 19.4(8.1-39.1) | 16.9(7-34.2) | -0.44(-0.46, -0.43) |
| Republic of Moldova | 29897.9(19666.7-42017.7) | 29983.1(19770.1-41600.6) | 0(-0.02, 0.02) | 27.7(11.6-53.9) | 27.8(11.9-54.5) | 0(-0.02, 0.02) |
| Romania | 39879.9(27913.2-52619.5) | 38698.4(27288.6-51846.7) | -0.1(-0.1, -0.09) | 37(15.8-73.1) | 36(15.5-67.6) | -0.09(-0.09, -0.08) |
| Russian Federation | 32251.1(22696.4-43177.9) | 32393.6(23371-42754.5) | 0.01(0, 0.01) | 29.7(13-57) | 29.8(13.2-56.7) | 0.01(0, 0.01) |
| Rwanda | 43010.1(30881.8-55600.4) | 41037.5(28907-53256.2) | -0.15(-0.16, -0.15) | 39.8(17.3-77.2) | 38.1(16.6-72.6) | -0.14(-0.15, -0.13) |
| Saint Kitts and Nevis | 33924.9(23247.9-46132.9) | 31648.8(21236.2-43621.8) | -0.22(-0.23, -0.21) | 31.5(13.5-61.7) | 29.3(12.4-57) | -0.24(-0.25, -0.23) |
| Saint Lucia | 35293.6(24059.6-47994.8) | 33657.9(22590.4-46469.4) | -0.15(-0.16, -0.15) | 32.7(14-62.8) | 31(13.3-60.7) | -0.16(-0.17, -0.16) |
| Saint Vincent and the Grenadines | 43561.2(30793.6-56351.1) | 41791.9(29824.1-54885.2) | -0.12(-0.13, -0.11) | 40.5(17.5-77.7) | 38.5(17.1-75.2) | -0.15(-0.16, -0.13) |
| Samoa | 44035.3(31803.3-56049.8) | 43884.9(31768.3-55593.2) | -0.01(-0.02, -0.01) | 40.6(17.8-77.3) | 40.3(18.1-77) | -0.02(-0.03, -0.02) |
| San Marino | 46270.4(33568-58405.2) | 45270(33143.5-57642.3) | -0.07(-0.08, -0.07) | 43.2(19.2-83.9) | 42.2(19.1-80.4) | -0.07(-0.08, -0.07) |
| Sao Tome and Principe | 35115(24081.2-46393.2) | 34205.5(23177.2-45958.4) | -0.1(-0.11, -0.09) | 32.6(14-63.3) | 31.6(13.7-61.2) | -0.11(-0.12, -0.1) |
| Saudi Arabia | 51449.3(38373-64224.7) | 51286.8(37748.4-64274) | -0.04(-0.06, -0.02) | 46.7(21.1-88.3) | 46.5(20.9-89.1) | -0.04(-0.06, -0.02) |
| Senegal | 27494.6(20824.8-34816.9) | 18659.9(11724.4-27254.8) | -1.18(-1.21, -1.15) | 25.5(11-50.2) | 17.3(6.5-35.9) | -1.17(-1.21, -1.14) |
| Serbia | 33799.4(22925.5-45482.3) | 32276.5(21342.6-43847) | -0.16(-0.26, -0.08) | 31.3(13.3-60.5) | 29.8(12.8-57.6) | -0.17(-0.23, -0.12) |
| Seychelles | 36590.6(25338.5-49481.2) | 34643.8(23173.2-47031.1) | -0.16(-0.18, -0.15) | 34(14.5-66.2) | 31.7(13.3-61.5) | -0.21(-0.23, -0.19) |
| Sierra Leone | 33755.5(26281.7-41763.9) | 26317.5(17388.9-36306.2) | -0.79(-0.82, -0.77) | 31.3(13.5-61) | 24.5(9.6-50) | -0.77(-0.8, -0.74) |
| Singapore | 13380.3(8400.7-20195.8) | 11851.5(7517-17577.9) | -0.39(-0.4, -0.38) | 12.5(5.1-25.1) | 11.1(4.5-22.3) | -0.38(-0.39, -0.37) |
| Slovakia | 34083.7(22771.2-46930.6) | 32300.6(21428.3-44568.3) | -0.17(-0.18, -0.16) | 31.3(13.6-61.5) | 29.8(12.6-57.5) | -0.15(-0.16, -0.14) |
| Slovenia | 30813.1(20393.1-42855.3) | 29490.6(19282.5-41629.8) | -0.14(-0.16, -0.11) | 28.3(12-55.8) | 27.2(11.5-53.3) | -0.12(-0.14, -0.1) |
| Solomon Islands | 45997(33986.1-57213.7) | 45481.8(33423.8-56938.2) | 0(-0.02, 0.02) | 42.6(19-81.9) | 41.9(18.5-79.1) | -0.02(-0.04, 0) |
| Somalia | 46433.6(33467.2-58376.3) | 47007.6(34667.9-58853.6) | 0.04(0.03, 0.04) | 42.8(18.8-81.4) | 43.2(19.3-81.8) | 0.03(0.03, 0.04) |
| South Africa | 27198.7(18844.6-36380.2) | 26024.1(18467.7-34366.2) | -0.13(-0.14, -0.12) | 25.1(11-48.8) | 23.8(10.3-46.2) | -0.15(-0.17, -0.14) |
| South Sudan | 39013.2(26682.1-51146) | 41568.3(29448.8-54033.3) | 0.2(0.19, 0.21) | 35.6(15.6-69.1) | 37.8(16.7-70.8) | 0.19(0.18, 0.2) |
| Spain | 50138.8(37429.5-61845.2) | 48448.7(35629.5-60209.8) | -0.14(-0.17, -0.11) | 46.7(21-87.7) | 45.1(20-85.9) | -0.14(-0.17, -0.11) |
| Sri Lanka | 39801.3(27372.1-52611.5) | 36178.3(24514.5-48409.6) | -0.28(-0.3, -0.25) | 36.7(16.2-72.3) | 33(14.2-63.7) | -0.32(-0.33, -0.29) |
| Sudan | 46640.8(34276.3-58382.6) | 45944.3(33363.9-57969.5) | -0.05(-0.06, -0.05) | 43(18.7-81) | 42.4(18.7-81.9) | -0.05(-0.07, -0.04) |
| Suriname | 22591.6(14569.4-32597.3) | 21463.5(13834.1-31214.1) | -0.16(-0.17, -0.14) | 21(8.9-41.4) | 19.8(8.3-39.6) | -0.18(-0.2, -0.17) |
| Sweden | 32401.9(20825.4-44388.2) | 39688(28340.1-51225.6) | 0.66(0.63, 0.68) | 30.2(12.5-60.5) | 37(16.2-71.1) | 0.65(0.63, 0.67) |
| Switzerland | 61268.5(50244.7-70538.1) | 60818.1(49114.2-70058.9) | -0.02(-0.03, -0.02) | 56.8(26.4-104.4) | 56.4(25.8-104.7) | -0.02(-0.02, -0.02) |
| Syrian Arab Republic | 45400.9(33085.3-56994.3) | 45425.4(33192.7-57463.8) | -0.01(-0.03, 0) | 42(18.2-80.2) | 41.8(18.3-78.9) | -0.03(-0.06, -0.02) |
| Taiwan (Province of China) | 12092(7628.9-17698.5) | 11739.7(7346.9-17212.8) | -0.09(-0.12, -0.06) | 11.3(4.5-23.1) | 10.9(4.5-22) | -0.1(-0.14, -0.07) |
| Tajikistan | 38144.8(26090.7-51224.6) | 39688.1(27471.5-52403.1) | 0.13(0.11, 0.14) | 35.7(15.6-70) | 37.1(16-70.6) | 0.13(0.12, 0.14) |
| Thailand | 38202.2(25385.3-50956.2) | 35905.3(24212.7-48726.7) | -0.2(-0.2, -0.19) | 35.2(15.5-68.6) | 33.2(14.1-63.5) | -0.19(-0.2, -0.19) |
| Timor-Leste | 48751.5(35893.1-60972.3) | 45856.5(33459-57913.7) | -0.18(-0.19, -0.16) | 44.8(19.7-83.7) | 42.1(18.5-80.2) | -0.19(-0.21, -0.18) |
| Togo | 36239.7(24883.1-48225.9) | 20478.1(12789.6-29813.4) | -1.82(-1.87, -1.78) | 33.6(14.4-65.6) | 19.1(7.2-39) | -1.8(-1.84, -1.76) |
| Tokelau | 43929.2(31411.1-55835.8) | 42876.4(31025.8-54783.6) | -0.07(-0.09, -0.05) | 40.6(17.7-78.5) | 39.3(17.6-75.2) | -0.09(-0.12, -0.07) |
| Tonga | 44141.5(32217.5-55585.9) | 43563.8(31321.2-56022.7) | -0.04(-0.05, -0.04) | 40.9(18.1-77.1) | 40.2(17.8-77.6) | -0.05(-0.06, -0.05) |
| Trinidad and Tobago | 33641(22775.5-46175.5) | 22829(13898.8-34534.8) | -1.23(-1.26, -1.21) | 31.3(13.5-61.9) | 21.1(7.9-43.1) | -1.25(-1.27, -1.23) |
| Tunisia | 43937.7(31837.1-56253.4) | 43259(30830.4-54560) | -0.04(-0.05, -0.03) | 40.7(18.1-78.1) | 39.6(17.5-76.9) | -0.07(-0.08, -0.05) |
| Turkey | 46533.1(33737.7-58434.3) | 44279.4(31710.4-56046.1) | -0.15(-0.17, -0.14) | 43.2(18.6-82.9) | 40.8(18.5-76.9) | -0.17(-0.19, -0.16) |
| Turkmenistan | 36269.3(24800.5-48970.9) | 35129.3(23350.6-47978) | -0.11(-0.12, -0.09) | 33.9(14.5-65.1) | 32.8(13.9-64.5) | -0.11(-0.13, -0.1) |
| Tuvalu | 45739.4(33438.9-57526.2) | 44095.8(32392-55882.4) | -0.09(-0.11, -0.06) | 42.4(18.8-80.9) | 40.6(17.7-77.3) | -0.11(-0.13, -0.08) |
| Uganda | 25494.2(16221.6-36523) | 24191.3(15519.6-35114.9) | -0.19(-0.21, -0.16) | 23.5(9.6-47.1) | 22.4(9.2-45.1) | -0.17(-0.19, -0.15) |
| Ukraine | 26575.5(17419.8-37687.3) | 27485.6(17761.5-38630) | 0.11(0.1, 0.12) | 24.5(10.2-48.3) | 25.3(10.5-50.4) | 0.12(0.11, 0.12) |
| United Arab Emirates | 37074.5(25115.6-49234.7) | 38950.6(26979.9-51831.5) | 0.15(0.14, 0.16) | 34(14.9-66.1) | 35.7(15.2-68.7) | 0.15(0.14, 0.16) |
| United Kingdom | 43106.2(33710.9-51432) | 39304.5(31382.5-46775.4) | -0.3(-0.32, -0.28) | 40.2(18.4-76.8) | 36.5(16.6-70.8) | -0.31(-0.33, -0.3) |
| United Republic of Tanzania | 35210.3(24861.4-46145.3) | 33751.9(23516.9-44435.9) | -0.15(-0.16, -0.13) | 32.5(14-63.5) | 31.2(13.5-59.2) | -0.14(-0.16, -0.12) |
| United States of America | 19292.8(12344.3-27930.8) | 19458.3(13339.1-26964.6) | 0.02(0.02, 0.03) | 17.8(7.4-35.1) | 17.7(7.6-34.4) | -0.03(-0.04, -0.02) |
| United States Virgin Islands | 32653.8(21641.8-45279) | 30414.5(20367.9-43091.7) | -0.21(-0.22, -0.19) | 30.5(13.1-60) | 28.1(12-54.6) | -0.26(-0.27, -0.24) |
| Uruguay | 50642.3(38115.9-62036.9) | 50136.1(37337.6-62126) | -0.03(-0.04, -0.02) | 47.2(21.3-90.1) | 46.6(20.5-89.3) | -0.05(-0.06, -0.04) |
| Uzbekistan | 38196.9(26753.3-50972.2) | 37137.7(25660.8-49756.3) | -0.09(-0.1, -0.09) | 35.6(15.5-69.4) | 34.6(14.8-68.7) | -0.1(-0.11, -0.09) |
| Vanuatu | 45360.8(33174.9-56839.8) | 45232.1(33073.5-56776.6) | -0.01(-0.01, 0) | 42.1(18.6-78.8) | 41.8(18.5-79.9) | -0.02(-0.02, -0.01) |
| Venezuela (Bolivarian Republic of) | 49089.4(35519.7-62606.7) | 49297.3(35477-62976.6) | 0.01(0.01, 0.02) | 45.5(19.9-87.1) | 45.5(20.3-87.3) | 0.01(0, 0.01) |
| Viet Nam | 36887.7(25341.2-50217.7) | 31609.6(21250.8-43387.6) | -0.51(-0.57, -0.46) | 34.3(14.6-68) | 29.4(12.4-57.4) | -0.51(-0.54, -0.48) |
| Yemen | 39443.1(28225.6-50990.4) | 40305.6(28438.9-51492.4) | 0.07(0.06, 0.07) | 36.3(15.8-70.1) | 37.1(16.5-69.7) | 0.06(0.06, 0.07) |
| Zambia | 32203.6(24572.7-40156.3) | 21100.5(12935-30456.4) | -1.34(-1.38, -1.29) | 29.8(13.3-58.2) | 19.6(7.7-40.2) | -1.33(-1.36, -1.29) |
| Zimbabwe | 21373.4(13569.7-30630.2) | 47572.9(34242.6-59428.3) | 0.15(0.1, 0.18) | 42.8(19-82.3) | 42.8(19.1-81.8) | 0.12(0.07, 0.16) |

Data in parentheses are 95% confidence intervals.

ASR, age-standardized rate; AAPC, average annual percentage change; DALYs, disability-adjusted life years.

**Table S10 The prevalence and DALYs of periodontal diseases and their trends from 1990 to 2021 at the national level**

| Location | **Prevalence** | | | **DALYs** | | |
| --- | --- | --- | --- | --- | --- | --- |
|  | ASR in 1990 | ASR in 2021 | AAPC (1990-2021) | ASR in 1990 | ASR in 2021 | AAPC (1990-2021) |
| Afghanistan | 18032.7(11760.4-24957.1) | 17421.7(11379-23954.6) | -0.12(-0.14, -0.1) | 111.2(41.7-249.5) | 105.6(38.7-222.7) | -0.18(-0.2, -0.16) |
| Albania | 17097.9(11052.2-23516) | 20252(14042.5-26796.6) | 0.56(0.54, 0.57) | 107.1(40.3-236.8) | 126.6(46.4-268.4) | 0.55(0.54, 0.57) |
| Algeria | 23351(16708.3-29996) | 22943.5(16675.5-29457.3) | -0.05(-0.06, -0.04) | 145.5(55.7-311) | 141.8(52.2-292) | -0.07(-0.08, -0.05) |
| American Samoa | 28612.3(21907.4-35604.2) | 27704.5(20850.2-34688.1) | -0.1(-0.1, -0.1) | 178(68.8-371.4) | 170.2(66.1-352.6) | -0.14(-0.15, -0.14) |
| Andorra | 23273(16086.5-30463.8) | 22178.9(15259.8-29138.8) | -0.14(-0.16, -0.12) | 146.5(55.3-316.1) | 138.6(50.7-291.8) | -0.16(-0.17, -0.14) |
| Angola | 32839(25022.1-40239.3) | 27575.6(19290.8-35254.5) | -0.54(-0.57, -0.51) | 203.5(80-418.8) | 170.1(62.8-350.1) | -0.54(-0.57, -0.52) |
| Antigua and Barbuda | 26291.2(19929.9-32959.7) | 26465.8(19713.3-33110.8) | 0.02(0.01, 0.04) | 165.4(63.2-350.5) | 164.5(62.4-331.9) | -0.01(-0.03, 0) |
| Argentina | 22001.9(15241.4-29010.8) | 22687.3(15641.5-29701.3) | 0.13(0.08, 0.18) | 138.2(52.1-297.9) | 141.6(51.9-296.7) | 0.07(0.03, 0.11) |
| Armenia | 22307.6(15880.4-29077.3) | 17821.3(12158-24112.6) | -0.67(-0.73, -0.63) | 139.5(53.1-299) | 111.4(40.5-235.3) | -0.68(-0.72, -0.63) |
| Australia | 14439.3(9581.9-19283.9) | 17871.6(11646.9-24556.7) | 0.58(0.33, 0.93) | 89.8(33.4-192.5) | 111(39.9-236.4) | 0.71(0.57, 0.85) |
| Austria | 18778.6(12161.4-26001.2) | 19527.5(12933.8-26483.3) | 0.12(0.09, 0.16) | 117.3(44.2-260) | 122(43.9-259.7) | 0.12(0.09, 0.15) |
| Azerbaijan | 23763.9(17155.3-30568.8) | 19075.6(13254.5-25374.6) | -0.71(-0.76, -0.67) | 150(57.7-323.4) | 119.8(43.8-250.6) | -0.73(-0.78, -0.69) |
| Bahamas | 27793.9(21467.9-34277.9) | 26965.6(20418.8-33639.1) | -0.09(-0.1, -0.09) | 175.4(67.8-365) | 168.6(63.1-344.4) | -0.12(-0.13, -0.12) |
| Bahrain | 25963.6(19716-32575.3) | 25947.7(19610.3-32424.7) | 0(-0.01, 0.01) | 160.6(62.5-334.6) | 158.3(60.1-322.4) | -0.04(-0.05, -0.04) |
| Bangladesh | 32633.1(24262.3-40528.1) | 36724.7(27838.8-44744) | 0.37(0.36, 0.38) | 202.5(78.2-426.3) | 228.1(86.5-461.2) | 0.37(0.36, 0.38) |
| Barbados | 26404.1(20069.7-33204.5) | 25283.3(18584.2-31835.2) | -0.15(-0.15, -0.14) | 166.7(63.9-349.9) | 157.9(59-321.1) | -0.18(-0.19, -0.17) |
| Belarus | 24665.6(17908-31582) | 24164.3(17307.2-31096) | -0.07(-0.08, -0.06) | 153.9(59.4-328.2) | 150.1(55.4-309.5) | -0.08(-0.09, -0.07) |
| Belgium | 24386.1(18358.5-30857.8) | 25459.4(19453.3-31724.2) | 0.12(0.08, 0.18) | 152.7(58.7-321.9) | 158.8(59.6-320.7) | 0.12(0.07, 0.18) |
| Belize | 23814.6(17379-30725.6) | 23748.8(17138.3-30526.8) | -0.02(-0.04, -0.01) | 150.8(58-321.5) | 148.6(55.1-307.3) | -0.05(-0.07, -0.03) |
| Benin | 35793.1(28094.3-43396.4) | 31431.2(23586.8-38878.4) | -0.41(-0.44, -0.39) | 221.7(87.1-460.6) | 195.1(73.5-399.3) | -0.41(-0.43, -0.38) |
| Bermuda | 28435.4(21903-35200.8) | 29991.2(23625.4-36825.6) | 0.17(0.17, 0.17) | 179.6(69-370.3) | 188.2(72.9-389.3) | 0.15(0.15, 0.15) |
| Bhutan | 31812.9(23997.4-39219.1) | 36985.8(28953.4-44555.4) | 0.49(0.48, 0.49) | 198.6(77.8-414.1) | 230.4(88.1-460.4) | 0.48(0.48, 0.48) |
| Bolivia (Plurinational State of) | 14428.1(9739.4-20027.7) | 13481.4(9136.6-18924.1) | -0.21(-0.23, -0.17) | 90.4(34.3-202.8) | 83.9(30.7-179.8) | -0.23(-0.25, -0.19) |
| Bosnia and Herzegovina | 14498.8(9005.1-20879.7) | 20031.9(13670.4-26722.1) | 1.06(0.93, 1.18) | 90.2(32.7-200.6) | 123.7(45.1-262.2) | 1.04(0.93, 1.14) |
| Botswana | 16438.2(9882.2-24207.3) | 12502.5(7317.4-18871.7) | -0.85(-0.89, -0.8) | 102.5(36.1-231.7) | 76.8(25.1-169.1) | -0.89(-0.93, -0.84) |
| Brazil | 15962.2(11340.3-21666.1) | 16659.8(12629.8-21269.9) | 0.14(0.11, 0.17) | 98.7(37.9-215.4) | 103(40-215.9) | 0.14(0.1, 0.18) |
| Brunei Darussalam | 28398.8(20769-35964.8) | 24935.9(17170.6-32529.2) | -0.43(-0.44, -0.42) | 176.8(67.9-375.9) | 154.4(55.7-323.6) | -0.44(-0.46, -0.43) |
| Bulgaria | 17600.4(11081.3-24607.4) | 18737.2(12013.4-25618.8) | 0.22(0.16, 0.28) | 109.8(40.4-245.3) | 116.3(40.9-249.6) | 0.19(0.14, 0.24) |
| Burkina Faso | 36397.5(28030.9-44248.7) | 37263.7(28655.5-45350.4) | 0.07(0.07, 0.08) | 226.1(87.1-469) | 233.1(89.9-487) | 0.1(0.09, 0.1) |
| Burundi | 34848.2(26879.1-42569.2) | 22776.4(14183.5-31658.1) | -1.36(-1.41, -1.31) | 217.5(85-455.1) | 141.9(50-303.5) | -1.38(-1.43, -1.32) |
| Cabo Verde | 36484.5(28802-43969.8) | 38931.9(31469.3-46269.2) | 0.21(0.21, 0.21) | 229.8(89.5-481.3) | 243(94.6-497.4) | 0.18(0.18, 0.18) |
| Cambodia | 20310.8(13259.6-27703.9) | 24064.3(16712-31295.1) | 0.55(0.54, 0.55) | 125.8(46.5-272.9) | 149.1(56.1-319.3) | 0.55(0.54, 0.55) |
| Cameroon | 37160(29546.8-44718.5) | 37080(29309.4-44616.3) | -0.01(-0.01, 0) | 230.7(90.1-473.5) | 231.1(89.3-479.7) | 0(0, 0.01) |
| Canada | 33713.5(27209.4-40564.2) | 31217.2(24704.9-37488.6) | -0.24(-0.25, -0.22) | 211.6(82.4-443) | 194.5(75.8-390.4) | -0.28(-0.29, -0.26) |
| Central African Republic | 30064.8(22153.7-37748.5) | 20603.1(12694.9-28936.8) | -1.23(-1.26, -1.19) | 184.6(72.2-390.6) | 126.2(43.2-275.2) | -1.23(-1.26, -1.2) |
| Chad | 33740(25733.4-41389.7) | 29878.9(22193.3-37222.7) | -0.4(-0.43, -0.37) | 209.8(82.1-437.4) | 185.6(69.6-377.8) | -0.41(-0.43, -0.38) |
| Chile | 22521(15770.4-29483.3) | 25292.3(18488.2-32043.7) | 0.34(0.29, 0.38) | 140.8(53.3-301.7) | 157.1(59-324) | 0.36(0.31, 0.4) |
| China | 25635.4(18813.6-32797.8) | 25977.6(20798.5-31245) | 0.05(-0.02, 0.12) | 161(61.5-343.2) | 162.7(62.8-337.6) | 0.04(-0.03, 0.1) |
| Colombia | 25255.4(18718.5-31918.4) | 26599.2(19875.5-33400.7) | 0.16(0.15, 0.18) | 157.8(61-335.4) | 166(63.2-337.9) | 0.16(0.15, 0.17) |
| Comoros | 36482(28810.1-43872.1) | 26501.9(17505.8-34453.9) | -1.02(-1.08, -0.97) | 227.2(89.1-472.1) | 164.1(58.2-342.9) | -1.04(-1.07, -1) |
| Congo | 33319.9(25762.2-40658.1) | 26891.1(18459.2-34653.2) | -0.68(-0.72, -0.65) | 206.9(81.3-428.8) | 166(60.5-346.5) | -0.69(-0.72, -0.66) |
| Cook Islands | 27470.8(20627.2-34430.7) | 10028.5(5637.9-15512.1) | -3.2(-3.44, -3.03) | 171.1(65.7-357.7) | 61.6(20.4-138.8) | -3.25(-3.41, -3.15) |
| Costa Rica | 24661.9(18090.3-31346.2) | 25570.4(18930.9-32268.6) | 0.11(0.1, 0.12) | 154.5(59.7-323.4) | 159(59.9-325.9) | 0.09(0.08, 0.1) |
| Coted'Ivoire | 35697.7(28530.5-42933.9) | 31260.1(23747.4-38401.8) | -0.43(-0.45, -0.41) | 219(85.2-455.5) | 194.3(72.7-393.6) | -0.39(-0.41, -0.38) |
| Croatia | 26677.3(21279.7-32221.4) | 25921.4(19746.4-32197.3) | -0.07(-0.12, -0.02) | 165.4(65.7-340.9) | 160.6(60.8-324.6) | -0.06(-0.11, -0.02) |
| Cuba | 24708.7(18187.9-31463.5) | 23461.9(16971.5-30208.2) | -0.17(-0.19, -0.15) | 155.8(60.6-330) | 146.7(54.3-301) | -0.2(-0.22, -0.18) |
| Cyprus | 19659(12734.5-27171.7) | 19734.1(12773.7-26846) | -0.01(-0.03, 0.01) | 122.9(45.5-273.4) | 123.3(43.9-264.9) | -0.01(-0.02, 0.01) |
| Czechia | 20883.9(14629.9-27538.9) | 22702.5(16322.3-29319.5) | 0.29(0.25, 0.33) | 129(49-278.3) | 139.9(51.4-287.1) | 0.29(0.25, 0.33) |
| Democratic People's Republic of Korea | 23298.3(15979.1-30760.7) | 20022.9(12919.8-27717.8) | -0.49(-0.49, -0.48) | 147.2(54.3-317.3) | 126.5(46.6-278.9) | -0.48(-0.49, -0.48) |
| Democratic Republic of the Congo | 31321(23586.4-38542.6) | 21238.2(13304.7-29642.2) | -1.24(-1.3, -1.19) | 192.7(75.6-403.1) | 130.9(45.6-283) | -1.25(-1.3, -1.21) |
| Denmark | 31582.6(25417.8-38012.2) | 32888.1(26791.3-39011.2) | 0.13(0.08, 0.17) | 198(77.3-410.2) | 207.1(80.7-417.2) | 0.17(0.12, 0.21) |
| Djibouti | 37777.7(30151.4-45126.9) | 28432.9(19315.1-36827.6) | -0.9(-0.94, -0.86) | 236.3(91.8-485.6) | 176.7(64.4-369.5) | -0.93(-0.96, -0.9) |
| Dominica | 24241.8(17687.6-30893.1) | 24339.9(17801.4-31167.3) | 0(-0.01, 0.02) | 152.8(58.8-323.3) | 151.8(56.9-313.3) | -0.03(-0.04, -0.02) |
| Dominican Republic | 22600.3(16289-29393.1) | 23231.4(17009.3-29923.4) | 0.08(0.06, 0.1) | 142.8(55-304.2) | 145.3(54.3-300.5) | 0.05(0.02, 0.07) |
| Ecuador | 15974(12453.7-20054) | 16015.7(11062.6-22015.3) | 0.05(-0.01, 0.11) | 100.4(38.9-207.1) | 99.9(37-209.9) | 0.03(-0.03, 0.1) |
| Egypt | 20739.6(14081.1-27620.1) | 22184.5(15853-28679.6) | 0.2(0.19, 0.22) | 129.4(48.9-279.9) | 137(51.1-283.5) | 0.17(0.16, 0.18) |
| El Salvador | 22418(15941.1-29251.8) | 23547.4(16808.6-30365.2) | 0.16(0.15, 0.16) | 140.2(53.4-300.5) | 146.6(54.4-303.2) | 0.14(0.14, 0.15) |
| Equatorial Guinea | 31406.2(23603.6-38696.5) | 30856.4(22975-38295.4) | -0.05(-0.09, -0.01) | 192(74.8-401.6) | 189.6(71.6-389) | -0.03(-0.06, 0) |
| Eritrea | 34767.6(26721.8-42644.9) | 25485.3(16549.3-34133) | -1.03(-1.07, -0.98) | 215.4(84-449.8) | 157.2(56-335.3) | -1.07(-1.11, -1.02) |
| Estonia | 24551.5(17686.7-31345.5) | 25528.6(18888.5-32404.6) | 0.14(0.11, 0.17) | 153.2(58.8-324.3) | 159(58.6-328.5) | 0.13(0.11, 0.16) |
| Eswatini | 16504.6(9925.6-24537.9) | 18752.7(11476.9-27066.7) | 0.41(0.4, 0.42) | 103.1(36-236.3) | 115.6(41.6-259.3) | 0.37(0.36, 0.38) |
| Ethiopia | 33443.2(25375.7-41210) | 34140.9(26371.9-41480) | 0.07(0.06, 0.07) | 205.3(80.2-432.1) | 211.3(83.1-438.7) | 0.1(0.09, 0.11) |
| Fiji | 25043.3(17894.3-32371.2) | 7477.7(4151.9-11827.3) | -3.77(-3.9, -3.65) | 155.3(59.2-328.5) | 45.7(14.9-104.1) | -3.8(-3.92, -3.68) |
| Finland | 16936.5(11491.9-22923.8) | 25085.7(18799.5-31412) | 1.3(1.21, 1.37) | 105.5(39.1-229.4) | 156.3(58.7-318.9) | 1.29(1.23, 1.35) |
| France | 15198.3(10469.5-20283.5) | 17653.7(10853.2-24989.8) | 0.57(0.48, 0.66) | 94.4(33.5-200.8) | 109.8(38.5-238.9) | 0.59(0.51, 0.68) |
| Gabon | 36012.8(28957.3-42915.9) | 29931.5(21852.8-37441.5) | -0.6(-0.62, -0.58) | 223.6(86.9-460.5) | 184.3(68.9-380.8) | -0.62(-0.64, -0.6) |
| Gambia | 39179.5(32201.5-46266.1) | 39198.5(32014.1-46446.8) | 0.01(-0.01, 0.03) | 245(95.1-501.3) | 243.8(94.8-500.9) | -0.01(-0.02, 0.01) |
| Georgia | 23629.6(17181.3-30318.3) | 19200.3(13224.2-25615.5) | -0.66(-0.68, -0.64) | 148.5(57.2-319.1) | 119.6(44-248.8) | -0.68(-0.7, -0.67) |
| Germany | 27174.6(21346.7-33475.7) | 29182.1(22978.5-35595.7) | 0.22(0.15, 0.3) | 170.2(65.9-354.1) | 182(70.8-366.1) | 0.21(0.16, 0.27) |
| Ghana | 36451.9(29003.4-44087.5) | 37943(30496.7-45333.6) | 0.14(0.13, 0.14) | 228(88.4-476.5) | 237.7(92.4-485.8) | 0.14(0.13, 0.15) |
| Greece | 17313.9(10851.4-24512) | 19324(12573.2-26395.5) | 0.34(0.29, 0.4) | 108.8(39.8-244.3) | 121.2(43.8-260.6) | 0.35(0.29, 0.4) |
| Greenland | 28178.6(20748.4-35461.6) | 28031.9(20958.4-34968.5) | -0.02(-0.04, -0.01) | 175(67.2-370) | 174.5(64.9-358) | -0.02(-0.03, 0) |
| Grenada | 23855.4(17286.8-30881.3) | 24958.9(18081.7-31771.8) | 0.14(0.12, 0.15) | 150(58-319.6) | 155.1(58.2-318.3) | 0.1(0.09, 0.12) |
| Guam | 30276.9(23874.7-37036.6) | 11117.2(6266.1-17261.9) | -3.23(-3.41, -3.13) | 190.8(74.4-396.8) | 69.5(22.9-158.1) | -3.25(-3.35, -3.17) |
| Guatemala | 22530.9(15892.6-29343) | 22541.8(16116.1-29158.2) | 0(-0.02, 0.02) | 140.2(54.4-300.9) | 139.2(51.6-288.4) | -0.03(-0.05, -0.01) |
| Guinea | 35991(28424.1-43735.7) | 36035(28119.2-43700.2) | 0.01(0, 0.01) | 224.5(88-467.1) | 225(87.7-467.7) | 0.01(0.01, 0.02) |
| Guinea-Bissau | 35317.4(27229.7-43002.8) | 30276.2(22190.3-38014) | -0.49(-0.52, -0.47) | 219.4(85.5-459.5) | 188.1(69.9-383.6) | -0.5(-0.51, -0.48) |
| Guyana | 22680.6(16096.3-29465.4) | 23755.6(17099.7-30619.4) | 0.14(0.13, 0.16) | 140.9(53.6-302.4) | 146.4(54.4-301.6) | 0.11(0.09, 0.12) |
| Haiti | 21194.4(14515-28130.4) | 19016.9(12515.3-25824.7) | -0.33(-0.35, -0.32) | 132.4(50-286.7) | 117.7(42.5-250) | -0.36(-0.38, -0.35) |
| Honduras | 22148.9(15474.6-28950.2) | 22281.7(15512.4-29098) | 0.01(0, 0.02) | 138.9(52.3-298.4) | 138.1(51-289) | -0.02(-0.03, -0.01) |
| Hungary | 10949.1(6223.5-17079.1) | 12677.1(7365.5-19260.9) | 0.48(0.33, 0.64) | 67.3(23.7-155.8) | 78.6(26-177.3) | 0.51(0.39, 0.63) |
| Iceland | 20082.2(14951.8-25171.6) | 20790.5(13781.1-27977.8) | 0.11(0.1, 0.12) | 126.3(47.1-263.5) | 130.7(47.5-280.4) | 0.11(0.1, 0.12) |
| India | 27670(20359.3-34857.2) | 29977(24336.3-35625.7) | 0.28(0.23, 0.35) | 168.6(65.8-359.1) | 183.3(70.9-369.6) | 0.3(0.24, 0.36) |
| Indonesia | 26036.9(19282.1-32945.4) | 27412.1(20441.2-34458.5) | 0.17(0.16, 0.17) | 162(62.5-344.8) | 170.6(66.1-361.7) | 0.17(0.17, 0.17) |
| Iran (Islamic Republic of) | 25289.4(18772.9-32066.8) | 25726.9(20324.8-31059) | 0.06(0.03, 0.09) | 156.9(60.6-331.9) | 158.8(61.9-329.4) | 0.05(0.02, 0.07) |
| Iraq | 23138.9(16581.9-29979.1) | 23222.6(16856.6-29835) | 0.04(0.02, 0.05) | 143.7(55-310) | 142(52.7-290.1) | -0.03(-0.05, -0.01) |
| Ireland | 9615.2(5552.4-15056.6) | 12826.5(7492.6-19028.5) | 0.9(0.82, 0.98) | 60.3(20.9-139.5) | 80.3(27.2-181.1) | 0.9(0.83, 0.97) |
| Israel | 19632.3(12704-27145.7) | 18718.1(12086-25818.3) | -0.14(-0.16, -0.11) | 123.6(45.7-276.2) | 117.3(42-254.3) | -0.16(-0.18, -0.14) |
| Italy | 22677.5(15588-29811.3) | 22018.8(15115.6-28990.6) | -0.1(-0.14, -0.07) | 141.1(52.9-307.8) | 137.2(49.6-289.7) | -0.1(-0.13, -0.07) |
| Jamaica | 24329.9(17758.5-31112.5) | 23785.4(17161-30528.1) | -0.07(-0.08, -0.06) | 153.8(59.1-328.9) | 148.9(55-309.5) | -0.1(-0.11, -0.09) |
| Japan | 21555.7(14369.4-28940.2) | 24243.5(16892-31353.4) | 0.38(0.26, 0.51) | 135.7(50.1-294.8) | 152.7(55.6-319.9) | 0.37(0.28, 0.46) |
| Jordan | 22170.9(15704.3-28828.6) | 21960(15542.9-28577.9) | -0.03(-0.05, -0.02) | 137.9(52.4-294.7) | 135.2(49.7-281.4) | -0.06(-0.08, -0.05) |
| Kazakhstan | 22087.9(15866.9-28732) | 19057.3(13465.4-25468.2) | -0.47(-0.5, -0.44) | 137.9(52.6-296.6) | 118.4(43.4-242.5) | -0.48(-0.51, -0.46) |
| Kenya | 35847.8(28023.8-43295.3) | 34807.1(26956.2-42096.4) | -0.09(-0.1, -0.09) | 222.3(87.2-462.5) | 215.2(81.8-436.2) | -0.1(-0.1, -0.1) |
| Kiribati | 22136.9(14952-29418.2) | 4960.4(2736.1-7831.5) | -4.66(-4.79, -4.55) | 136.7(51.3-299) | 30.4(9.7-69.9) | -4.69(-4.81, -4.58) |
| Kuwait | 26982.7(20628.3-33471.2) | 26680.3(20373.2-33010.3) | -0.04(-0.05, -0.03) | 168.3(64.6-348.5) | 164.4(62.1-331) | -0.08(-0.09, -0.07) |
| Kyrgyzstan | 22303.8(15838.2-28981.5) | 15618(10105.2-21976.7) | -1.14(-1.2, -1.1) | 139.9(53.5-299.3) | 98.1(35.9-210.7) | -1.15(-1.23, -1.1) |
| Lao People's Democratic Republic | 12201.9(6994.6-18798.9) | 17225.5(10330.2-25427.6) | 1.14(1.09, 1.2) | 76.2(25.9-176.1) | 107.6(37.8-243.3) | 1.14(1.1, 1.2) |
| Latvia | 26120.5(19174.6-33070.5) | 26529.5(19708.2-33477.8) | 0.07(0.04, 0.09) | 162.1(63.1-340.7) | 164.6(61-336.4) | 0.07(0.04, 0.09) |
| Lebanon | 23186.5(16556.7-29852.4) | 22949(16551.4-29660.4) | -0.05(-0.06, -0.03) | 143.5(56-309.8) | 140.7(52.8-287.6) | -0.08(-0.09, -0.07) |
| Lesotho | 11755.8(6833.1-18152.4) | 14523(8585.4-21921) | 0.68(0.67, 0.69) | 73.5(25.9-169.5) | 89.3(31.7-203.2) | 0.63(0.62, 0.64) |
| Liberia | 35485.4(27565.8-43135.6) | 34478.1(26343.8-42435.6) | -0.09(-0.1, -0.08) | 219(85-454.6) | 212.5(83.3-445.4) | -0.1(-0.1, -0.09) |
| Libya | 26141.2(19850.4-32767) | 21979.8(15571.4-28560.4) | -0.55(-0.57, -0.53) | 163.2(62.4-342.6) | 135.1(49.5-279) | -0.6(-0.62, -0.59) |
| Lithuania | 26555.5(19723.6-33532.9) | 26888.2(19936.2-33804.3) | 0.04(0.01, 0.07) | 165.3(64.4-346.8) | 166.7(62.9-340.4) | 0.03(0, 0.06) |
| Luxembourg | 20885.6(13991.9-28101.7) | 21313.7(14405-28281.1) | 0.05(0.01, 0.1) | 130.8(49.3-287) | 133.4(49-280.5) | 0.05(0.01, 0.09) |
| Madagascar | 35820.6(27715.1-43488.7) | 11457.1(6647.4-17443.5) | -3.65(-3.8, -3.53) | 223.2(87.7-465.8) | 71(23.8-158.2) | -3.66(-3.76, -3.53) |
| Malawi | 34581.7(26537.4-42436.3) | 24043.2(15537.7-32859.3) | -1.15(-1.23, -1.09) | 215(83.2-450.4) | 149.1(52.2-314.4) | -1.15(-1.2, -1.1) |
| Malaysia | 22195.8(15958.8-28849.4) | 21871.3(15499.5-28714.4) | -0.12(-0.21, -0.02) | 138.1(52.5-297.2) | 135.3(50.4-286.4) | -0.13(-0.22, -0.03) |
| Maldives | 25748.3(18293.6-32929.1) | 24145.5(16933.4-31146.3) | -0.2(-0.23, -0.18) | 161.2(60.3-345.7) | 150.5(54.6-315.8) | -0.22(-0.25, -0.19) |
| Mali | 35808.3(27486.2-43492.1) | 37274.9(29069.9-45257.9) | 0.13(0.12, 0.14) | 221.9(85.9-460.7) | 231.3(89.7-478.4) | 0.14(0.13, 0.14) |
| Malta | 20475.5(13250.6-28106.5) | 21389.5(14005.7-28814.7) | 0.14(0.11, 0.17) | 129.1(47.6-287.2) | 134.2(47.8-285.2) | 0.12(0.09, 0.15) |
| Marshall Islands | 23052(15833-30242.9) | 5798.9(3226.2-9075.8) | -4.3(-4.46, -4.15) | 143.2(53.7-305.5) | 35.5(11.5-80.9) | -4.38(-4.5, -4.26) |
| Mauritania | 35300.2(28007.6-42800) | 31173.6(23625.1-38321.5) | -0.4(-0.43, -0.38) | 220.9(86.2-461.1) | 195.4(73.2-395.5) | -0.4(-0.42, -0.37) |
| Mauritius | 21692.5(15419.1-28374.9) | 20865(14604.8-27419.9) | -0.12(-0.17, -0.07) | 135(51.6-293.2) | 128.2(47.2-268.4) | -0.16(-0.21, -0.12) |
| Mexico | 23833.4(17649.7-30413.2) | 23744.5(19163.1-28709.3) | -0.01(-0.03, 0) | 147.3(56.9-313.7) | 147.3(56.8-299.3) | 0(-0.02, 0.01) |
| Micronesia (Federated States of) | 22825.7(15652-30037.9) | 5690.8(3140.1-9010.5) | -4.41(-4.61, -4.25) | 142.1(53-307) | 35(11.3-79.6) | -4.44(-4.57, -4.31) |
| Monaco | 26526.1(19562.2-33470) | 26230.6(19287.7-33219.9) | -0.03(-0.04, -0.01) | 167.9(65.1-355.2) | 165(60.8-338.6) | -0.04(-0.05, -0.03) |
| Mongolia | 20796.1(14219.1-27373.5) | 18242.5(12593.8-24576.6) | -0.42(-0.45, -0.38) | 130.6(49.4-281.7) | 114.1(41.9-240.8) | -0.43(-0.47, -0.4) |
| Montenegro | 19957.7(13848.3-26521.1) | 20924(14594.1-27415.6) | 0.16(0.14, 0.18) | 124.9(46.5-271.7) | 129.9(47.6-272.6) | 0.13(0.11, 0.14) |
| Morocco | 24401(18590.8-30800.5) | 23743.2(17698.3-29938.1) | -0.08(-0.1, -0.06) | 152.4(59.1-317.6) | 146.1(55.3-297.3) | -0.13(-0.14, -0.11) |
| Mozambique | 32964.5(24767.5-41005.2) | 24632.7(15874.8-33363.8) | -0.92(-0.99, -0.86) | 203(79-427.5) | 151.2(53.2-320.9) | -0.92(-0.96, -0.88) |
| Myanmar | 23200.7(15299.1-31138.5) | 23713.4(16038-31421.4) | 0.08(0.06, 0.1) | 143.9(52.5-311.3) | 146.1(53.2-310.6) | 0.06(0.04, 0.08) |
| Namibia | 15804.1(9419.7-23296.4) | 18092.4(10998.1-26399.6) | 0.48(0.44, 0.52) | 98.9(35.3-224.7) | 112.8(40-252.4) | 0.47(0.43, 0.51) |
| Nauru | 27815.6(21183.8-34652.5) | 7777.6(4326.8-12255.1) | -4.04(-4.17, -3.91) | 173.3(66.9-362.6) | 47.7(15.8-108.6) | -4.06(-4.18, -3.93) |
| Nepal | 23841.1(15138.5-32801.8) | 26931.9(17597-36235.5) | 0.41(0.35, 0.46) | 146.4(52.9-316.8) | 165.4(59-347.2) | 0.38(0.35, 0.42) |
| Netherlands | 16874.1(10898.6-23534.1) | 17925.7(11564.1-24604.5) | 0.22(0.17, 0.26) | 106.2(39.8-237) | 112.2(40.5-236.8) | 0.19(0.14, 0.24) |
| New Zealand | 17672.2(11304-24560) | 20842.1(14950.3-27204.4) | 0.5(0.47, 0.53) | 109.4(41-246.1) | 129.6(48.1-264.8) | 0.52(0.49, 0.55) |
| Nicaragua | 22401.1(15860-29115.3) | 22546.3(15889.8-29426.2) | 0.03(0.02, 0.04) | 139.9(53.6-300.8) | 139.8(51.8-288.5) | 0(-0.01, 0.01) |
| Niger | 34318(26355.5-42122.1) | 16245.1(9706.2-24380.1) | -2.35(-2.49, -2.24) | 213.5(83.1-447.3) | 100.9(33.4-221.2) | -2.38(-2.47, -2.31) |
| Nigeria | 35433.6(27658.5-42913.4) | 13852(8056-20775.7) | -2.92(-3.04, -2.84) | 219(85.8-455.8) | 85.5(28.4-190.3) | -2.94(-3.06, -2.84) |
| Niue | 25907.4(18830.7-32978.8) | 8000.7(4486.3-12506.7) | -3.67(-3.84, -3.53) | 161.5(62.1-341.9) | 49.1(16.2-111.6) | -3.71(-3.83, -3.58) |
| North Macedonia | 18469.2(12478.2-24974) | 19851.5(13481.9-26495.1) | 0.24(0.22, 0.25) | 114.9(43-250.5) | 122.7(44.6-261.4) | 0.21(0.2, 0.22) |
| Northern Mariana Islands | 30254.9(23900.7-37193.4) | 9709.1(5403.8-15113.8) | -3.6(-3.79, -3.46) | 189.7(73.6-393.4) | 60.2(19.3-136.1) | -3.64(-3.74, -3.54) |
| Norway | 26114.5(19792.5-32666) | 25965.2(19746.4-32387.6) | 0.03(-0.12, 0.16) | 162.6(63.2-344.3) | 162.5(62-329.8) | 0.05(-0.07, 0.15) |
| Oman | 25328.4(18589.7-31979.4) | 25668.3(19200.2-32235.7) | 0.05(0.04, 0.06) | 156.3(59.8-326.3) | 157.8(59.6-319.3) | 0.04(0.03, 0.05) |
| Pakistan | 32056.1(25038.3-39112.9) | 35026.5(27624.9-41897.1) | 0.29(0.25, 0.33) | 198.6(77.7-416.7) | 215.5(83.2-428.3) | 0.27(0.24, 0.3) |
| Palau | 26638.8(19537.5-33672.3) | 8223.6(4622.1-12823.2) | -3.74(-3.9, -3.6) | 165.8(64-351.9) | 50.4(16.3-114.9) | -3.78(-3.89, -3.66) |
| Palestine | 19598.5(13234.5-26394.7) | 20052.9(13557.1-26749.4) | 0.08(0.06, 0.09) | 122(46.1-267.8) | 123.4(45.2-257.7) | 0.04(0.03, 0.06) |
| Panama | 24706.1(18054.5-31517) | 26801.2(20209.1-33506.7) | 0.27(0.26, 0.27) | 154.9(59.5-325.4) | 167(64.3-338.2) | 0.24(0.24, 0.25) |
| Papua New Guinea | 22722.8(15620.7-29907.3) | 5763.9(3215.1-9170.5) | -4.33(-4.53, -4.15) | 140.9(52.2-303.7) | 35.5(11.5-83.3) | -4.33(-4.47, -4.19) |
| Paraguay | 21832(15688.9-28573.5) | 20324.8(14239.6-26925.5) | -0.21(-0.26, -0.17) | 136.8(52.4-292.5) | 126.4(46.7-262.2) | -0.2(-0.24, -0.15) |
| Peru | 13407.4(10245.6-16999.7) | 13343.2(9277.8-18427.4) | -0.01(-0.03, 0.02) | 84.5(32.5-178.4) | 83.9(30.9-178.5) | -0.01(-0.04, 0.01) |
| Philippines | 22098.7(15848-28911) | 10398.6(7225.2-14322.2) | -2.39(-2.45, -2.34) | 136.9(52.5-293.7) | 64.3(23.6-139) | -2.4(-2.45, -2.35) |
| Poland | 19135.1(13540.7-25532) | 20389(14713.3-26672.3) | 0.12(-0.06, 0.26) | 118.1(45.5-255.3) | 126.3(46.6-258.7) | 0.17(0.03, 0.3) |
| Portugal | 18699.6(11886.8-25895.4) | 19092.3(12380.3-26039.4) | 0.05(0.02, 0.08) | 117(42.7-259.7) | 119.5(43-255.9) | 0.05(0.02, 0.08) |
| Puerto Rico | 27472.2(21108.3-34129.2) | 27871.4(21327.4-34530.8) | 0.04(0.02, 0.07) | 172.7(66.7-361.6) | 173.6(66.7-348) | 0.01(-0.01, 0.04) |
| Qatar | 31530.4(25219.9-38020.8) | 28356.5(21862-34515.7) | -0.34(-0.36, -0.32) | 194.6(75.5-406.1) | 172.7(66.1-347.2) | -0.38(-0.4, -0.36) |
| Republic of Korea | 21018.1(13547.2-28916.7) | 23461.9(15443-31052.4) | 0.33(0.27, 0.36) | 131.2(48-290.2) | 146.5(51.9-314.4) | 0.33(0.27, 0.37) |
| Republic of Moldova | 22622.3(15825-29677.4) | 21217.3(14181.1-28154.8) | -0.19(-0.21, -0.17) | 141.1(53.6-304.7) | 132(47.7-279.9) | -0.2(-0.21, -0.18) |
| Romania | 20432.3(13831-27198.5) | 22683.9(15984.3-29476.5) | 0.35(0.31, 0.38) | 127.5(47.6-277) | 141.5(52.7-292.5) | 0.33(0.29, 0.36) |
| Russian Federation | 23391(17134.3-30101.5) | 21592.3(15664-27957.8) | -0.26(-0.27, -0.25) | 145.2(55.8-310.7) | 133.4(49.6-273.7) | -0.27(-0.28, -0.26) |
| Rwanda | 34867.6(26828.2-42611.9) | 26146.1(17431.9-34638.1) | -0.9(-0.95, -0.86) | 217.5(84.6-454.3) | 162.3(58.4-344.5) | -0.91(-0.95, -0.87) |
| Saint Kitts and Nevis | 25790.5(19338.1-32481.3) | 26667.4(20188.1-33366.3) | 0.11(0.11, 0.12) | 161.7(62.1-337.6) | 165.9(62.3-338.3) | 0.08(0.08, 0.09) |
| Saint Lucia | 24792.7(18222.5-31715.5) | 24879(18213.3-31516.9) | 0.01(-0.01, 0.02) | 155.1(59-326.2) | 154.5(58-313.1) | -0.02(-0.04, -0.01) |
| Saint Vincent and the Grenadines | 23834.4(17347.3-30553.7) | 24632.9(17879.3-31420) | 0.1(0.09, 0.12) | 149.5(57.3-317.6) | 153(56.8-314.7) | 0.07(0.06, 0.09) |
| Samoa | 23962.4(16976.9-31108.5) | 6519.6(3581.2-10227.9) | -4.11(-4.29, -3.96) | 149.3(56.4-319.9) | 40.1(13.1-90.8) | -4.13(-4.25, -4.01) |
| San Marino | 22549.3(15570.6-29812.7) | 21273.3(14203.1-28319.8) | -0.2(-0.22, -0.18) | 141.9(53.3-309.3) | 133.3(48.1-282.7) | -0.21(-0.22, -0.19) |
| Sao Tome and Principe | 36290.2(28609.2-43758.1) | 32383.4(24596.5-39561.4) | -0.37(-0.41, -0.35) | 227.5(87.6-470.2) | 201.7(75.7-406.1) | -0.39(-0.41, -0.37) |
| Saudi Arabia | 19167.1(12871.3-25681.4) | 19619.5(12475-26772.3) | 0.06(0.02, 0.09) | 117.5(44.4-254.3) | 119.6(43.1-255) | 0.06(0.03, 0.1) |
| Senegal | 35369.7(27912-42679.6) | 30872.9(23276.5-37843.1) | -0.43(-0.45, -0.41) | 220.2(85.5-455.8) | 191.5(72.1-392.3) | -0.44(-0.46, -0.42) |
| Serbia | 19548.6(13323.1-26152.9) | 20632.2(14460.4-27435.4) | 0.17(0.16, 0.19) | 122.3(45.3-265.1) | 128(46.8-267.9) | 0.15(0.14, 0.16) |
| Seychelles | 27488.1(20519.5-34320) | 25405.8(18211.7-32215.8) | -0.25(-0.27, -0.23) | 172.2(66.3-359.3) | 156.3(57.8-323.1) | -0.31(-0.34, -0.29) |
| Sierra Leone | 25398.8(16367.6-34545.3) | 38758.3(32362.4-45239.4) | 1.3(1.22, 1.39) | 157.9(57.9-339.5) | 241.2(95.4-479.3) | 1.36(1.27, 1.43) |
| Singapore | 24191.4(16488.7-32019.1) | 25339.5(17546.3-32961.7) | 0.14(0.13, 0.15) | 152.8(56.2-331.7) | 160.3(58.4-335.3) | 0.14(0.13, 0.15) |
| Slovakia | 19152.7(13054.3-25791.6) | 21872.2(15560.9-28432.8) | 0.44(0.41, 0.46) | 118.6(44.8-257.2) | 135.6(50.3-284.8) | 0.44(0.42, 0.46) |
| Slovenia | 23838.2(18347.1-29880.1) | 25018.5(18962.8-31147.7) | 0.17(0.13, 0.2) | 147.2(56.5-306.1) | 155.1(58.8-316.1) | 0.18(0.15, 0.21) |
| Solomon Islands | 21245.7(14021.6-28614.4) | 5139(2841.4-8108.9) | -4.42(-4.57, -4.28) | 132.9(48.2-292) | 31.7(10.3-72.3) | -4.46(-4.59, -4.33) |
| Somalia | 31103.3(22357.1-39508) | 17545.9(10289.5-25885.1) | -1.81(-1.88, -1.76) | 193.5(74.5-408.6) | 108.1(35.5-239.9) | -1.87(-1.92, -1.82) |
| South Africa | 21957.8(14429.5-29512.8) | 16863.1(11213.4-23217.4) | -0.86(-0.9, -0.82) | 136.4(50.7-294.6) | 103.4(36.6-224.7) | -0.92(-0.96, -0.88) |
| South Sudan | 38310.8(30817.2-45670.6) | 25867.8(16986.3-34213.8) | -1.27(-1.3, -1.24) | 235.5(90.9-486.1) | 157.7(56.5-334.1) | -1.29(-1.32, -1.27) |
| Spain | 18771.3(12181.9-25717) | 6083.9(3562.7-9304.4) | -3.87(-4.16, -3.63) | 117.7(42.6-261.8) | 37.9(12.6-84.6) | -3.89(-4.1, -3.68) |
| Sri Lanka | 32078.8(25895.7-38431) | 24382.3(17072.2-31656.9) | -0.87(-0.94, -0.8) | 199.4(77.6-409.8) | 149.3(54.2-312.9) | -0.92(-0.99, -0.84) |
| Sudan | 18371.2(11933.7-25130.9) | 20347.3(13644-27197.7) | 0.33(0.33, 0.34) | 114.2(42.9-254.1) | 126.4(47.2-276.6) | 0.33(0.32, 0.34) |
| Suriname | 25494.2(18931.9-32305.9) | 25047.6(18522.5-31723.4) | -0.06(-0.07, -0.05) | 160.3(62.3-336.8) | 155.6(58.2-319.7) | -0.09(-0.11, -0.08) |
| Sweden | 27183.9(19631.4-34572.8) | 24485.7(17177.9-31968.3) | -0.26(-0.32, -0.19) | 170.6(65.6-365.5) | 153.4(56.1-321) | -0.28(-0.33, -0.21) |
| Switzerland | 22290.3(15321.3-28712.8) | 22851.2(15412.8-30333.9) | 0.09(0.07, 0.11) | 139.1(52.9-292.1) | 142.3(51.3-299.9) | 0.08(0.06, 0.1) |
| Syrian Arab Republic | 20312.7(13886.8-26966.7) | 19848.5(13585.1-26360.5) | -0.07(-0.08, -0.07) | 126.8(47.9-274.7) | 122.8(44.5-257.7) | -0.1(-0.11, -0.1) |
| Taiwan (Province of China) | 28054(20896.6-35282.4) | 27966.1(20647.9-35200.9) | -0.01(-0.02, -0.01) | 176.6(67.5-371.4) | 175.6(67.2-373.6) | -0.02(-0.03, -0.02) |
| Tajikistan | 21859.8(15384.9-28479.3) | 15229.9(9779.4-21478.2) | -1.16(-1.22, -1.1) | 137.8(51.8-295.8) | 95.8(35-209.1) | -1.17(-1.22, -1.11) |
| Thailand | 25391.5(18440.1-32404) | 29721.6(23413.3-36015.6) | 0.49(0.47, 0.52) | 158.2(60.2-339.6) | 185(71-368) | 0.52(0.5, 0.54) |
| Timor-Leste | 22944.9(15635.6-30175.3) | 21228.6(14072.2-28309.9) | -0.26(-0.29, -0.24) | 142.2(52.8-309) | 130.9(47-280.6) | -0.3(-0.34, -0.27) |
| Togo | 35409.6(27702.3-43030.1) | 30167.9(21949.6-37871.4) | -0.51(-0.54, -0.5) | 220.7(86.5-458) | 188.1(70.8-384.6) | -0.51(-0.53, -0.5) |
| Tokelau | 23808.6(16765-30906.2) | 6937.2(3829.2-10882.7) | -3.89(-4.07, -3.73) | 148.6(55.5-319.1) | 42.7(13.9-98.1) | -3.92(-4.04, -3.78) |
| Tonga | 23914.3(16977.8-31026.4) | 6548.3(3646.5-10312.4) | -4.12(-4.32, -3.97) | 149.8(56.4-324.3) | 40.5(13.1-91.5) | -4.14(-4.26, -4.02) |
| Trinidad and Tobago | 26217.1(19859-32932.1) | 27408.9(20873.6-34044.7) | 0.15(0.14, 0.16) | 164.3(64-345.2) | 170.3(64.9-347.7) | 0.12(0.11, 0.13) |
| Tunisia | 21925.5(15464-28682.3) | 23355.6(16893.8-29848.5) | 0.21(0.19, 0.24) | 136.8(52.2-296.2) | 143.9(53.7-296) | 0.16(0.14, 0.18) |
| Turkey | 15150.2(9203-21802.5) | 22542.7(16560-28839.1) | 1.31(1.24, 1.4) | 94.7(34.6-216.1) | 139.9(52.8-284.5) | 1.29(1.23, 1.36) |
| Turkmenistan | 23219.4(16573.5-30105.2) | 19744.8(13959.6-26155.8) | -0.5(-0.55, -0.46) | 146(56.2-312.2) | 124.2(45.5-258.3) | -0.51(-0.56, -0.46) |
| Tuvalu | 21939.2(14679.4-29055) | 5858(3154-9305.9) | -4.18(-4.32, -4.06) | 137.3(51.4-298.7) | 36.2(11.5-80.8) | -4.22(-4.36, -4.09) |
| Uganda | 34661.9(26587.9-42492.1) | 26216.3(17366.6-34651.4) | -0.87(-0.92, -0.83) | 215(84.1-446.5) | 162.4(58.2-340.9) | -0.87(-0.91, -0.83) |
| Ukraine | 22405.6(16236-29128.6) | 20310(14281.1-26751.8) | -0.3(-0.32, -0.29) | 139(53.4-294.8) | 126(46.1-262.6) | -0.3(-0.31, -0.28) |
| United Arab Emirates | 25241.7(19403.5-31690.5) | 25973.7(19630.7-32304) | 0.1(0.08, 0.12) | 156(59.9-327.2) | 160(60.4-322.7) | 0.09(0.07, 0.11) |
| United Kingdom | 14631.9(8871.9-21577.9) | 14328.5(9724.6-19658.9) | -0.1(-0.19, 0) | 91.8(32.9-207.3) | 89.3(31.7-194.9) | -0.12(-0.2, -0.03) |
| United Republic of Tanzania | 35799.9(27771.3-43438.3) | 27213.1(18137.4-35617.7) | -0.86(-0.92, -0.82) | 222.6(86.9-461.5) | 168.7(60.8-352.2) | -0.87(-0.91, -0.83) |
| United States of America | 24289.9(17896.6-31052.2) | 23899.8(18689.3-29134.6) | -0.12(-0.18, -0.05) | 150.9(57.7-321.6) | 145.8(55.8-302.1) | -0.18(-0.24, -0.12) |
| United States Virgin Islands | 29536.9(23050.4-36380.7) | 30161.9(23375-36781.7) | 0.05(0.01, 0.08) | 186.1(71.8-386) | 187.5(71.8-380.2) | 0(-0.03, 0.04) |
| Uruguay | 25785.8(19613-32249.7) | 27156.3(20911.1-33578.1) | 0.17(0.15, 0.18) | 162(62.6-339.4) | 169.6(64.5-345.5) | 0.15(0.13, 0.16) |
| Uzbekistan | 23616.3(16576.1-30608.7) | 17700.6(11941.6-24074.7) | -0.9(-0.96, -0.85) | 148.5(55.4-316.8) | 110.8(40.5-234.9) | -0.92(-0.98, -0.86) |
| Vanuatu | 22392.9(15324.8-29653.1) | 5378.3(2958-8465.8) | -4.49(-4.72, -4.33) | 140.3(51.9-305.5) | 33.4(10.8-76.4) | -4.51(-4.66, -4.38) |
| Venezuela (Bolivarian Republic of) | 25679.9(18989.7-32467.9) | 24513.1(17759-31224.4) | -0.14(-0.15, -0.13) | 160.8(61.9-339.7) | 152.6(57.1-312.3) | -0.16(-0.17, -0.15) |
| Viet Nam | 15607.3(9425.4-22947.1) | 23106.1(15497.3-30356.7) | 1.22(1.03, 1.43) | 97.7(35.1-221.7) | 144(51.9-307.1) | 1.15(1.03, 1.27) |
| Yemen | 21465(15224.1-27938.1) | 19318.8(13107.3-25816.3) | -0.34(-0.36, -0.33) | 133(51.1-287.3) | 119.6(43.4-249.8) | -0.35(-0.36, -0.34) |
| Zambia | 36512.3(28808-44036.8) | 28048.5(19094-36500.6) | -0.84(-0.89, -0.8) | 227.3(89-468.2) | 173.2(62.3-369.1) | -0.87(-0.91, -0.83) |
| Zimbabwe | 14638.7(8699.4-22076.2) | 13619.1(7867.9-20861) | -0.22(-0.24, -0.2) | 91.5(31.9-209.6) | 84.5(29.5-193.4) | -0.24(-0.26, -0.22) |

Data in parentheses are 95% confidence intervals.

ASR, age-standardized rate; AAPC, average annual percentage change; DALYs, disability-adjusted life years.

**Table S11**. Difference and decomposition of DALYs from 1990 to 2021 for older adults, globally, by sex, and by social-development index.

| **Location** | **Overall difference** | | **Aging** | **Population** | **Epidemiological change** |
| --- | --- | --- | --- | --- | --- |
| **Both** | |  |  |  |  |
| Global | 3936723 | | 48781.43(1.24%) | 4383906.64(111.36%) | -495965.08(-12.6%) |
| Low SDI | 127671.3 | | 1768.49(1.39%) | 143444.59(112.35%) | -17541.83(-13.74%) |
| Low-middle SDI | 558840.3 | | 7029.73(1.26%) | 603213.16(107.94%) | -51402.58(-9.2%) |
| Middle SDI | 1486734 | | 24785.22(1.67%) | 1556487.27(104.69%) | -94538.37(-6.36%) |
| High-middle SDI | 943057 | | 10951.74(1.16%) | 1057163.2(112.1%) | -125057.95(-13.26%) |
| High SDI | 817296.2 | | 14406.02(1.76%) | 1008546.94(123.4%) | -205656.81(-25.16%) |
| **Male** |  | |  |  |  |
| Global | 1734206 | | 29256.29(1.69%) | 1908322.79(110.04%) | -203372.61(-11.73%) |
| Low SDI | 57402.41 | | 930.7(1.62%) | 65369.99(113.88%) | -8898.28(-15.5%) |
| Low-middle SDI | 236193 | | 2261.14(0.96%) | 260678.15(110.37%) | -26746.26(-11.32%) |
| Middle SDI | 635534.3 | | 11954.14(1.88%) | 668595.16(105.2%) | -45014.98(-7.08%) |
| High-middle SDI | 406943.6 | | 6255.97(1.54%) | 444661.42(109.27%) | -43973.77(-10.81%) |
| High SDI | 396844.3 | | 9332.57(2.35%) | 472138.93(118.97%) | -84627.2(-21.33%) |
| **Female** |  | |  |  |  |
| Global | 2202517 | | 19504.38(0.89%) | 2458297.3(111.61%) | -275285.16(-12.5%) |
| Low SDI | 70268.85 | | 805.72(1.15%) | 78242.42(111.35%) | -8779.29(-12.49%) |
| Low-middle SDI | 322647.3 | | 4605.7(1.43%) | 344025.89(106.63%) | -25984.31(-8.05%) |
| Middle SDI | 851199.8 | | 12117.33(1.42%) | 887235.46(104.23%) | -48152.98(-5.66%) |
| High-middle SDI | 536113.4 | | 5085.99(0.95%) | 602370.66(112.36%) | -71343.27(-13.31%) |
| High SDI | 420451.9 | | 5542.59(1.32%) | 528302.29(125.65%) | -113393.04(-26.97%) |

Abbreviations: DALYs, disability-adjusted life-years; SDI, social-development index.

**Table S12** The predicted prevalence and DALYs for oral diseases up to 2050 globally

|  | **Prevalence** | | **DALYs** | |
| --- | --- | --- | --- | --- |
|  | Case Number (million) | ASR (000s) | Case Number (000s) | ASR |
| 1990 | 211.91(211.78-212.03) | 65.17(65.21-65.13) | 3344.08(3298.1-3390.05) | 1045.99(1060.48-1031.51) |
| 1991 | 217.08(216.95-217.21) | 64.91(64.96-64.87) | 3398.61(3351.37-3445.86) | 1034.1(1048.54-1019.66) |
| 1992 | 222.53(222.4-222.66) | 64.69(64.73-64.64) | 3456.87(3408.3-3505.43) | 1023.01(1037.42-1008.61) |
| 1993 | 228.2(228.06-228.33) | 64.49(64.53-64.45) | 3519.27(3469.34-3569.19) | 1013.13(1027.51-998.76) |
| 1994 | 234.07(233.94-234.21) | 64.34(64.39-64.3) | 3588.67(3537.38-3639.97) | 1005.27(1019.62-990.92) |
| 1995 | 240.06(239.93-240.2) | 64.25(64.29-64.21) | 3666.28(3613.64-3718.91) | 999.93(1014.25-985.61) |
| 1996 | 246.04(245.9-246.18) | 64.16(64.2-64.12) | 3747.93(3693.95-3801.91) | 995.94(1010.23-981.64) |
| 1997 | 251.72(251.58-251.87) | 64.04(64.08-64) | 3826.42(3771.13-3881.71) | 991.7(1005.98-977.43) |
| 1998 | 257.46(257.31-257.6) | 63.92(63.96-63.88) | 3906.66(3850.04-3963.28) | 987.66(1001.92-973.41) |
| 1999 | 263.35(263.2-263.5) | 63.82(63.86-63.78) | 3991.2(3933.22-4049.17) | 984.28(998.52-970.05) |
| 2000 | 269.69(269.54-269.84) | 63.77(63.8-63.73) | 4085.04(4025.68-4144.4) | 981.99(996.2-967.78) |
| 2001 | 276.77(276.62-276.92) | 63.79(63.82-63.75) | 4190.84(4130.03-4251.66) | 981.11(995.29-966.93) |
| 2002 | 284.33(284.18-284.48) | 63.88(63.92-63.84) | 4301.52(4239.22-4363.82) | 980.97(995.12-966.81) |
| 2003 | 292.01(291.85-292.16) | 64(64.04-63.97) | 4412.77(4348.99-4476.54) | 981.02(995.15-966.9) |
| 2004 | 299.56(299.4-299.72) | 64.11(64.15-64.07) | 4520.81(4455.58-4586.04) | 980.42(994.52-966.32) |
| 2005 | 306.88(306.72-307.04) | 64.12(64.15-64.08) | 4625.32(4558.58-4692.06) | 978.22(992.3-964.15) |
| 2006 | 313.48(313.32-313.64) | 63.93(63.97-63.9) | 4731.68(4663.4-4799.96) | 975.3(989.35-961.25) |
| 2007 | 319.25(319.08-319.41) | 63.59(63.63-63.56) | 4842.49(4772.72-4912.26) | 973.01(987.02-959) |
| 2008 | 324.74(324.58-324.91) | 63.23(63.26-63.19) | 4954.42(4883.17-5025.66) | 971.17(985.15-957.19) |
| 2009 | 330.62(330.45-330.78) | 62.94(62.97-62.9) | 5066.35(4993.6-5139.1) | 969.4(983.35-955.45) |
| 2010 | 337.24(337.07-337.41) | 62.79(62.82-62.76) | 5175.72(5101.42-5250.01) | 967.25(981.17-953.32) |
| 2011 | 345.28(345.11-345.45) | 62.71(62.74-62.68) | 5263.48(5187.31-5339.66) | 958.96(972.88-945.04) |
| 2012 | 355.77(355.6-355.94) | 62.61(62.64-62.57) | 5334.33(5255.55-5413.11) | 942.32(956.26-928.39) |
| 2013 | 366.75(366.58-366.93) | 62.55(62.58-62.52) | 5387.32(5305.82-5468.82) | 923.15(937.11-909.19) |
| 2014 | 379.72(379.54-379.9) | 62.5(62.53-62.47) | 5472.89(5388.24-5557.53) | 906.66(920.65-892.68) |
| 2015 | 391.55(391.37-391.74) | 62.49(62.52-62.46) | 5597.75(5510.41-5685.09) | 899.8(913.79-885.82) |
| 2016 | 405.8(405.62-405.99) | 62.48(62.51-62.45) | 5837.91(5747.57-5928.25) | 906.22(920.16-892.28) |
| 2017 | 420.52(420.33-420.71) | 62.49(62.52-62.46) | 6149.88(6056.67-6243.1) | 921.57(935.45-907.69) |
| 2018 | 435.98(435.78-436.17) | 62.51(62.54-62.49) | 6483.01(6386.8-6579.22) | 937.66(951.47-923.84) |
| 2019 | 452.08(451.88-452.28) | 62.52(62.55-62.49) | 6782.75(6683.29-6882.21) | 946.28(960.05-932.51) |
| 2020 | 468.84(468.64-469.04) | 62.7(62.72-62.67) | 7081.67(6979.02-7184.33) | 956.19(969.93-942.46) |
| 2021 | 482.64(482.44-482.84) | 62.72(62.75-62.69) | 7281.1(7175.57-7386.62) | 955.62(969.34-941.9) |
| 2022 | 502.18(491.46-512.9) | 62.9(64.23-61.56) | 7622.92(7325.25-7920.6) | 962.42(999.83-925.02) |
| 2023 | 518.37(503.73-533.01) | 63(64.77-61.22) | 7909.81(7506.43-8313.19) | 968.25(1017.44-919.05) |
| 2024 | 534.62(514.44-554.81) | 63.09(65.47-60.71) | 8205.74(7613.82-8797.65) | 974.17(1044.22-904.12) |
| 2025 | 551.22(524.05-578.39) | 63.19(66.3-60.07) | 8512.92(7692.3-9333.53) | 980.3(1074.51-886.09) |
| 2026 | 568.4(532.84-603.96) | 63.28(67.25-59.32) | 8833.87(7770.79-9896.95) | 986.73(1105.16-868.31) |
| 2027 | 586.43(541.02-631.83) | 63.38(68.3-58.47) | 9171.44(7853.09-10489.79) | 993.56(1136.1-851.03) |
| 2028 | 605.97(549.18-662.75) | 63.49(69.45-57.52) | 9536.2(7938.81-11133.59) | 1000.71(1168.13-833.29) |
| 2029 | 627.1(557.33-696.88) | 63.59(70.68-56.5) | 9931.04(8021.16-11840.93) | 1008.13(1201.89-814.38) |
| 2030 | 649.18(564.81-733.56) | 63.7(71.99-55.41) | 10347.24(8086.17-12608.32) | 1015.91(1237.86-793.97) |
| 2031 | 671.58(571-772.16) | 63.81(73.38-54.24) | 10776.84(8122.6-13431.08) | 1024.15(1276.4-771.9) |
| 2032 | 693.7(575.34-812.06) | 63.94(74.86-53.01) | 11211.91(8120.55-14303.28) | 1032.93(1317.84-748.01) |
| 2033 | 715.93(578.15-853.72) | 64.07(76.42-51.71) | 11659.92(8082.01-15237.84) | 1042.2(1362.27-722.13) |
| 2034 | 738.83(579.8-897.86) | 64.2(78.06-50.35) | 12129.76(8009.15-16250.37) | 1051.94(1409.78-694.11) |
| 2035 | 761.96(579.88-944.05) | 64.35(79.78-48.92) | 12614.9(7893.34-17336.46) | 1062.23(1460.56-663.9) |
| 2036 | 784.91(578-991.83) | 64.51(81.59-47.44) | 13109.09(7726.75-18491.43) | 1073.15(1514.86-631.45) |
| 2037 | 807.26(573.79-1040.72) | 64.69(83.49-45.89) | 13606.2(7502.18-19710.23) | 1084.81(1572.97-596.66) |
| 2038 | 829.15(567.39-1090.9) | 64.88(85.49-44.27) | 14110.79(7219.26-21002.31) | 1097.21(1635.06-559.35) |
| 2039 | 851.04(559.09-1142.98) | 65.09(87.57-42.6) | 14631.33(6878.37-22384.29) | 1110.36(1701.36-519.36) |
| 2040 | 872.93(548.82-1197.04) | 65.31(89.76-40.86) | 15167.09(6473.57-23860.61) | 1124.33(1772.17-476.5) |
| 2041 | 894.84(536.47-1253.2) | 65.55(92.05-39.05) | 15717.57(5998.49-25436.65) | 1139.22(1847.89-430.56) |
| 2042 | 916.75(521.94-1311.57) | 65.81(94.45-37.17) | 16282.63(5446.25-27119) | 1155.13(1928.98-381.27) |
| 2043 | 938.66(505.15-1372.17) | 66.1(96.97-35.23) | 16864.45(4811.27-28917.64) | 1172.1(2015.88-328.32) |
| 2044 | 960.73(486.17-1435.3) | 66.4(99.6-33.2) | 17468.58(4087.76-30849.41) | 1190.2(2109.08-271.33) |
| 2045 | 983.28(464.99-1501.56) | 66.73(102.36-31.1) | 18100.03(3266.66-32933.4) | 1209.53(2209.17-209.89) |
| 2046 | 1006.62(441.59-1571.66) | 67.09(105.26-28.92) | 18764.5(2349.4-35192.29) | 1230.17(2316.84-144.51) |
| 2047 | 1031.1(415.83-1646.38) | 67.48(108.31-26.65) | 19468.35(1458.12-37652.84) | 1252.26(2432.9-85.01) |
| 2048 | 1057.29(387.67-1726.9) | 67.89(111.5-24.28) | 20223.88(758.2-40356.26) | 1275.89(2558.22-41.78) |
| 2049 | 1085.19(356.82-1813.56) | 68.34(114.86-21.81) | 21035.88(319.82-43331.96) | 1301.17(2693.77-16.22) |
| 2050 | 1114.33(322.84-1905.82) | 68.82(118.4-19.24) | 21899.57(36.7-46592.32) | 1328.24(2840.72-1.57) |

Data in parentheses are 95% confidence intervals.

ASR, age-standardized rate; DALYs, disability-adjusted life years.


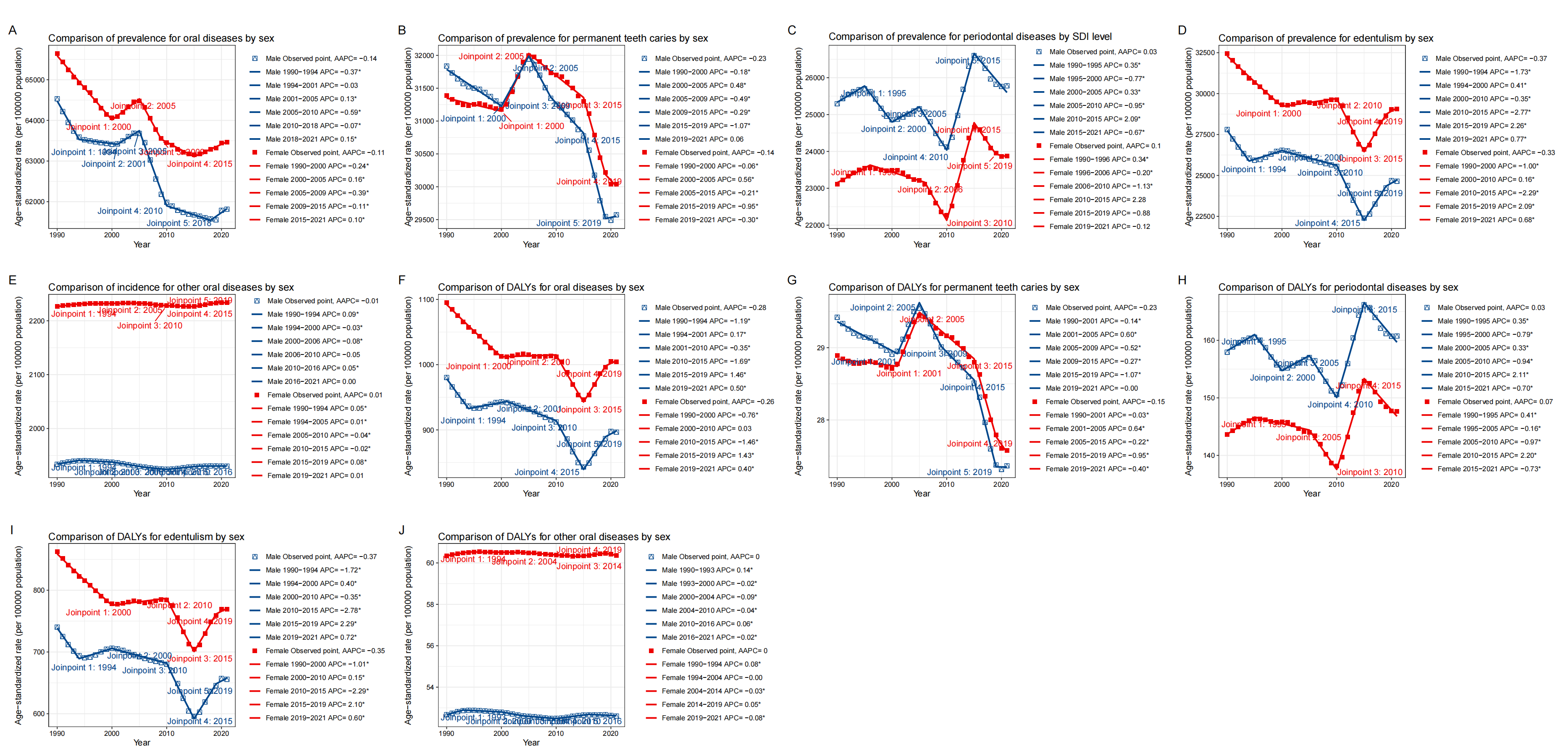


Figure S1. Joinpoint regression analysis of global prevalence for oral diseases (A), permanent teeth caries (B), periodontal diseases(C), edentulism (D), other oral diseases (E), and DALYs for oral diseases (F), permanent teeth caries (G), periodontal diseases(H), edentulism (I), and other oral diseases (J) from 1990 to 2021 among different sexes.

AAPC, average annual percentage change; APC, annual percentage change; DALYs, disability-adjusted life-years; *P < 0.05.


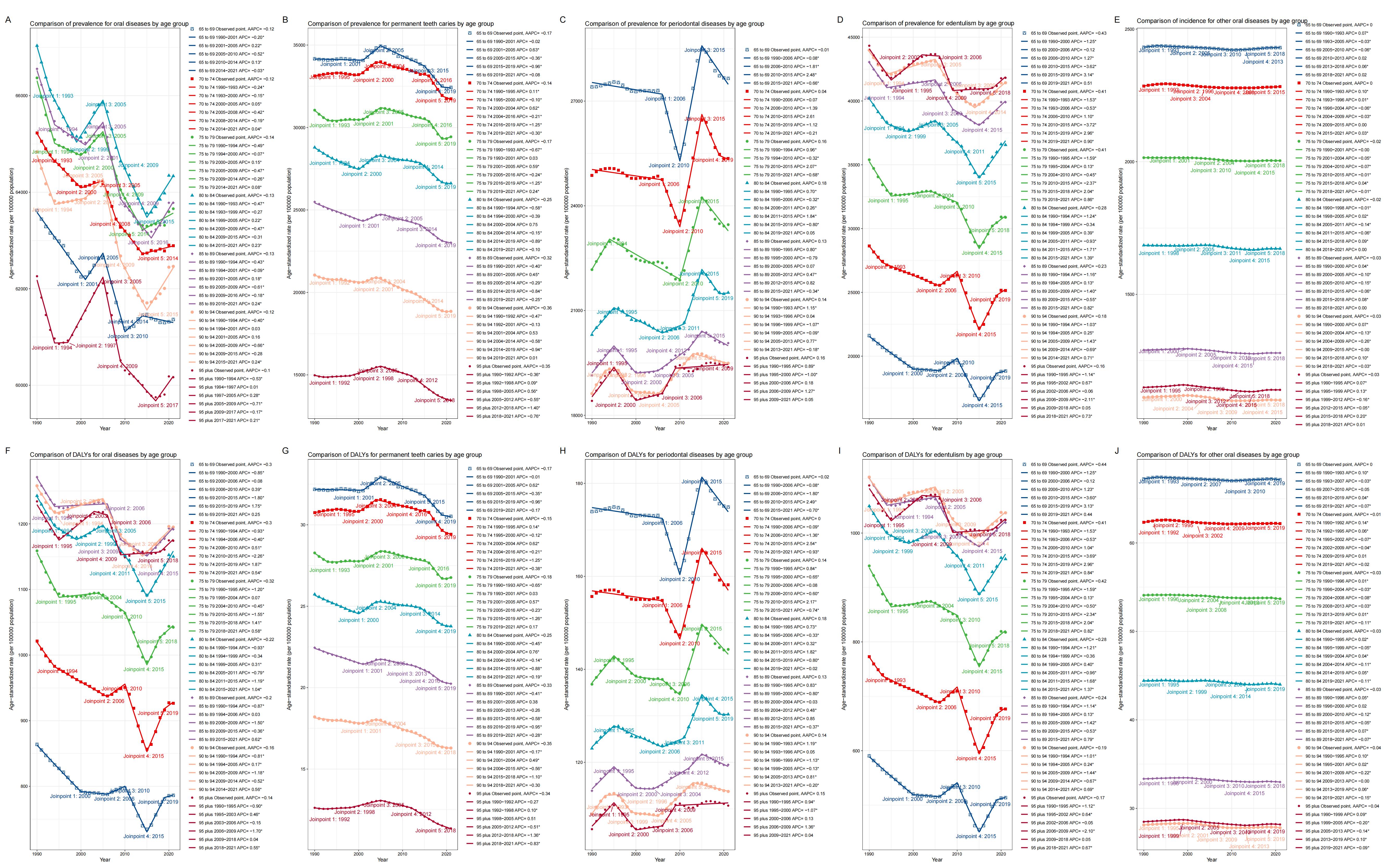


Figure S2. Joinpoint regression analysis of global prevalence for oral diseases (A), permanent teeth caries (B), periodontal diseases(C), edentulism (D), other oral diseases (E), and DALYs for oral diseases (F), permanent teeth caries (G), periodontal diseases(H), edentulism (I), and other oral diseases (J) from 1990 to 2021 among different age groups.

APC, annual percentage change; AAPC, average annual percentage change; DALYs, disability-adjusted life-years; *P < 0.05.


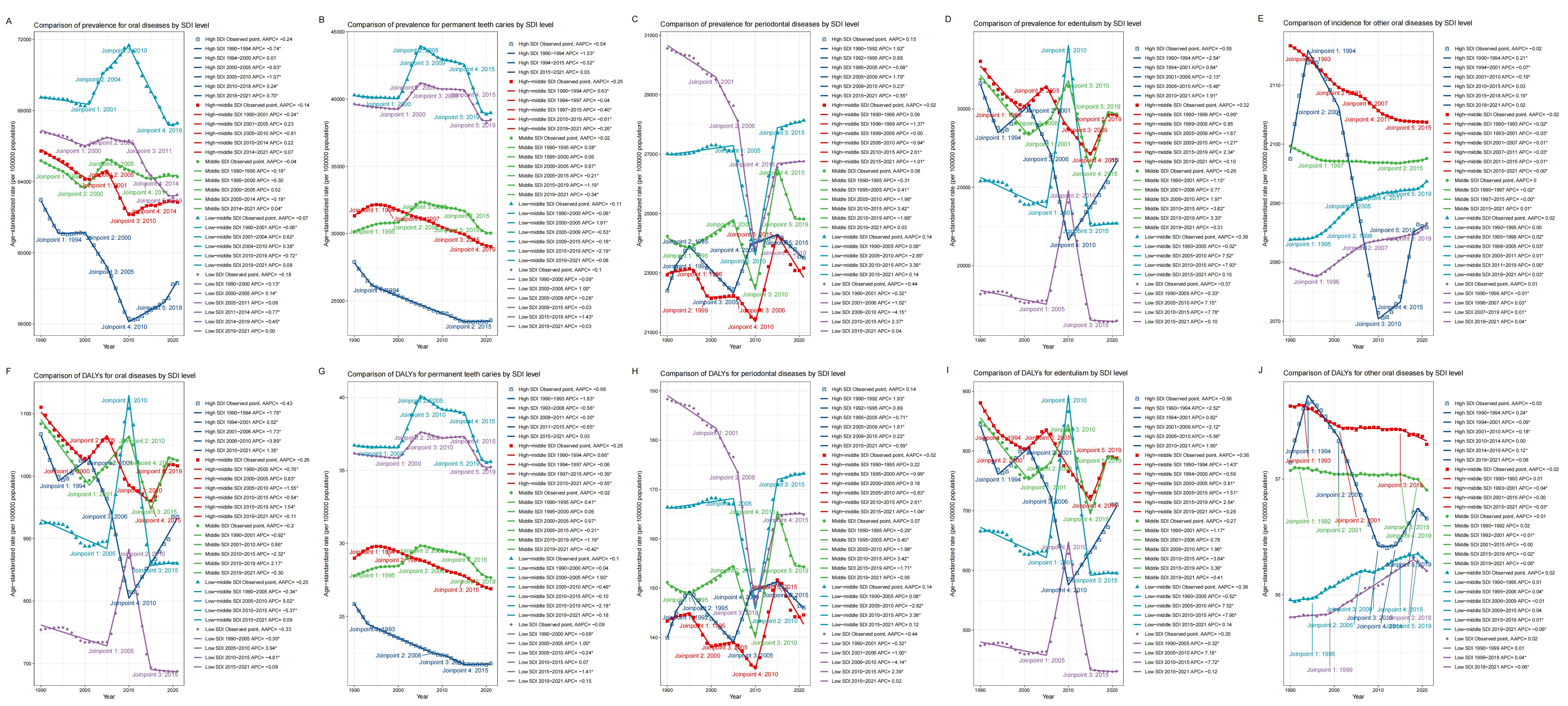


Figure S3. Joinpoint regression analysis of global prevalence for oral diseases (A), permanent teeth caries (B), periodontal diseases(C), edentulism (D), other oral diseases (E), and DALYs for oral diseases (F), permanent teeth caries (G), periodontal diseases(H), edentulism (I), and other oral diseases (J) from 1990 to 2021 among different SDI level.

APC, annual percentage change; AAPC, average annual percentage change; DALYs, disability-adjusted life-years; SDI, social-development index; *P < 0.05.


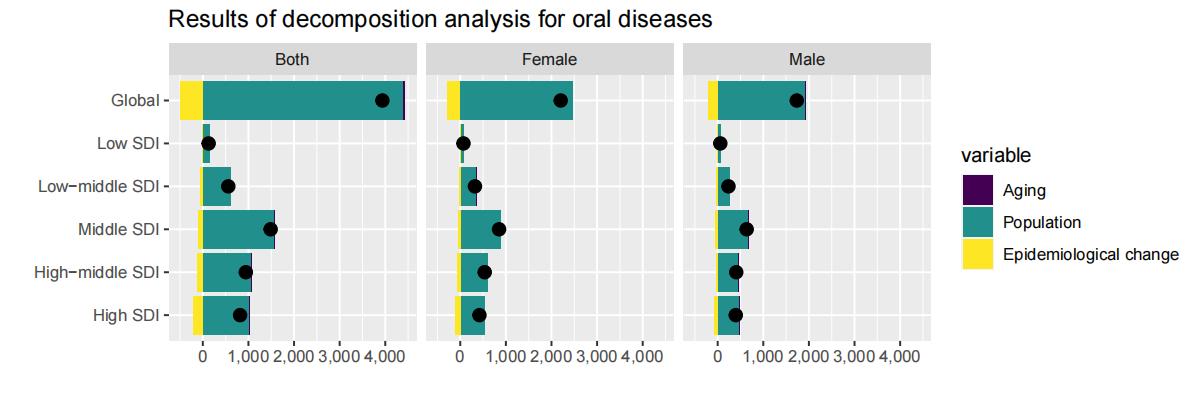


Figure S4. Results of decomposition analysis of DALYs for oral diseases from 1990 to 2021 at the global level, by sex, and by SDI regions.

DALYs, disability-adjusted life-years; SDI, social-development index.
