# Supplementary material for: Global, Regional, and National Burden of Oral Diseases in Older Adults Aged 65 Years And Over
Source: Int Dent J. 2025 Dec 9;76(1):109297. doi: 10.1016/j.identj.2025.109297 (PMC12753236; doi:10.1016/j.identj.2025.109297)
Supplement: Supplementary file 2 [file mmc2.pdf]

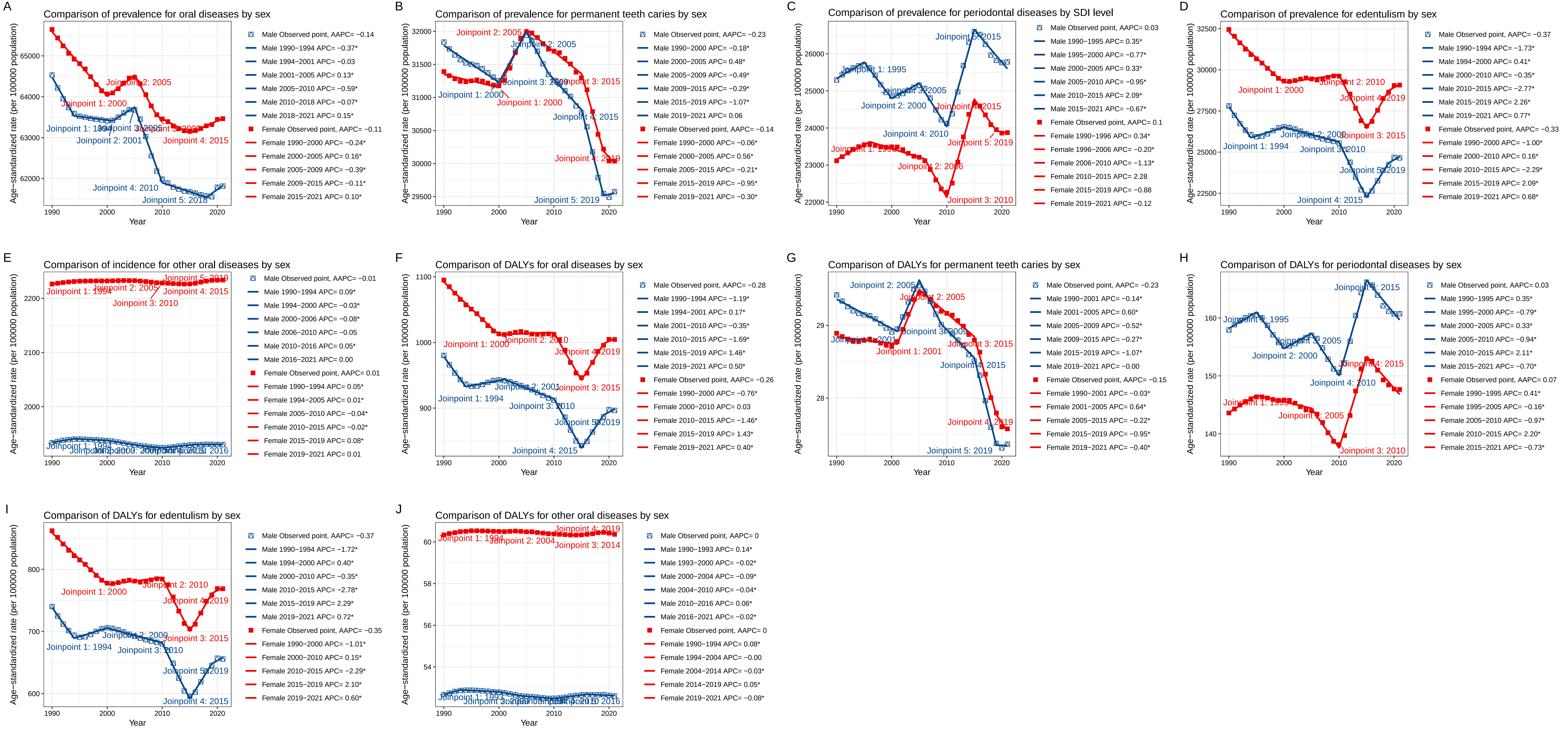
A
Comparison of prevalence for oral diseases by sex
Male Observed point, AAPC= −0.14
Male 1990−1994 APC= −0.37*
Male 1994−2001 APC= −0.03
Male 2001−2005 APC= 0.13*
Male 2005−2010 APC= −0.59*
Male 2010−2018 APC= −0.07*
Male 2018−2021 APC= 0.15*
Female Observed point, AAPC= −0.11
Female 1990−2000 APC= −0.24*
Female 2000−2005 APC= 0.16*
Female 2005−2009 APC= −0.39*
Female 2009−2015 APC= −0.11*
Female 2015−2021 APC= 0.10*
B
Comparison of prevalence for permanent teeth caries by sex
Male Observed point, AAPC= −0.23
Male 1990−2000 APC= −0.18*
Male 2000−2005 APC= 0.48*
Male 2005−2009 APC= −0.49*
Male 2009−2015 APC= −0.29*
Male 2015−2019 APC= −1.07*
Male 2019−2021 APC= 0.06
Female Observed point, AAPC= −0.14
Female 1990−2000 APC= −0.06*
Female 2000−2005 APC= 0.56*
Female 2005−2015 APC= −0.21*
Female 2015−2019 APC= −0.95*
Female 2019−2021 APC= −0.30*
C
Comparison of prevalence for periodontal diseases by SDI level
Male Observed point, AAPC= 0.03
Male 1990−1995 APC= 0.35*
Male 1995−2000 APC= −0.77*
Male 2000−2005 APC= 0.33*
Male 2005−2010 APC= −0.95*
Male 2010−2015 APC= 2.09*
Male 2015−2021 APC= −0.67*
Female Observed point, AAPC= 0.1
Female 1990−1996 APC= 0.34*
Female 1996−2006 APC= −0.20*
Female 2006−2010 APC= −1.13*
Female 2010−2015 APC= 2.28
Female 2015−2019 APC= −0.88
Female 2019−2021 APC= −0.12
D
Comparison of prevalence for edentulism by sex
Male Observed point, AAPC= −0.37
Male 1990−1994 APC= −1.73*
Male 1994−2000 APC= 0.41*
Male 2000−2010 APC= −0.35*
Male 2010−2015 APC= −2.77*
Male 2015−2019 APC= 2.26*
Male 2019−2021 APC= 0.77*
Female Observed point, AAPC= −0.33
Female 1990−2000 APC= −1.00*
Female 2000−2010 APC= 0.16*
Female 2010−2015 APC= −2.29*
Female 2015−2019 APC= 2.09*
Female 2019−2021 APC= 0.68*
E
Comparison of incidence for other oral diseases by sex
Male Observed point, AAPC= −0.01
Male 1990−1994 APC= 0.09*
Male 1994−2000 APC= −0.03*
Male 2000−2006 APC= −0.08*
Male 2006−2010 APC= −0.05
Male 2010−2016 APC= 0.05*
Male 2016−2021 APC= 0.00
Female Observed point, AAPC= 0.01
Female 1990−1994 APC= 0.05*
Female 1994−2005 APC= 0.01*
Female 2005−2010 APC= −0.04*
Female 2010−2015 APC= −0.02*
Female 2015−2019 APC= 0.08*
Female 2019−2021 APC= 0.01
F
Comparison of DALYs for oral diseases by sex
Male Observed point, AAPC= −0.28
Male 1990−1994 APC= −1.19*
Male 1994−2001 APC= 0.17*
Male 2001−2010 APC= −0.35*
Male 2010−2015 APC= −1.69*
Male 2015−2019 APC= 1.46*
Male 2019−2021 APC= 0.50*
Female Observed point, AAPC= −0.26
Female 1990−2000 APC= −0.76*
Female 2000−2010 APC= 0.03
Female 2010−2015 APC= −1.46*
Female 2015−2019 APC= 1.43*
Female 2019−2021 APC= 0.40*
G
Comparison of DALYs for permanent teeth caries by sex
Male Observed point, AAPC= −0.23
Male 1990−2001 APC= −0.14*
Male 2001−2005 APC= 0.60*
Male 2005−2009 APC= −0.52*
Male 2009−2015 APC= −0.27*
Male 2015−2019 APC= −1.07*
Male 2019−2021 APC= −0.00
Female Observed point, AAPC= −0.15
Female 1990−2001 APC= −0.03*
Female 2001−2005 APC= 0.64*
Female 2005−2015 APC= −0.22*
Female 2015−2019 APC= −0.95*
Female 2019−2021 APC= −0.40*
H
Comparison of DALYs for periodontal diseases by sex
Male Observed point, AAPC= 0.03
Male 1990−1995 APC= 0.35*
Male 1995−2000 APC= −0.79*
Male 2000−2005 APC= 0.33*
Male 2005−2010 APC= −0.94*
Male 2010−2015 APC= 2.11*
Male 2015−2021 APC= −0.70*
Female Observed point, AAPC= 0.07
Female 1990−1995 APC= 0.41*
Female 1995−2005 APC= −0.16*
Female 2005−2010 APC= −0.97*
Female 2010−2015 APC= 2.20*
Female 2015−2021 APC= −0.73*
I
Comparison of DALYs for edentulism by sex
Male Observed point, AAPC= −0.37
Male 1990−1994 APC= −1.72*
Male 1994−2000 APC= 0.40*
Male 2000−2010 APC= −0.35*
Male 2010−2015 APC= −2.78*
Male 2015−2019 APC= 2.29*
Male 2019−2021 APC= 0.72*
Female Observed point, AAPC= −0.35
Female 1990−2000 APC= −1.01*
Female 2000−2010 APC= 0.15*
Female 2010−2015 APC= −2.29*
Female 2015−2019 APC= 2.10*
Female 2019−2021 APC= 0.60*
J
Comparison of DALYs for other oral diseases by sex
Male Observed point, AAPC= 0
Male 1990−1993 APC= 0.14*
Male 1993−2000 APC= −0.02*
Male 2000−2004 APC= −0.09*
Male 2004−2010 APC= −0.04*
Male 2010−2016 APC= 0.06*
Male 2016−2021 APC= −0.02*
Female Observed point, AAPC= 0
Female 1990−1994 APC= 0.08*
Female 1994−2004 APC= −0.00
Female 2004−2014 APC= −0.03*
Female 2014−2019 APC= 0.05*
Female 2019−2021 APC= −0.08*
Age−standardized rate (per 100000 population)
Year
