# Supplementary figures and images for: Global, Regional, and National Burden of Oral Diseases in Older Adults Aged 65 Years And Over
Source: Int Dent J. 2025 Dec 9;76(1):109297. doi: 10.1016/j.identj.2025.109297 (PMC12753236; doi:10.1016/j.identj.2025.109297)

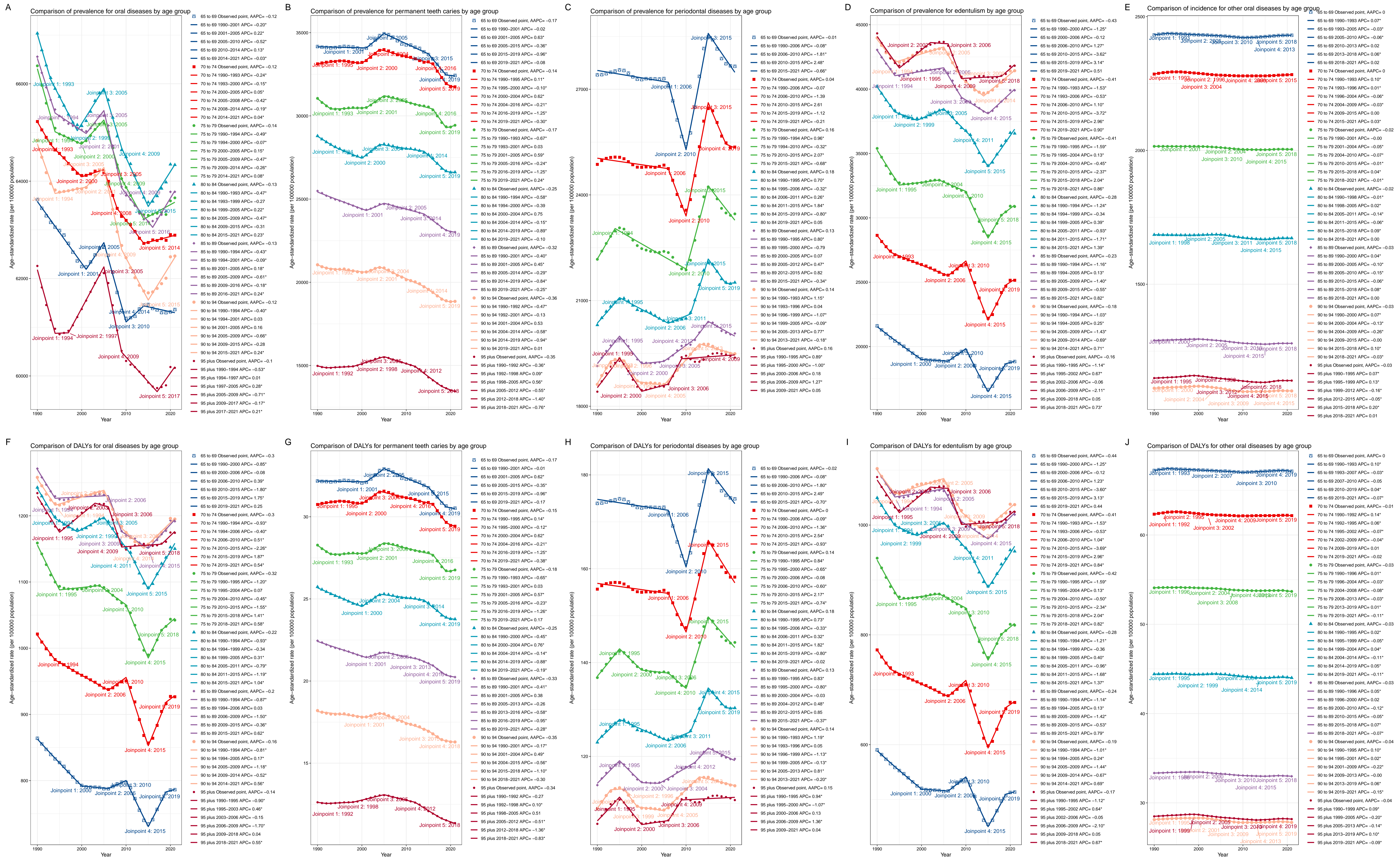

Supplement: Supplementary file 3 [file mmc3.pdf]

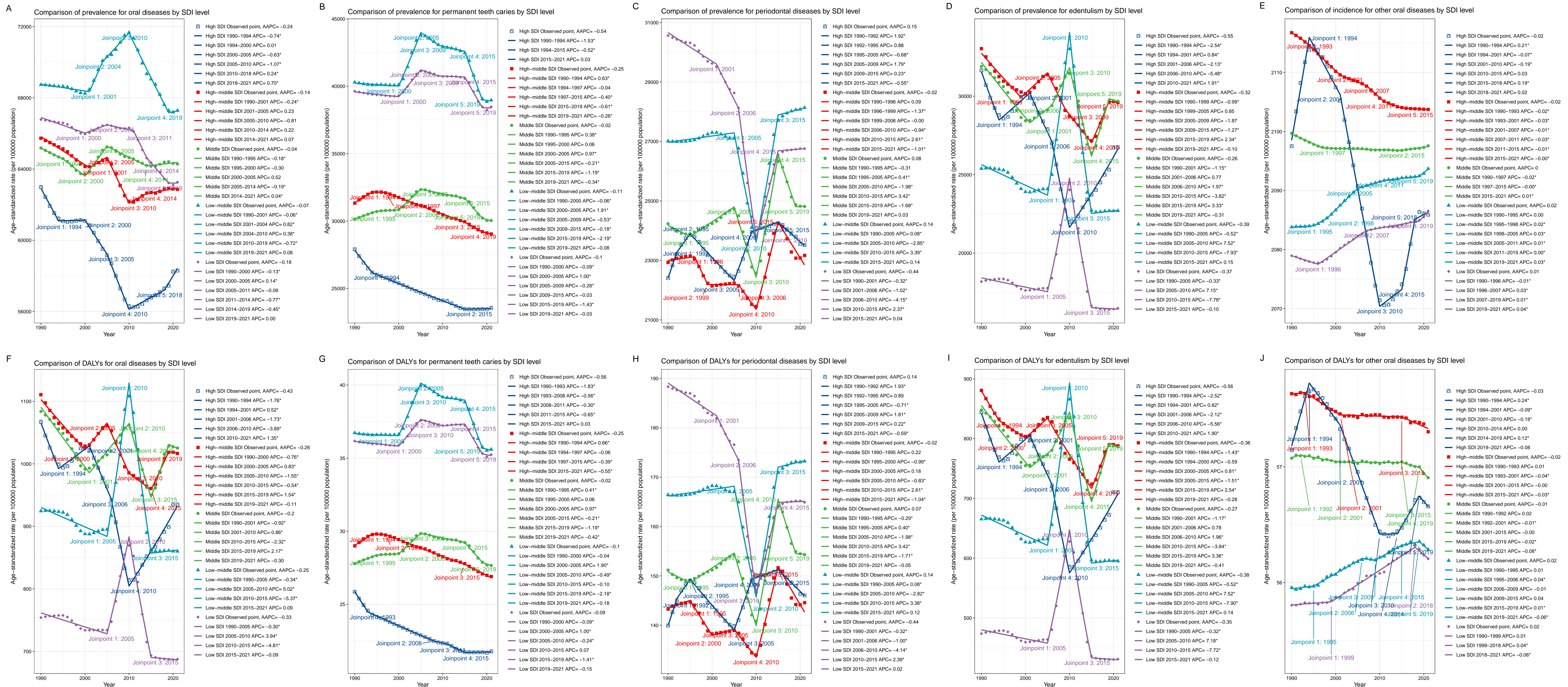

Supplement: Supplementary file 4 [file mmc4.pdf]

# Results of decomposition analysis for oral diseases

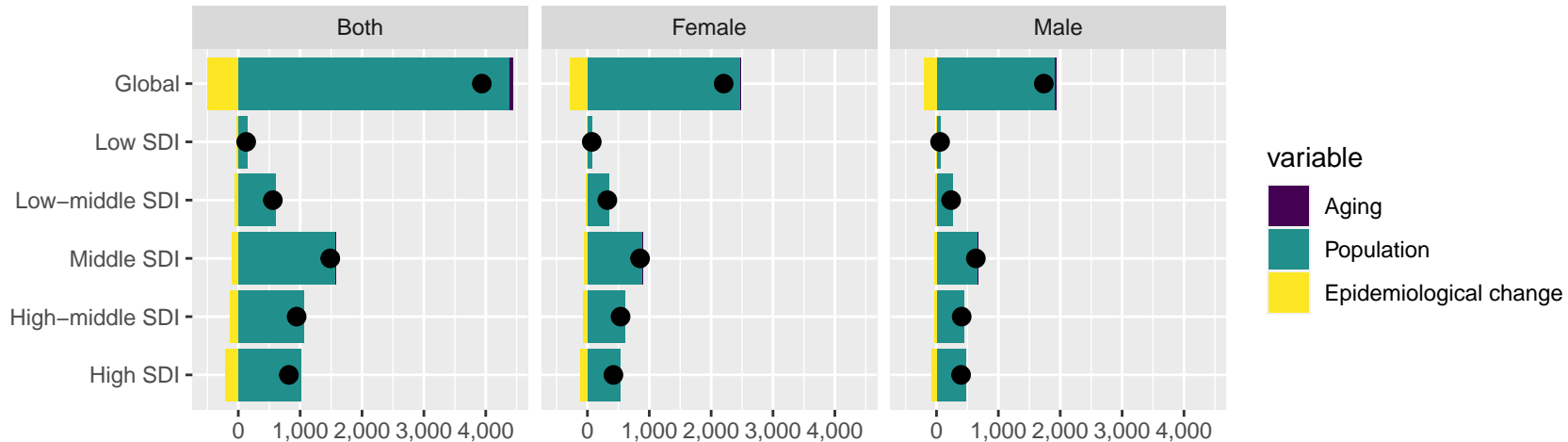

Supplement: Supplementary file 5 [file mmc5.pdf]
